# Supplementary material for: HUWE1 is a critical colonic tumour suppressor gene that prevents MYC signalling, DNA damage accumulation and tumour initiation
Source: EMBO Mol Med. 2016 Dec 22;9(2):181–97. doi: 10.15252/emmm.201606684 (PMC5286368; doi:10.15252/emmm.201606684)
Supplement: Supplementary file 1 — Appendix [file EMMM-9-181-s001.pdf]

Appendix

Supplemental experimental procedures

Table S1

Table S2

Supplemental Experimental Procedures

Primary antibodies

The following primary antibodies were used for immunohistochemistry: β-catenin (1:50; Transduction Laboratories), BrdUrd (1:500; BD Biosciences), c-MYC (1:200; Santa Cruz sc-764), Caspase 3 (1:800; R&D Systems AF835), Lysozyme (1:200; DAKO A0099), MMP7 (1:200; Santa Cruz sc-8832), OLFM4 (1:200, Abcam ab85046) and γH2AX (1:2500; Upstate 05636).

Western blotting

Primary antibodies used for Western blotting were as follows: γH2AX (1:200; Cell Signalling Technologies 9718), MYC (1:100; Cell Signaling Technology 5605), MCL1 (1:500; Rockland 600-401-394S) H2AX (1:2000; Cell Signaling Technology 7631) and β-actin (1:5000; Cell Signaling Technology 4970).

Apc LOH primers

Apc wt F	GTTCTGTATCATGGAAAGATAGGTGGTC
Apc wt R	CACTCAAAACGCTTTTGAGGGTTGATT
Apc KO F	GTTCTGTATCATGGAAAGATAGGTGGTC
Apc KO R	GAGTACGGGGTCTCTGTCTCAGTGAA

RNA isolation

We isolated RNA from whole tissue pieces using a Qiagen RNeasy Mini Kit (Qiagen, Crawly, West Sussex, UK) according to the manufacturer's instructions. We used DNA-free (Ambion/Applied Biosystems, Warrington, UK) to digest genomic DNA contamination from the RNA samples prior to RT-PCR.

Quantitative PCR

We reverse transcribed 0.5μg of RNA using a DyNAmo SYBR Green 2-step qPCR kit (Finnzymes, Espoo, Finland) in a reaction volume of 20ul. qPCR was performed in duplicate in a 20 μl reaction mixture containing 10 μl of 2 × DyNAmo HS master mix, 0.5 μM of each of the primers and 0.1 μl cDNA. The reaction mixture without a template was run as a control. The cycling conditions were as follows: 95°C for 15 min, followed by 40 cycles of three steps consisting of denaturation at 94°C for 15 s, primer annealing at the optimal temperature for 30s, and primer extension at 72°C for 30s. β-actin was used to normalize for differences in RNA input.

cBioportal data analysis

cBioportal data was analysed using the available search function. For determining *HUWE1* mutation rate a simple search was carried out for HUWE1 either with the cancer type section left empty or selecting Colorectal Adenocarcinoma (TCGA, Nature 2012) or Colorectal Adenocarcinoma (Genentech, Nature 2012). For mutual exclusivity analysis the following search criteria were used:

Colorectal Adenocarcinoma (TCGA, Nature 2012)/ Tumors with sequencing and CNA data (212)/User-defined List/2 genes, mRNA Expression z-Scores (RNA Seq RPKM)

HUWE1

MYC: AMP GAIN EXP>2

Tumour culture

For Western blot analysis we cultured individual tumours to remove contaminating stromal tissue. Individual tumours were isolated, chopped into small pieces and washed with PBS, then PBS + 1mM EDTA. Tumours were then digested with 5% Trypsin for 30min at 37°C. Digestion was stopped by addition of 5% FBS and cells isolated by passing through a 40µm filter. Cells were pelleted at 300g and washed 3 times with Advanced DMEM/F12 media + Pen/Strep + L-glutamine + Hepes. Single cells were resuspended in matrigel and plated in a 24 well plate (25µl / well). 500 µl media supplemented with N2, B27, EGF (50ng/ml) and Noggin (100ng/ml) was added. Spheres were passaged every 3-4 days for a minimum of 2 weeks to remove contaminating cells.

Image analysis

For nuclear β-catenin analysis intestinal slides stained for ß-catenin were scanned on an SCN400F slide scanner (Leica). The files were exported and analysed in HALO v2.0 image analysis software (Indica Labs). The tumour boarders were manually identified and the resulting tumour areas were segregated using the “classifier” module to identify epithelial cells. CytoNuclear v1.5 analysis was carried out to identify and quantify nuclear positive epithelial cells. Thresholds and parameters were set manually.

Table S1.

Gene Symbol	Gene Title	p-value(Hu	qvalue(p-va	Fold-Change(Huwe1 KO
**Lgals6**	lectin, galactose binding, soluble 6	1,03E-06	0,0155407	-97,737
**Gm10499 /// Gm8909**	predicted gene 10499 /// predicted gene 890	3,10E-05	0,0726097	-61,1337
**H2-T3**	histocompatibility 2, T region locus 3	0,0006942	0,0849322	-19,3552
**Slc34a2**	solute carrier family 34 (sodium phosphate), r	0,0001444	0,0831415	-11,3813
**H2-T18 /// H2-T3 /// LOC10**	histocompatibility 2, T region locus 18 /// hist	0,0004742	0,0849322	-10,8219
**H2-T18**	histocompatibility 2, T region locus 18	0,0006172	0,0849322	-7,88783
**Gsta2**	glutathione S-transferase, alpha 2 (Yc2)	0,0001261	0,0805578	-6,90287
**Tmigd1**	transmembrane and immunoglobulin domain	0,0298512	0,243554	-5,8898
**Ddah1**	dimethylarginine dimethylaminohydrolase 1	0,0002266	0,0849322	-5,01368
**Cryz**	crystallin, zeta	0,0003398	0,0849322	-4,87342
**Wdpcp**	WD repeat containing planar cell polarity effe	0,0003048	0,0849322	-4,29598
**Sprr2a1 /// Sprr2a2**	small proline-rich protein 2A1 /// small prolin	0,0057706	0,150585	-4,22789
**Scd1**	stearoyl-Coenzyme A desaturase 1	0,0028046	0,124286	-4,11411
**---**	---	0,0019685	0,114711	-4,11282
**Ddah1**	dimethylarginine dimethylaminohydrolase 1	0,0011405	0,0968114	-3,82635
**Fut2**	fucosyltransferase 2	0,0440907	0,272416	-3,62498
**Ddah1**	dimethylarginine dimethylaminohydrolase 1	0,0007096	0,0849322	-3,51672
**Hspa1b**	heat shock protein 1B	0,018445	0,212189	-3,51284
**Acot6**	acyl-CoA thioesterase 6	0,0001318	0,0805578	-3,48896
**Paqr7**	progestin and adipoQ receptor family membe	0,0234486	0,227833	-3,39741
**Bcl2l15**	BCLl2-like 15	0,01067	0,181461	-3,3333
**Paqr8**	progestin and adipoQ receptor family membe	0,0085473	0,169883	-3,27393
**5430410E06Rik**	RIKEN cDNA 5430410E06 gene	0,0024577	0,120058	-3,25517
**H2-K1**	Histocompatibility 2, K1, K region	0,0014104	0,103881	-3,2252
**Mboat1**	membrane bound O-acyltransferase domain c	0,0227844	0,225899	-3,15455
**Npl**	N-acetylneuraminate pyruvate lyase	0,0183529	0,212171	-3,15435
**Igf2**	insulin-like growth factor 2	0,0023157	0,119793	-3,15258
**Tspan12**	tetraspanin 12	0,0091005	0,17254	-3,15063
**Cryz**	crystallin, zeta	0,0062013	0,153771	-3,11286
**Sqrdl**	sulfide quinone reductase-like (yeast)	0,008216	0,169111	-3,08814
**Ear12 /// Ear2 /// Ear3**	eosinophil-associated, ribonuclease A family,	0,0014789	0,104066	-3,05865
**H2-T18 /// H2-T3 /// H2-T3-l**	histocompatibility 2, T region locus 18 /// hist	0,0051949	0,14802	-3,00127
**Serpinb5**	serine (or cysteine) peptidase inhibitor, clade	0,0497946	0,282091	-2,98913
**Adamts4**	a disintegrin-like and metallopeptidase (repro	0,0051459	0,147369	-2,92373
**Paqr8**	progestin and adipoQ receptor family membe	0,0087622	0,170577	-2,89824
**Fst**	follistatin	0,0036452	0,132023	-2,8663
**Scd1**	stearoyl-Coenzyme A desaturase 1	0,0089727	0,171687	-2,81649
**Pigl**	phosphatidylinositol glycan anchor biosynthes	6,77E-05	0,0726097	-2,77852
**Krt12**	keratin 12	0,0219451	0,223509	-2,76459
**Clca2**	chloride channel calcium activated 2	0,0015887	0,105787	-2,71003
**---**	---	0,0007136	0,0849322	-2,70592
**Afm**	afamin	0,0253435	0,231521	-2,69487
**Serpinb5**	serine (or cysteine) peptidase inhibitor, clade	0,0050423	0,146373	-2,60552
**Bglap /// Bglap-rs1 /// Bglap**	bone gamma carboxyglutamate protein /// bo	0,0196463	0,215905	-2,59842
**Nipal2**	NIPA-like domain containing 2	0,0002314	0,0849322	-2,59551
**Anxa10**	annexin A10	0,0498026	0,282091	-2,59325
**Ccl28**	chemokine (C-C motif) ligand 28	0,001434	0,103881	-2,59184
**S100a14**	S100 calcium binding protein A14	0,0439254	0,271976	-2,57668
**Serpinb5**	serine (or cysteine) peptidase inhibitor, clade	0,0230467	0,226843	-2,57544
**Nbea**	neurobeachin	0,0005098	0,0849322	-2,56888
**Gas5**	growth arrest specific 5	0,0005989	0,0849322	-2,546

Table S1.

**Angptl4**	angiopoietin-like 4	0,0290568	0,24078	-2,53693
**Slc5a8**	solute carrier family 5 (iodide transporter), me	0,0362388	0,258831	-2,5167
**Ighm**	Immunoglobulin heavy constant mu	0,0322166	0,251226	-2,51091
**H2-Ea-ps**	histocompatibility 2, class II antigen E alpha, p	0,0278623	0,237798	-2,47887
**5830477G23Rik**	RIKEN cDNA 5830477G23 gene	0,0118737	0,186339	-2,45407
**Cyp4a10 /// Cyp4a31**	cytochrome P450, family 4, subfamily a, polyp	0,0021308	0,117205	-2,43751
**Paqr8**	progestin and adipoQ receptor family membe	0,0117936	0,185884	-2,43539
**Tcf23**	transcription factor 23	0,0181546	0,211065	-2,43392
**Lpl**	lipoprotein lipase	0,0013627	0,103163	-2,42047
**Chst4**	carbohydrate (chondroitin 6/keratan) sulfotra	0,0227066	0,225899	-2,406
**Gkn3**	gastrokine 3	0,0042348	0,1385	-2,39984
**1190003J15Rik**	RIKEN cDNA 1190003J15 gene	3,31E-05	0,0726097	-2,39012
**Ccl28**	chemokine (C-C motif) ligand 28	0,0026284	0,122879	-2,37616
**Ccdc21**	coiled-coil domain containing 21	0,0089292	0,171277	-2,37282
**Gm3579**	predicted gene 3579	0,0135961	0,192995	-2,37075
**Pla2g5**	phospholipase A2, group V	0,0005926	0,0849322	-2,36881
**Il1rn**	interleukin 1 receptor antagonist	0,0015569	0,104987	-2,35744
**---**	---	0,0048519	0,14496	-2,34325
**Alox15**	arachidonate 15-lipoxygenase	0,0197756	0,216393	-2,33624
**A730091E23Rik**	RIKEN cDNA A730091E23 gene	0,0003276	0,0849322	-2,29534
**Amph**	amphiphysin	0,0023596	0,119793	-2,28775
**Paqr8**	progestin and adipoQ receptor family membe	0,0237617	0,228298	-2,28207
**Ivd**	isovaleryl coenzyme A dehydrogenase	0,0013932	0,103789	-2,26468
**1700029I01Rik /// Gm13139**	RIKEN cDNA 1700029I01 gene /// predicted ge	0,0127594	0,189985	-2,25087
**1190003J15Rik**	RIKEN cDNA 1190003J15 gene	0,0037283	0,132928	-2,24832
**Haus2**	HAUS augmin-like complex, subunit 2	0,022045	0,22382	-2,24405
**Ldhb**	lactate dehydrogenase B	0,045811	0,275912	-2,24215
**Zdhhc2**	zinc finger, DHHC domain containing 2	0,0037039	0,132893	-2,24205
**Akr1b8**	aldo-keto reductase family 1, member B8	0,0269169	0,235593	-2,24155
**Ighm**	Immunoglobulin heavy constant mu	0,032579	0,251771	-2,23493
**Zdhhc2**	zinc finger, DHHC domain containing 2	0,0054631	0,148673	-2,23244
**Lpl**	lipoprotein lipase	0,0051947	0,14802	-2,22897
**Slc25a4**	solute carrier family 25 (mitochondrial carrier	0,0008346	0,0864886	-2,21086
**Il1rn**	interleukin 1 receptor antagonist	0,0012491	0,0999478	-2,20863
**Agphd1**	aminoglycoside phosphotransferase domain c	0,0005966	0,0849322	-2,19867
**Gas5**	growth arrest specific 5	0,0064433	0,1551	-2,19663
**Capg**	capping protein (actin filament), gelsolin-like	0,0268211	0,235425	-2,1905
**Tnfrsf11b**	tumor necrosis factor receptor superfamily, m	0,0081221	0,168173	-2,14788
**Enpp4**	ectonucleotide pyrophosphatase/phosphodie	0,035336	0,257842	-2,14471
**Clca2**	chloride channel calcium activated 2	0,0132009	0,191132	-2,13209
**Wdpcp**	WD repeat containing planar cell polarity effe	0,0433648	0,271071	-2,12933
**Sprr1a**	small proline-rich protein 1A	0,0373561	0,260842	-2,12806
**Ypel2**	yippee-like 2 (Drosophila)	0,0200238	0,216847	-2,11129
**Casp4**	caspase 4, apoptosis-related cysteine peptida	0,0029054	0,125052	-2,10917
**Sord**	sorbitol dehydrogenase	0,0445384	0,273295	-2,10452
**Vnn1**	vanin 1	0,0039927	0,135934	-2,09395
**Adm**	adrenomedullin	0,0072424	0,160297	-2,06718
**Acer1**	alkaline ceramidase 1	0,0017478	0,109295	-2,05852
**Arntl2**	aryl hydrocarbon receptor nuclear translocato	0,0089156	0,171117	-2,00512
**Ctgf**	connective tissue growth factor	0,0120288	0,186803	-1,99434
**Zfp811**	zinc finger protein 811	0,0094184	0,174332	-1,99385
**C87414 /// Gm3259 /// Gm6**	expressed sequence C87414 /// predicted ger	0,0159811	0,202582	-1,9918

Table S1.

**Ddah1**	dimethylarginine dimethylaminohydrolase 1	0,0043416	0,139788	-1,9913
**Tmem161a**	transmembrane protein 161A	0,0005125	0,0849322	-1,9856
**Timp1**	tissue inhibitor of metalloproteinase 1	0,0202477	0,217253	-1,98517
**Slit2**	slit homolog 2 (Drosophila)	0,0324094	0,251402	-1,97882
**Nfil3**	nuclear factor, interleukin 3, regulated	0,0061549	0,153771	-1,97075
**Mpp7**	membrane protein, palmitoylated 7 (MAGUK	0,0081057	0,168173	-1,95869
**Igf2**	insulin-like growth factor 2	0,0304422	0,245075	-1,95552
**Cilp**	cartilage intermediate layer protein, nucleotic	0,0404061	0,266997	-1,94734
**Rpl17**	ribosomal protein L17	0,0096804	0,176573	-1,94337
**Stom**	stomatin	0,0016014	0,105804	-1,94045
**Fgf18**	fibroblast growth factor 18	0,0180361	0,210593	-1,93743
**Il22ra1**	interleukin 22 receptor, alpha 1	0,0002641	0,0849322	-1,92917
**Pof1b**	premature ovarian failure 1B	0,0038757	0,134489	-1,92496
**Gmds**	GDP-mannose 4, 6-dehydratase	0,0065014	0,155617	-1,92465
**S100a4**	S100 calcium binding protein A4	0,0235356	0,228001	-1,92354
**Rnf157**	ring finger protein 157	0,003595	0,131572	-1,92297
**Slc11a2**	solute carrier family 11 (proton-coupled divale	0,0088196	0,170577	-1,92279
**Acadl**	acyl-Coenzyme A dehydrogenase, long-chain	0,0003565	0,0849322	-1,91978
**Meis2**	Meis homeobox 2	0,0216903	0,222436	-1,91591
**Grem1**	gremlin 1	0,0342675	0,255872	-1,90452
**Ptms**	parathymosin	0,0492679	0,281592	-1,90411
**Eda2r**	ectodysplasin A2 receptor	0,0112681	0,18408	-1,90178
**Sfrp1**	secreted frizzled-related protein 1	0,0484949	0,27988	-1,90088
**Serpinb5**	serine (or cysteine) peptidase inhibitor, clade	0,0126401	0,189766	-1,89961
**Gm13242 /// LOC10086245[illegible]**	predicted gene 13242 /// zinc finger protein 1	0,0118856	0,186347	-1,89394
**Vwc2**	von Willebrand factor C domain containing 2	0,0444824	0,273253	-1,89126
**Ccl8 /// LOC100503254**	chemokine (C-C motif) ligand 8 /// c-C motif c	0,0409922	0,267381	-1,87348
**Mreg**	melanoregulin	0,0357665	0,258379	-1,8653
**Arl13b**	ADP-ribosylation factor-like 13B	0,002599	0,122478	-1,86451
**Tmem161a**	transmembrane protein 161A	0,0103982	0,179738	-1,86367
**Slc25a4**	solute carrier family 25 (mitochondrial carrier	0,0005883	0,0849322	-1,86187
**Myl7**	myosin, light polypeptide 7, regulatory	0,0015979	0,105787	-1,86118
**Atf7ip2**	activating transcription factor 7 interacting pr	0,0281328	0,238244	-1,85654
**Mga**	MAX gene associated	0,001316	0,102302	-1,85214
**Car8**	carbonic anhydrase 8	0,0014558	0,104066	-1,85154
**Arntl**	aryl hydrocarbon receptor nuclear translocato	0,0005699	0,0849322	-1,85062
**---**	---	0,0096904	0,176658	-1,84954
**B3gnt6**	UDP-GlcNAc:betaGal beta-1,3-N-acetylglucosa	0,0052007	0,14802	-1,84792
**Cyp1b1**	cytochrome P450, family 1, subfamily b, polyp	0,0001521	0,0831415	-1,84609
**Oxct1**	3-oxoacid CoA transferase 1	0,0042169	0,138073	-1,83428
**Vnn1**	vanin 1	0,0148117	0,198344	-1,83423
**Gmds**	GDP-mannose 4, 6-dehydratase	0,0049586	0,145735	-1,83156
**Slc25a4**	solute carrier family 25 (mitochondrial carrier	0,0024816	0,120341	-1,83082
**Oxct1**	3-oxoacid CoA transferase 1	0,0035385	0,131563	-1,829
**Il1rn**	interleukin 1 receptor antagonist	0,0072947	0,16098	-1,82635
**Car8**	carbonic anhydrase 8	0,0213233	0,221191	-1,8212
**B330016D10Rik**	RIKEN cDNA B330016D10 gene	0,0124373	0,189368	-1,81723
**Mpp7**	membrane protein, palmitoylated 7 (MAGUK	0,0281523	0,238244	-1,8139
**Khdrbs3**	KH domain containing, RNA binding, signal tra	0,000419	0,0849322	-1,81087
**Uap1l1**	UDP-N-acteylglucosamine pyrophosphorylase	0,0126923	0,189766	-1,80746
**Ttr**	transthyretin	0,0073166	0,161207	-1,80363
**Oxct1**	3-oxoacid CoA transferase 1	0,01071	0,181488	-1,79548

Table S1.

**Ccdc122**	coiled-coil domain containing 122	0,0073452	0,161334	-1,79408
**Ivd**	isovaleryl coenzyme A dehydrogenase	0,0201242	0,216849	-1,7882
**Ttr**	transthyretin	0,040544	0,267148	-1,7869
**Fam115c**	family with sequence similarity 115, member	0,0097996	0,176669	-1,78389
**Map3k6**	mitogen-activated protein kinase kinase kinas	0,0090948	0,17254	-1,78311
**Dner**	delta/notch-like EGF-related receptor	0,0266997	0,235425	-1,7822
**Gjb1**	gap junction protein, beta 1	0,002337	0,119793	-1,77559
**Rpap1**	RNA polymerase II associated protein 1	0,0409701	0,267335	-1,77163
**2410006H16Rik**	RIKEN cDNA 2410006H16 gene	0,0008247	0,0864886	-1,76949
**Sfrp1**	secreted frizzled-related protein 1	0,0074881	0,162506	-1,76876
**Fam129c**	family with sequence similarity 129, member	0,0228513	0,226196	-1,76818
**7530414M10Rik /// LOC100!**	RIKEN cDNA 7530414M10 gene /// uncharact	0,0030307	0,125899	-1,76279
**Adamts4**	a disintegrin-like and metallopeptidase (repro	0,0359769	0,258387	-1,7616
**Nek8**	NIMA (never in mitosis gene a)-related expres	0,0116702	0,1854	-1,7593
**Fblim1**	filamin binding LIM protein 1	0,0052986	0,14802	-1,75495
**Rpl13a**	ribosomal protein L13A	0,0017667	0,10964	-1,75291
**Cfhr2**	complement factor H-related 2	0,0168655	0,207129	-1,7471
**Ano3**	anoctamin 3	0,0320752	0,250786	-1,74572
**Gkn3**	gastrokine 3	0,0185167	0,21244	-1,74524
**---**	---	0,0251077	0,231021	-1,74521
**Snhg6**	small nucleolar RNA host gene (non-protein c	0,0107934	0,182345	-1,74444
**1500012F01Rik**	RIKEN cDNA 1500012F01 gene	0,0008476	0,0864886	-1,74415
**Cyp1b1**	cytochrome P450, family 1, subfamily b, polyp	0,0068936	0,159107	-1,74408
**Rps25**	ribosomal protein S25	0,0023375	0,119793	-1,74285
**Ddit4**	DNA-damage-inducible transcript 4	0,0020913	0,116965	-1,74262
**---**	---	0,0087799	0,170577	-1,74193
**Oxct1**	3-oxoacid CoA transferase 1	0,0028317	0,124774	-1,7406
**---**	---	0,0070912	0,160168	-1,7394
**D130020L05Rik**	RIKEN cDNA D130020L05 gene	0,017543	0,209167	-1,73873
**Ccl28**	chemokine (C-C motif) ligand 28	0,0007304	0,0849322	-1,73856
**Hspa8**	heat shock protein 8	0,0102037	0,178809	-1,73613
**Tyro3**	TYRO3 protein tyrosine kinase 3	0,0202644	0,217253	-1,73607
**Dleu2**	deleted in lymphocytic leukemia, 2	4,82E-05	0,0726097	-1,73567
**Stom**	stomatin	0,0038572	0,134301	-1,73352
**Ccdc32**	coiled-coil domain containing 32	0,0106205	0,181075	-1,7291
**Bok**	BCL2-related ovarian killer protein	0,0013198	0,102302	-1,72747
**Dab1**	disabled 1	0,0035521	0,131563	-1,72187
**Satl1**	spermidine/spermine N1-acetyl transferase-lil	0,0118111	0,185922	-1,72098
**Ereg**	epiregulin	0,0209726	0,220008	-1,71581
**Gm17586**	predicted gene, 17586	0,0033353	0,130662	-1,71431
**Zdhhc2**	zinc finger, DHHC domain containing 2	0,004422	0,140705	-1,71182
**BC021614**	cDNA sequence BC021614	0,0028533	0,124893	-1,71
**Stom**	stomatin	0,0081257	0,168173	-1,70935
**Ttr**	transthyretin	0,0125237	0,189441	-1,70788
**Zdhhc2**	zinc finger, DHHC domain containing 2	0,0251593	0,231021	-1,70572
**4930581F22Rik**	RIKEN cDNA 4930581F22 gene	0,0156484	0,201591	-1,702
**Svip**	small VCP/p97-interacting protein	0,0044676	0,141005	-1,70026
**Cebpd**	CCAAT/enhancer binding protein (C/EBP), delt	0,0005614	0,0849322	-1,69963
**1810011O10Rik**	RIKEN cDNA 1810011O10 gene	0,0302746	0,244647	-1,69928
**Sfi1**	Sfi1 homolog, spindle assembly associated (ye	0,032806	0,252214	-1,69617
**Senp8**	SUMO/sentrin specific peptidase 8	0,0102344	0,178809	-1,69271
**---**	---	0,0036167	0,131572	-1,69061

Table S1.

**1110038B12Rik**	RIKEN cDNA 1110038B12 gene	0,0009279	0,0874427	-1,69015
**Mpp7**	membrane protein, palmitoylated 7 (MAGUK	0,0100936	0,178211	-1,68989
**St5**	suppression of tumorigenicity 5	0,0027075	0,123955	-1,68986
**Elovl7**	ELOVL family member 7, elongation of long ch	0,0153693	0,200277	-1,68889
**Rbm12b**	RNA binding motif protein 12B	0,0158754	0,202412	-1,68808
**Stom**	stomatin	0,006072	0,152756	-1,6859
**Rpusd2**	RNA pseudouridylate synthase domain contai	0,001911	0,114047	-1,68535
**Ccr3**	chemokine (C-C motif) receptor 3	0,0451667	0,274885	-1,68534
**Capg**	capping protein (actin filament), gelsolin-like	0,0297957	0,243554	-1,68457
**Samd4**	sterile alpha motif domain containing 4	0,0414283	0,268297	-1,68232
**Gtf3c3**	general transcription factor IIIC, polypeptide 3	0,0038601	0,134301	-1,68187
**Igfbp5**	insulin-like growth factor binding protein 5	0,0487197	0,280277	-1,68176
**Efha2**	EF-hand domain family, member A2	0,0108566	0,18246	-1,68009
**4933403F05Rik**	RIKEN cDNA 4933403F05 gene	0,0168289	0,206991	-1,67941
**Ptpn22**	protein tyrosine phosphatase, non-receptor ty	0,0070995	0,160198	-1,6792
**Vgll3**	vestigial like 3 (Drosophila)	0,0394811	0,264976	-1,6751
**Enpp4**	ectonucleotide pyrophosphatase/phosphodie	0,0414916	0,268297	-1,67502
**Parp3**	poly (ADP-ribose) polymerase family, member	0,0005135	0,0849322	-1,67355
**Ypel2**	yippee-like 2 (Drosophila)	0,0386091	0,263865	-1,67122
**Cep55**	centrosomal protein 55	0,0011521	0,0971952	-1,6694
**Pglyrp1**	peptidoglycan recognition protein 1	0,0200838	0,216849	-1,66724
**---**	---	0,0224207	0,225067	-1,66714
**Parp3**	poly (ADP-ribose) polymerase family, member	0,0006339	0,0849322	-1,66637
**Lmna**	lamin A	0,003484	0,131563	-1,66534
**---**	---	0,0485394	0,27988	-1,6651
**Stom**	stomatin	0,0048619	0,14496	-1,66492
**Sqrdl**	sulfide quinone reductase-like (yeast)	0,0436625	0,271676	-1,65936
**Agphd1**	aminoglycoside phosphotransferase domain c	0,010757	0,181907	-1,65818
**Gas2**	growth arrest specific 2	0,0208893	0,219868	-1,65625
**Mob2**	MOB kinase activator 2	0,0129842	0,190262	-1,65451
**Bag4**	BCL2-associated athanogene 4	0,002342	0,119793	-1,65357
**Smcp**	sperm mitochondria-associated cysteine-rich	0,0092685	0,173659	-1,65277
**---**	---	0,0226742	0,225899	-1,65248
**Gm3650 /// Spg11**	predicted gene 3650 /// spastic paraplegia 11	0,0098223	0,176669	-1,65223
**1700040L02Rik**	RIKEN cDNA 1700040L02 gene	0,000733	0,0849322	-1,65195
**Efhd2**	EF hand domain containing 2	0,0133337	0,191798	-1,65066
**Krt222**	keratin 222	0,024492	0,229994	-1,64913
**S100a16**	S100 calcium binding protein A16	0,0005488	0,0849322	-1,64888
**Gm3579**	predicted gene 3579	0,0062203	0,153771	-1,64794
**Gadd45b**	Growth arrest and DNA-damage-inducible 45	0,0416471	0,268297	-1,64641
**Arsa**	arylsulfatase A	0,0310219	0,246737	-1,64611
**Thoc1**	THO complex 1	0,0379534	0,262147	-1,64481
**Stxbp6**	syntaxin binding protein 6 (amisyn)	0,0062345	0,153771	-1,6444
**Nfkbie**	nuclear factor of kappa light polypeptide gene	0,0370204	0,260249	-1,63595
**Thap2**	THAP domain containing, apoptosis associated	0,025993	0,233538	-1,63505
**Rtn4**	reticulon 4	0,0051033	0,146905	-1,6325
**Tufm**	Tu translation elongation factor, mitochondria	0,0125589	0,189455	-1,63241
**Spata5l1**	spermatogenesis associated 5-like 1	0,022064	0,223823	-1,63019
**Zfp799**	zinc finger protein 799	0,013242	0,191394	-1,6295
**4833442J19Rik**	RIKEN cDNA 4833442J19 gene	0,0303702	0,244947	-1,6263
**Acot6**	acyl-CoA thioesterase 6	0,0263474	0,234624	-1,62333
**Nr1h4**	nuclear receptor subfamily 1, group H, memb	0,0065726	0,156088	-1,62071

Table S1.

**Gss**	glutathione synthetase	0,0138368	0,194085	-1,62041
**Dnaja4**	DnaJ (Hsp40) homolog, subfamily A, member	0,0085295	0,169883	-1,61994
**Ppp4r1l-ps**	protein phosphatase 4, regulatory subunit 1-li	0,0074354	0,162116	-1,61882
**Cks2 /// Gm12891 /// Gm19**	CDC28 protein kinase regulatory subunit 2 ///	0,0202713	0,217253	-1,6172
**Sult1c2**	sulfotransferase family, cytosolic, 1C, membe	0,0028601	0,124893	-1,61673
**Pisd-ps1 /// Pisd-ps3**	phosphatidylserine decarboxylase, pseudoger	0,0231124	0,226903	-1,61609
**Cnot7**	CCR4-NOT transcription complex, subunit 7	0,0212058	0,220911	-1,61532
**4831440E17Rik**	RIKEN cDNA 4831440E17 gene	0,0195479	0,215725	-1,61472
**4833438C02Rik**	RIKEN cDNA 4833438C02 gene	0,0067381	0,157295	-1,61439
**Id4**	inhibitor of DNA binding 4	0,0004017	0,0849322	-1,61395
**Ceacam10**	carcinoembryonic antigen-related cell adhesic	0,0428888	0,270312	-1,61256
**D730005E14Rik**	RIKEN cDNA D730005E14 gene	0,0003956	0,0849322	-1,61179
**Rerg**	RAS-like, estrogen-regulated, growth-inhibito	0,0112089	0,18408	-1,61009
**Pla2g4c**	phospholipase A2, group IVC (cytosolic, calciu	0,0150474	0,198997	-1,60961
**Etf1**	eukaryotic translation termination factor 1	0,0223897	0,22504	-1,60923
---	---	0,0168371	0,207014	-1,60799
**Baiap2l1**	BAI1-associated protein 2-like 1	0,0207483	0,219228	-1,60673
**Atg9b**	autophagy related 9B	0,0141816	0,195177	-1,60628
**Stard3nl**	STARD3 N-terminal like	0,0026967	0,123955	-1,60497
**A930035D04Rik**	RIKEN cDNA A930035D04 gene	0,0024874	0,120341	-1,60407
**Dab1**	disabled 1	0,0004699	0,0849322	-1,59956
**Neurog3**	neurogenin 3	0,0305214	0,245142	-1,59896
**Btbd19**	BTB (POZ) domain containing 19	0,0097532	0,176669	-1,5984
**Eif2a**	eukaryotic translation initiation factor 2A	0,0060388	0,152626	-1,59725
**Anxa13**	annexin A13	0,0237066	0,228298	-1,59707
**Clca4**	chloride channel calcium activated 4	0,0033149	0,130662	-1,5968
**Snhg12**	small nucleolar RNA host gene 12	0,0255768	0,232165	-1,59632
---	---	0,0354789	0,258176	-1,59544
**Acn9**	ACN9 homolog (S. cerevisiae)	0,0113072	0,18408	-1,59535
**Jmjd7 /// Pla2g4b**	jumonji domain containing 7 /// phospholipas	0,0016956	0,108097	-1,59534
**6330416G13Rik**	RIKEN cDNA 6330416G13 gene	0,0284113	0,238843	-1,59381
**Dio2**	deiodinase, iodothyronine, type II	0,0080665	0,168164	-1,59213
**Pcdhb9**	protocadherin beta 9	0,0497838	0,282091	-1,59146
**1810011O10Rik**	RIKEN cDNA 1810011O10 gene	0,0110471	0,183576	-1,58988
**Klhl13**	kelch-like 13 (Drosophila)	0,0174819	0,209122	-1,58903
**Esr1**	estrogen receptor 1 (alpha)	0,001256	0,0999478	-1,58867
**Aagab**	alpha- and gamma-adaptin binding protein	0,0363013	0,258995	-1,58671
---	---	0,0247836	0,230738	-1,58373
**Trmt1**	TRM1 tRNA methyltransferase 1 homolog (S. (	0,0109765	0,183113	-1,58251
**Isl1**	ISL1 transcription factor, LIM/homeodomain	0,0378711	0,261835	-1,58162
**2500002B13Rik**	RIKEN cDNA 2500002B13 gene	0,0164325	0,204812	-1,58155
**Fars2**	phenylalanine-tRNA synthetase 2 (mitochondi	0,0459594	0,275912	-1,58126
**Ppp1r14d**	protein phosphatase 1, regulatory (inhibitor) s	0,0083701	0,169445	-1,58106
**Lmna**	lamin A	0,0073783	0,161409	-1,57965
**Gm15848**	predicted gene 15848	0,0057658	0,150585	-1,57895
**Zmym1**	zinc finger, MYM domain containing 1	0,0160976	0,203169	-1,57788
**5730559C18Rik**	RIKEN cDNA 5730559C18 gene	0,0368366	0,259922	-1,5773
**S100a16**	S100 calcium binding protein A16	0,0030251	0,125899	-1,57632
**Arsj**	arylsulfatase J	0,018882	0,213232	-1,57471
**Prodh2**	proline dehydrogenase (oxidase) 2	7,62E-05	0,0732731	-1,57468
**Igf1**	insulin-like growth factor 1	0,0398768	0,265913	-1,57296
**Slc25a12**	solute carrier family 25 (mitochondrial carrier	0,0004933	0,0849322	-1,57253

Table S1.

---	---	0,0114738	0,185214	-1,57139
**Nup54**	nucleoporin 54	0,0197476	0,216218	-1,57138
**Prss23**	protease, serine, 23	0,0336811	0,25456	-1,57138
**A430033K04Rik**	RIKEN cDNA A430033K04 gene	0,002906	0,125052	-1,57124
**Cks2 /// Gm12891 /// Gm19**	CDC28 protein kinase regulatory subunit 2 ///	0,0007736	0,0864886	-1,57049
**Rras**	Harvey rat sarcoma oncogene, subgroup R	0,0156508	0,201591	-1,56993
**Bet1**	blocked early in transport 1 homolog (S. cerev	0,0021004	0,116965	-1,56956
**Guca1a**	guanylate cyclase activator 1a (retina)	0,0134591	0,192379	-1,56939
**Slc11a2**	solute carrier family 11 (proton-coupled divale	0,0445395	0,273295	-1,56923
**Nap1l1**	nucleosome assembly protein 1-like 1	0,0114651	0,185214	-1,56892
**Atf7ip2**	activating transcription factor 7 interacting pr	0,0003849	0,0849322	-1,56852
**Guk1**	guanylate kinase 1	0,0048298	0,14496	-1,56716
**Tufm**	Tu translation elongation factor, mitochondria	0,013078	0,19038	-1,56589
**Tec**	tec protein tyrosine kinase	0,0009501	0,0874427	-1,5655
**Apobec3**	apolipoprotein B mRNA editing enzyme, catal	0,0027206	0,124091	-1,56412
**Sulf1**	sulfatase 1	0,010215	0,178809	-1,56322
**Pnp /// Pnp2**	purine-nucleoside phosphorylase /// purine-n	0,0300548	0,243988	-1,56311
**Tmem2**	transmembrane protein 2	0,0070708	0,160034	-1,56244
**Gsn**	gelsolin	0,0016485	0,107182	-1,56194
**Slc5a3**	solute carrier family 5 (inositol transporters),	0,0018331	0,112105	-1,56171
**Pisd /// Pisd-ps3**	phosphatidylserine decarboxylase /// phospha	0,0029412	0,125632	-1,56099
---	---	0,0071929	0,160198	-1,56036
**C430048L16Rik**	RIKEN cDNA C430048L16 gene	0,0086715	0,170433	-1,56033
**Nap1l1**	nucleosome assembly protein 1-like 1	0,0035769	0,131572	-1,55776
**Sh2d4a**	SH2 domain containing 4A	0,0170219	0,207725	-1,55717
**Dclk1**	doublecortin-like kinase 1	0,0037352	0,132928	-1,55639
**Cyp27a1**	cytochrome P450, family 27, subfamily a, poly	0,0481622	0,279615	-1,55546
**Etv5**	ets variant gene 5	0,0044705	0,141005	-1,5551
**Serhl**	serine hydrolase-like	0,0179935	0,21057	-1,55397
**Gm3417 /// Gm3448 /// Tct**	predicted gene 3417 /// predicted gene 3448	0,0490523	0,28108	-1,55326
---	---	0,0009518	0,0874427	-1,5529
**Fcamr**	Fc receptor, IgA, IgM, high affinity	0,0369961	0,260249	-1,55233
**Habp4**	hyaluronic acid binding protein 4	0,0009427	0,0874427	-1,55115
---	---	0,0030053	0,125847	-1,54977
**Senp6**	SUMO/sentrin specific peptidase 6	0,0028899	0,125052	-1,54729
---	---	0,0030334	0,125899	-1,54726
**Gm4285**	predicted gene 4285	0,0003372	0,0849322	-1,54696
**Kif20b**	kinesin family member 20B	0,0345129	0,256475	-1,54622
**Slc35b2**	solute carrier family 35, member B2	0,0334909	0,253664	-1,54619
**2810442I21Rik**	RIKEN cDNA 2810442I21 gene	0,0111453	0,183909	-1,54494
**Eif4b**	eukaryotic translation initiation factor 4B	0,007046	0,159985	-1,54459
**Pisd /// Pisd-ps3**	phosphatidylserine decarboxylase /// phospha	0,0046916	0,144067	-1,5425
**Tmx2**	thioredoxin-related transmembrane protein 2	0,0295813	0,242925	-1,54087
**Gp2**	glycoprotein 2 (zymogen granule membrane)	0,0175009	0,209122	-1,5407
**Col12a1**	collagen, type XII, alpha 1	0,0132918	0,191689	-1,53915
**Coro7**	coronin 7	0,0055942	0,149218	-1,53878
**1810049H13Rik**	RIKEN cDNA 1810049H13 gene	0,0269799	0,235813	-1,53869
**Isx**	intestine specific homeobox	0,0179654	0,210369	-1,53852
**Uba3**	ubiquitin-like modifier activating enzyme 3	0,0481941	0,279615	-1,53812
**A330033J07Rik**	RIKEN cDNA A330033J07 gene	0,0043065	0,139213	-1,53798
---	---	0,0468477	0,277263	-1,53681
**Vps18**	vacuolar protein sorting 18 (yeast)	0,0012318	0,0998479	-1,53572

Table S1.

**Ccm2**	cerebral cavernous malformation 2	0,0130099	0,190262	-1,53554
**Fabp6**	fatty acid binding protein 6, ileal (gastrotropir	0,0430277	0,270442	-1,53286
**Mast4**	microtubule associated serine/threonine kina	0,0277742	0,237798	-1,53077
**Cd300lb**	CD300 antigen like family member B	0,0408557	0,267335	-1,53074
**Hnrnpa1**	heterogeneous nuclear ribonucleoprotein A1	0,016048	0,20288	-1,52903
**Ubxn1**	UBX domain protein 1	0,0018342	0,112105	-1,52873
**Vmp1**	vacuole membrane protein 1	0,0135224	0,19287	-1,52849
**Cln8**	ceroid-lipofuscinosis, neuronal 8	0,0065241	0,155858	-1,52564
**Cd2ap**	CD2-associated protein	0,0007178	0,0849322	-1,52506
**Cox7a2l**	cytochrome c oxidase subunit VIIa polypeptide	0,0061296	0,153771	-1,52477
**Pebp1**	phosphatidylethanolamine binding protein 1	0,0001537	0,0831415	-1,52475
**1700049G17Rik**	RIKEN cDNA 1700049G17 gene	0,0115006	0,185214	-1,52466
**Gsn**	gelsolin	0,0389818	0,264304	-1,52463
**Ky**	kyphoscoliosis peptidase	0,0175765	0,209395	-1,52331
**Ret**	ret proto-oncogene	0,0023735	0,119793	-1,5228
**Lonrf3**	LON peptidase N-terminal domain and ring fir	0,0146379	0,197577	-1,51982
**A730091E23Rik**	RIKEN cDNA A730091E23 gene	0,0142582	0,19552	-1,51957
**Btbd19**	BTB (POZ) domain containing 19	0,0427096	0,270271	-1,51922
**A230107N01Rik**	RIKEN cDNA A230107N01 gene	0,0049345	0,145521	-1,51768
**Eif2a**	eukaryotic translation initiation factor 2A	0,0394534	0,264936	-1,51768
**Vcan**	versican	0,0162238	0,203684	-1,51652
**Nmnat1**	nicotinamide nucleotide adenylyltransferase 1	0,0009494	0,0874427	-1,51538
**Pigz**	phosphatidylinositol glycan anchor biosynthes	0,0025806	0,122149	-1,5147
**---**	---	0,0110963	0,183584	-1,51392
**7530414M10Rik**	RIKEN cDNA 7530414M10 gene	0,035862	0,258383	-1,51391
**Gsn**	gelsolin	0,0301852	0,244168	-1,51317
**Lamtor2**	late endosomal/lysosomal adaptor, MAPK anc	0,0137853	0,19383	-1,51307
**4930473A06Rik**	RIKEN cDNA 4930473A06 gene	0,0035093	0,131563	-1,51221
**Snhg1**	small nucleolar RNA host gene (non-protein co	0,0234746	0,227891	-1,51189
**1700034H14Rik**	RIKEN cDNA 1700034H14 gene	0,0267846	0,235425	-1,51143
**Tstd1**	thiosulfate sulfurtransferase (rhodanese)-like	0,0027137	0,123955	-1,51138
**Mfap1a /// Mfap1b**	microfibrillar-associated protein 1A /// microf	0,0256519	0,232457	-1,51127
**1810019D21Rik**	RIKEN cDNA 1810019D21 gene	0,0171047	0,208346	-1,51101
**Cfh**	complement component factor h	0,0317664	0,250101	-1,51066
**Atp6v1g2**	ATPase, H+ transporting, lysosomal V1 subuni	0,0259945	0,233538	-1,51011
**Cisd1**	CDGSH iron sulfur domain 1	0,0130249	0,190262	-1,50997
**Srrm1**	Serine/arginine repetitive matrix 1	0,0097351	0,176669	-1,50978
**6030458C11Rik**	RIKEN cDNA 6030458C11 gene	0,0001089	0,0805578	-1,50853
**Snx5**	sorting nexin 5	0,0034133	0,131563	-1,50634
**Smpd4**	sphingomyelin phosphodiesterase 4	0,03862	0,263865	-1,50624
**Cebpd**	CCAAT/enhancer binding protein (C/EBP), delt	0,0012517	0,0999478	-1,50601
**Galntl6**	UDP-N-acetyl-alpha-D-galactosamine:polypep	0,0423846	0,270107	-1,506
**Snx5**	sorting nexin 5	0,0027935	0,124286	-1,50368
**6330578E17Rik**	RIKEN cDNA 6330578E17 gene	0,001675	0,107956	-1,50366
**Syk**	spleen tyrosine kinase	0,0021135	0,116965	-1,50357
**Mctp2**	multiple C2 domains, transmembrane 2	0,0020953	0,116965	-1,50309
**Thpo**	thrombopoietin	0,0453047	0,275246	-1,503
**Pde5a**	phosphodiesterase 5A, cGMP-specific	0,0339761	0,255161	-1,5014
**Nfkbil1**	nuclear factor of kappa light polypeptide gene	0,0095284	0,17517	-1,50093
**Gm3650 /// Spg11**	predicted gene 3650 /// spastic paraplegia 11	0,0101732	0,178783	-1,50062
**Gigyf2**	GRB10 interacting GYF protein 2	0,0286671	0,239623	-1,50028
**Dynlt1a /// Dynlt1b /// Dynl**	dynein light chain Tctex-type 1A /// dynein lig	0,0017397	0,109277	-1,50024

Table S1.

**D8Ertd317e**	DNA segment, Chr 8, ERATO Doi 317, expresse	0,0222443	0,224606	1,50012
**Abca3**	ATP-binding cassette, sub-family A (ABC1), me	0,0389731	0,264304	1,50063
**Ddx58**	DEAD (Asp-Glu-Ala-Asp) box polypeptide 58	0,0101331	0,178366	1,50163
**Slc19a1**	solute carrier family 19 (folate transporter), m	0,0063784	0,154636	1,50196
**Slc39a11**	solute carrier family 39 (metal ion transporter	0,025387	0,231723	1,50272
**Il12rb2**	interleukin 12 receptor, beta 2	0,0097363	0,176669	1,50278
**Agfg1**	ArfGAP with FG repeats 1	0,0035656	0,131572	1,50363
**Aldh4a1**	aldehyde dehydrogenase 4 family, member A:	0,0170683	0,20809	1,50373
**Ces2c /// Ces2d-ps**	carboxylesterase 2C /// carboxylesterase 2D,		0,0236395	0,228264	1,50378
**---**	---	0,0180837	0,210818	1,50382
**Acsl6**	acyl-CoA synthetase long-chain family membe	0,0372362	0,260438	1,50448
**Fam13b**	family with sequence similarity 13, member B	0,0405002	0,267106	1,50517
**Fgl2**	fibrinogen-like protein 2	0,0184453	0,212189	1,50537
**Slc35e3**	solute carrier family 35, member E3	0,0179464	0,210297	1,50571
**Entpd7**	ectonucleoside triphosphate diphosphohydro	0,0020051	0,115014	1,50597
**Sypl2**	synaptophysin-like 2	0,0290929	0,240788	1,50601
**Cc2d2a**	coiled-coil and C2 domain containing 2A	0,0244268	0,229994	1,50626
**Aim1l**	absent in melanoma 1-like	0,0011013	0,0951068	1,50672
**Tmem236**	transmembrane protein 236	0,0084704	0,169849	1,50733
**Ergic2**	ERGIC and golgi 2	0,002527	0,121181	1,50783
**---**	---	0,0021603	0,117467	1,50816
**Osbpl6**	Oxysterol binding protein-like 6	0,0015258	0,104066	1,50932
**Slc22a4**	solute carrier family 22 (organic cation transp	0,0086356	0,170303	1,50947
**Soat2**	sterol O-acyltransferase 2	0,014409	0,196517	1,51166
**Ddx11**	DEAD/H (Asp-Glu-Ala-Asp/His) box polypeptid	0,0424811	0,270264	1,51207
**Ccnj**	cyclin J	0,02745	0,236926	1,51288
**Picalm**	phosphatidylinositol binding clathrin assembly	0,002428	0,120058	1,51306
**Tgm2**	transglutaminase 2, C polypeptide	0,0386315	0,263865	1,51331
**Tbx3**	T-box 3	0,0205539	0,218323	1,51347
**Entpd4 /// LOC100862375**	ectonucleoside triphosphate diphosphohydro	0,0087212	0,170474	1,51406
**Sc4mol**	sterol-C4-methyl oxidase-like	0,0065092	0,155617	1,51464
**Gfi1**	growth factor independent 1	0,0436915	0,271676	1,51466
**Zfp948**	Zinc finger protein 948	0,0299799	0,243601	1,51485
**Tspan6**	tetraspanin 6	0,0013458	0,102833	1,51671
**Slc19a1**	solute carrier family 19 (folate transporter), m	0,00707	0,160034	1,51725
**Pcm1**	pericentriolar material 1	0,0387676	0,264034	1,51758
**Ppard**	peroxisome proliferator activator receptor de	0,0077349	0,165124	1,51764
**---**	---	0,006503	0,155617	1,51861
**Nampt**	nicotinamide phosphoribosyltransferase	0,0246746	0,230449	1,51891
**AI413194 /// Trim56**	expressed sequence AI413194 /// tripartite m	0,0084575	0,169799	1,51919
**---**	---	0,0023619	0,119793	1,51925
**Cycs**	cytochrome c, somatic	0,0088868	0,171066	1,51949
**Slc16a5**	solute carrier family 16 (monocarboxylic acid	0,0090337	0,171976	1,51951
**Hamp2**	hepcidin antimicrobial peptide 2	0,0057923	0,150585	1,51959
**Entpd4 /// LOC100862375**	ectonucleoside triphosphate diphosphohydro	0,0008875	0,0864886	1,52086
**Il33**	interleukin 33	0,0372135	0,260435	1,52095
**Tmem65**	transmembrane protein 65	0,0001285	0,0805578	1,52191
**---**	---	0,0085993	0,170243	1,52246
**Xylb**	xylulokinase homolog (H. influenzae)	0,0077837	0,165518	1,52272
**Defa-rs1**	defensin, alpha, related sequence 1	0,0145436	0,197393	1,52296
**Slc6a7**	solute carrier family 6 (neurotransmitter trans	0,0390759	0,264387	1,52431
**Rab37**	RAB37, member of RAS oncogene family	0,0191346	0,214603	1,52481

Table S1.

**Gls**	glutaminase	0,0012839	0,101183	1,52486
**H2-T10 /// H2-T22 /// H2-T9**	histocompatibility 2, T region locus 10 /// hist	0,0268093	0,235425	1,52502
**Pnpo**	pyridoxine 5'-phosphate oxidase	0,0037329	0,132928	1,52586
**Pcp4l1**	Purkinje cell protein 4-like 1	0,0242772	0,229728	1,52656
**---**	---	0,0032501	0,130026	1,52714
**Iapp**	islet amyloid polypeptide	0,0185028	0,21244	1,52737
**Zfp92**	zinc finger protein 92	0,0291277	0,240788	1,52737
**Als2**	amyotrophic lateral sclerosis 2 (juvenile)	0,0021841	0,117875	1,52762
**Cyp2d22**	cytochrome P450, family 2, subfamily d, polyp	0,0006601	0,0849322	1,52765
**Bnip3**	BCL2/adenovirus E1B interacting protein 3	0,0063167	0,154285	1,52817
**Dpp4**	dipeptidylpeptidase 4	0,0053286	0,14802	1,52942
**Tgm2**	transglutaminase 2, C polypeptide	0,0361874	0,258771	1,52995
**Pcgf5**	polycomb group ring finger 5	0,009451	0,174632	1,53049
**Gclm**	glutamate-cysteine ligase, modifier subunit	0,0037996	0,133272	1,53078
**Sdccag8**	serologically defined colon cancer antigen 8	0,0052307	0,14802	1,53216
**---**	---	0,0195901	0,215794	1,53227
**---**	---	0,0118697	0,186339	1,53319
**A930026I22Rik**	RIKEN cDNA A930026I22 gene	0,0032655	0,130401	1,53331
**Siglec5**	sialic acid binding Ig-like lectin 5	0,0304078	0,245075	1,53376
**Abhd16a**	abhydrolase domain containing 16A	0,0024281	0,120058	1,53445
**Sc5d**	sterol-C5-desaturase (fungal ERG3, delta-5-de	0,0019852	0,114711	1,53484
**H2-Aa**	histocompatibility 2, class II antigen A, alpha	0,0497328	0,282091	1,53602
**Plekhb1**	pleckstrin homology domain containing, famil	0,0055996	0,149218	1,53862
**Cldn15**	claudin 15	0,002768	0,124286	1,53936
**Lce1i**	late cornified envelope 1I	0,023568	0,228041	1,53941
**---**	---	0,0344227	0,256247	1,53956
**Ppargc1a**	peroxisome proliferative activated receptor, g	0,0024587	0,120058	1,53985
**---**	---	0,0002257	0,0849322	1,54061
**Ankrd12**	ankyrin repeat domain 12	0,0030247	0,125899	1,54123
**Vipr1**	vasoactive intestinal peptide receptor 1	0,0171883	0,208575	1,5418
**AY761184**	cDNA sequence AY761184	0,0112418	0,18408	1,54207
**Tmem206**	transmembrane protein 206	0,0207795	0,219436	1,54228
**Sema3b**	sema domain, immunoglobulin domain (Ig), sh	0,0150634	0,198997	1,54282
**Hipk2**	homeodomain interacting protein kinase 2	0,0002913	0,0849322	1,5434
**Mtch2**	mitochondrial carrier homolog 2 (C. elegans)	0,0241457	0,229226	1,54425
**Cdc14a**	CDC14 cell division cycle 14A	0,0227843	0,225899	1,54467
**Naaladl1**	N-acetylated alpha-linked acidic dipeptidase-l	0,0201367	0,216849	1,5448
**Yes1**	Yamaguchi sarcoma viral (v-yes) oncogene ho	0,0165479	0,205389	1,54714
**Epha1**	Eph receptor A1	0,0087028	0,170474	1,54779
**Apol11b**	apolipoprotein L 11b	0,0151097	0,199098	1,54982
**Prm1**	protamine 1	0,0260503	0,233674	1,55051
**Hipk2**	homeodomain interacting protein kinase 2	0,0377939	0,261598	1,55131
**Wee1**	WEE 1 homolog 1 (S. pombe)	0,0351425	0,257842	1,55142
**Mep1b**	meprin 1 beta	0,0008053	0,0864886	1,55146
**Atl2**	atlastin GTPase 2	0,005331	0,14802	1,55553
**Zyx**	zyxin	0,0052447	0,14802	1,55564
**Vamp2**	vesicle-associated membrane protein 2	0,006666	0,156618	1,55604
**Srd5a3**	steroid 5 alpha-reductase 3	0,0242747	0,229728	1,55767
**Slc5a9**	solute carrier family 5 (sodium/glucose cotran	0,0004714	0,0849322	1,55882
**Gpr151**	G protein-coupled receptor 151	0,0404354	0,267051	1,56009
**2900006B11Rik**	RIKEN cDNA 2900006B11 gene	0,0073895	0,161438	1,56205
**---**	---	0,0264582	0,235012	1,56251

Table S1.

**Cpm**	carboxypeptidase M	0,013529	0,192879	1,56344
**Abcb1a**	ATP-binding cassette, sub-family B (MDR/TAP	0,0058443	0,150903	1,56457
**AW112010**	expressed sequence AW112010	0,0120153	0,186803	1,56547
**Vps37a**	vacuolar protein sorting 37A (yeast)	0,0084357	0,16957	1,56589
**Trim30d**	tripartite motif-containing 30D	0,0424954	0,270264	1,56615
**Gbp7**	guanylate binding protein 7	0,0475017	0,278552	1,56694
**Smpx**	small muscle protein, X-linked	0,0162906	0,204078	1,56705
**Gramd3**	GRAM domain containing 3	0,0049037	0,14496	1,56887
**Osbpl6**	oxysterol binding protein-like 6	0,0004612	0,0849322	1,56908
**Ttc39c**	tetratricopeptide repeat domain 39C	0,0011745	0,097786	1,5691
**Per3**	period homolog 3 (Drosophila)	0,0187062	0,213166	1,5705
**Evl**	Ena-vasodilator stimulated phosphoprotein	0,0366739	0,259502	1,57069
**Tmem117**	transmembrane protein 117	0,0142094	0,195178	1,57077
**Rgs2**	regulator of G-protein signaling 2	0,0001784	0,0831415	1,57156
**AI849538**	expressed sequence AI849538	0,0413233	0,268297	1,57246
**Rnd2**	Rho family GTPase 2	0,0122279	0,187704	1,57273
**Paqr7**	progestin and adipoQ receptor family membe	0,0317946	0,250162	1,57281
**Lrrtm1**	leucine rich repeat transmembrane neuronal	0,0071587	0,160198	1,57282
**Sh2d6**	SH2 domain containing 6	0,0169187	0,20732	1,57284
**Adh6a**	alcohol dehydrogenase 6A (class V)	0,0036719	0,132256	1,57309
**Nelf**	nasal embryonic LHRH factor	0,0393862	0,264919	1,57317
**Tsku**	tsukushi	0,0212625	0,220911	1,57421
**Dgcr6**	DiGeorge syndrome critical region gene 6	0,0033509	0,130776	1,57445
**Rabgap1l**	RAB GTPase activating protein 1-like	0,0126415	0,189766	1,57454
**Slc5a12**	solute carrier family 5 (sodium/glucose cotran	0,0198374	0,216477	1,57461
**Ankrd12**	ankyrin repeat domain 12	0,0098005	0,176669	1,57486
**Tmc7**	transmembrane channel-like gene family 7	0,0143257	0,195852	1,57536
**Zc3h15**	zinc finger CCCH-type containing 15	0,0153539	0,200277	1,57803
**Apol11b**	apolipoprotein L 11b	0,0035073	0,131563	1,57926
**Gramd3**	GRAM domain containing 3	0,0005361	0,0849322	1,57975
**Gas2l3**	growth arrest-specific 2 like 3	0,046066	0,276084	1,58039
**Gm15527**	Predicted gene 15527	0,0425171	0,270264	1,58064
**Abhd2**	abhydrolase domain containing 2	0,011691	0,185429	1,58083
**Vwce**	von Willebrand factor C and EGF domains	0,011613	0,1854	1,58098
**Ccbe1**	collagen and calcium binding EGF domains 1	0,0181826	0,211172	1,58128
**Gstm3**	glutathione S-transferase, mu 3	0,0356964	0,258379	1,58148
**Zfp40**	Zinc finger protein 40	0,0324174	0,251402	1,58289
**Il18**	interleukin 18	0,0171379	0,208575	1,58299
**Slc46a1**	solute carrier family 46, member 1	0,0359685	0,258387	1,58302
**Nlrc3**	NLR family, CARD domain containing 3	0,0280191	0,238017	1,58393
**Pxk**	PX domain containing serine/threonine kinase	0,0017868	0,109867	1,58557
**---**	---	0,0154487	0,200278	1,58661
**---**	---	0,0307826	0,246117	1,58698
**Gna13**	guanine nucleotide binding protein, alpha 13	0,0027401	0,124236	1,58723
**Ang3**	angiogenin, ribonuclease A family, member 3	0,0347993	0,257329	1,58732
**D9Ertd256e**	DNA segment, Chr 9, ERATO Doi 256, expresse	0,0146048	0,19754	1,58735
**Prnd**	prion protein dublet	0,0013595	0,103163	1,58934
**Plekhh1**	pleckstrin homology domain containing, famil	0,0014259	0,103881	1,58979
**Aqp1**	aquaporin 1	0,0308212	0,246215	1,5903
**Slc39a14**	solute carrier family 39 (zinc transporter), mei	0,0040653	0,13684	1,59057
**Nek6**	NIMA (never in mitosis gene a)-related expres	0,028001	0,238017	1,59114
**Myo7a**	myosin VIIA	0,0357903	0,258379	1,5922

Table S1.

---	---	0,0032828	0,13061	1,59278
**Entpd4 /// LOC100862375**	ectonucleoside triphosphate diphosphohydro	0,0150671	0,198997	1,59364
**Peli1**	pellino 1	0,0048711	0,14496	1,59381
**Eif2c4**	eukaryotic translation initiation factor 2C, 4	0,0155603	0,201067	1,59386
---	---	0,016048	0,20288	1,59391
**Trim5**	tripartite motif-containing 5	0,0495026	0,282053	1,59433
**Clec16a**	C-type lectin domain family 16, member A	0,0386342	0,263865	1,59456
---	---	0,0371117	0,260249	1,59518
**Lhpp**	phospholysine phosphohistidine inorganic pyr	0,0318965	0,250162	1,59846
**Ctso**	cathepsin O	0,0497566	0,282091	1,59855
**Mansc1**	MANSC domain containing 1	0,0007358	0,0849322	1,59969
**Stat1**	signal transducer and activator of transcriptio	0,0397355	0,265759	1,60164
**Entpd7**	ectonucleoside triphosphate diphosphohydro	0,0023062	0,119793	1,60172
**Acvr1c**	activin A receptor, type IC	0,0032261	0,129936	1,60214
**Reg3a**	regenerating islet-derived 3 alpha	0,0053135	0,14802	1,60264
**Apol7b /// Apol7e**	apolipoprotein L 7b /// apolipoprotein L 7e	0,0222829	0,224626	1,60317
**Hist2h2be**	histone cluster 2, H2be	0,0178037	0,209832	1,60372
**Zkscan1**	zinc finger with KRAB and SCAN domains 1	0,0027401	0,124236	1,60377
**2900042A17Rik**	RIKEN cDNA 2900042A17 gene	0,024543	0,229994	1,60442
**Asah1**	N-acylsphingosine amidohydrolase 1	0,0173823	0,20885	1,60665
**Gramd1b**	GRAM domain containing 1B	0,0306751	0,245889	1,60686
**Gm20004**	predicted gene, 20004	0,003594	0,131572	1,60719
**Ifih1**	interferon induced with helicase C domain 1	0,0365091	0,259258	1,60851
**1110037F02Rik**	RIKEN cDNA 1110037F02 gene	0,026981	0,235813	1,6089
**E230008J23Rik**	RIKEN cDNA E230008J23 gene	0,003302	0,13061	1,60907
**Arhgap17**	Rho GTPase activating protein 17	0,0470499	0,277368	1,6091
**Rhoq**	ras homolog gene family, member Q	0,0005642	0,0849322	1,6102
---	---	0,0054056	0,148248	1,6103
**Ccnd1**	cyclin D1	0,0038778	0,134489	1,61033
**Fasl**	Fas ligand (TNF superfamily, member 6)	0,0237567	0,228298	1,61068
**Aplp1**	amyloid beta (A4) precursor-like protein 1	0,0197905	0,216393	1,61163
**Ugcg**	UDP-glucose ceramide glucosyltransferase	0,0274608	0,236926	1,61241
**Slc20a1**	solute carrier family 20, member 1	0,0011833	0,0982265	1,61265
**Gramd1b**	GRAM domain containing 1B	5,52E-05	0,0726097	1,61462
**Hist1h3b /// Hist1h3c /// Hi**	histone cluster 1, H3b /// histone cluster 1, H3	0,0405894	0,267205	1,61593
**Slc5a6**	solute carrier family 5 (sodium-dependent vit	0,0119387	0,186618	1,61641
**AI043046**	expressed sequence AI043046	0,0005977	0,0849322	1,61673
**Zdhhc6**	zinc finger, DHHC domain containing 6	0,0391076	0,264405	1,61794
**Ssr1**	Signal sequence receptor, alpha	0,0088073	0,170577	1,6189
**Wipf3**	WAS/WASL interacting protein family, membe	0,0209366	0,219995	1,61975
**Pcdh9**	protocadherin 9	0,005952	0,152151	1,62008
**Ubc**	ubiquitin C	0,0010935	0,0951068	1,62085
**Abhd2**	abhydrolase domain containing 2	0,0473708	0,278295	1,62137
**Mettl7b**	methyltransferase like 7B	0,0332591	0,252993	1,62174
---	---	0,0256779	0,232501	1,62181
**Fgl2**	fibrinogen-like protein 2	0,0069272	0,159545	1,62219
**Bst1**	bone marrow stromal cell antigen 1	0,0283759	0,238692	1,62396
**Igsf3**	immunoglobulin superfamily, member 3	0,0102757	0,17912	1,62397
**Edem2**	ER degradation enhancer, mannosidase alpha	0,0296496	0,243156	1,62398
**Entpd4**	ectonucleoside triphosphate diphosphohydro	0,0083881	0,169445	1,62523
**Slc5a9**	solute carrier family 5 (sodium/glucose cotrar	0,0127845	0,189985	1,6255
**Abcc2**	ATP-binding cassette, sub-family C (CFTR/MRI	0,0189611	0,213614	1,62556

Table S1.

**Gm19434**	predicted gene, 19434	0,012011	0,186803	1,62666
**Entpd7**	ectonucleoside triphosphate diphosphohydro	0,002268	0,119793	1,62703
**Ppargc1a**	peroxisome proliferative activated receptor, g	0,0492115	0,281424	1,62799
**3110007F17Rik /// Gm2411**	RIKEN cDNA 3110007F17 gene /// predicted g	0,0362038	0,258771	1,62958
**Cyp3a25**	cytochrome P450, family 3, subfamily a, polyp	0,0001311	0,0805578	1,63102
**Hopx**	HOP homeobox	0,0136705	0,193089	1,63121
**Amn**	amnionless	0,0003689	0,0849322	1,63245
**---**	---	0,0008193	0,0864886	1,63276
**Xdh**	xanthine dehydrogenase	0,0373772	0,260842	1,63364
**---**	---	0,0261723	0,234062	1,63454
**---**	---	0,0128341	0,189985	1,63632
**Dusp23**	dual specificity phosphatase 23	0,0066037	0,156088	1,63633
**2010003K11Rik**	RIKEN cDNA 2010003K11 gene	0,0026516	0,123202	1,64042
**---**	---	0,0054123	0,148248	1,64267
**Gda**	guanine deaminase	0,0031389	0,128056	1,64287
**Prkg2**	protein kinase, cGMP-dependent, type II	0,0006038	0,0849322	1,64319
**Dnahc2**	dynein, axonemal, heavy chain 2	0,0014727	0,104066	1,64324
**Cphx**	Cytoplasmic polyadenylated homeobox	0,0368877	0,260059	1,64363
**Gm10393 /// Gm9780 /// Pl**	predicted gene 10393 /// predicted gene 978(	0,0093053	0,1739	1,64413
**Gda**	guanine deaminase	0,0441115	0,272416	1,64418
**---**	---	0,0088151	0,170577	1,64482
**Tap1**	transporter 1, ATP-binding cassette, sub-famil	0,0446291	0,273589	1,64493
**Abcg8**	ATP-binding cassette, sub-family G (WHITE), n	0,0096365	0,176067	1,64583
**Ccng2**	cyclin G2	0,0117645	0,185859	1,64596
**---**	---	0,0110962	0,183584	1,64604
**---**	---	0,0131233	0,190604	1,64626
**Ak7**	adenylate kinase 7	0,0008152	0,0864886	1,64708
**Pafah1b1**	platelet-activating factor acetylhydrolase, isof	0,0066214	0,156088	1,64722
**Homer2**	homer homolog 2 (Drosophila)	0,0252577	0,231452	1,64818
**Prkg2**	protein kinase, cGMP-dependent, type II	0,0087292	0,170474	1,65018
**Rdh5**	retinol dehydrogenase 5	0,0017271	0,109277	1,65041
**Leap2**	liver-expressed antimicrobial peptide 2	0,0267644	0,235425	1,65066
**Gpd2**	glycerol phosphate dehydrogenase 2, mitocho	0,0214663	0,22139	1,65197
**Adamtsl5**	ADAMTS-like 5	0,0002013	0,0831415	1,65356
**Cml2**	camello-like 2	0,035114	0,257732	1,6537
**LOC552908**	uncharacterized LOC552908	0,0097997	0,176669	1,65466
**Ept1**	ethanolaminephosphotransferase 1 (CDP-etha	0,0242941	0,229728	1,65489
**Wee1**	WEE 1 homolog 1 (S. pombe)	0,0088479	0,170716	1,65526
**Ace**	angiotensin I converting enzyme (peptidyl-dip	0,0101935	0,178809	1,6579
**Hemgn**	hemogen	0,0024595	0,120058	1,65801
**Mfsd2b**	Major facilitator superfamily domain containi	0,0108441	0,182345	1,66026
**Tcea3**	transcription elongation factor A (SII), 3	0,0028204	0,124716	1,66148
**Slc6a3**	solute carrier family 6 (neurotransmitter trans	0,0194551	0,215395	1,66242
**D14Ertd449e**	DNA segment, Chr 14, ERATO Doi 449, express	0,0059967	0,152151	1,66296
**Cmpk2**	cytidine monophosphate (UMP-CMP) kinase 2	0,0274443	0,236926	1,66306
**Hist1h1c**	histone cluster 1, H1c	0,0116278	0,1854	1,66554
**Tgtp1 /// Tgtp2**	T cell specific GTPase 1 /// T cell specific GTPa	0,0365852	0,259258	1,66597
**---**	---	0,0054424	0,148673	1,67003
**Phospho1 /// Zfp652**	phosphatase, orphan 1 /// zinc finger protein	0,0128413	0,189985	1,67237
**Usp2**	ubiquitin specific peptidase 2	0,0040434	0,136529	1,67242
**Pcm1**	pericentriolar material 1	0,0101306	0,178366	1,67304
**Gstk1**	glutathione S-transferase kappa 1	0,0213244	0,221191	1,67343

Table S1.

**Rgs2**	regulator of G-protein signaling 2	0,0173303	0,208838	1,67496
**Arglu1**	arginine and glutamate rich 1	0,0112927	0,18408	1,67542
**Gstm6**	glutathione S-transferase, mu 6	9,59E-05	0,0804523	1,67632
**Ccl6**	chemokine (C-C motif) ligand 6	0,0183973	0,212189	1,67746
**---**	---	0,0003587	0,0849322	1,67824
**Tgfbi**	transforming growth factor, beta induced	0,005602	0,149218	1,6805
**Cml5**	camello-like 5	0,0265821	0,23541	1,6815
**Gatm**	glycine amidinotransferase (L-arginine:glycine	0,0237417	0,228298	1,68194
**2610024D14Rik**	RIKEN cDNA 2610024D14 gene	0,004247	0,138661	1,68238
**Edaradd**	EDAR (ectodysplasin-A receptor)-associated d	0,0014977	0,104066	1,68266
**Clic6**	chloride intracellular channel 6	0,00299	0,125847	1,68402
**Igsf3**	immunoglobulin superfamily, member 3	0,0015757	0,105602	1,68434
**Efhd2**	EF hand domain containing 2	0,0096243	0,176067	1,68659
**Apoc3**	apolipoprotein C-III	7,33E-05	0,0726097	1,6866
**---**	---	0,0374403	0,260854	1,68671
**Per2**	period homolog 2 (Drosophila)	0,0416497	0,268297	1,68692
**---**	---	0,0019056	0,113932	1,6884
**Usp2**	ubiquitin specific peptidase 2	0,0095523	0,175509	1,69008
**Slc25a22**	solute carrier family 25 (mitochondrial carrier	0,0152442	0,199825	1,69093
**Ddx58**	DEAD (Asp-Glu-Ala-Asp) box polypeptide 58	0,040386	0,266941	1,69132
**D5Ertd579e**	DNA segment, Chr 5, ERATO Doi 579, expresse	0,0092667	0,173659	1,6944
**Ace**	angiotensin I converting enzyme (peptidyl-dip	0,003032	0,125899	1,69448
**Tmem56**	transmembrane protein 56	0,0125069	0,189441	1,69458
**1700019G17Rik**	RIKEN cDNA 1700019G17 gene	0,0005582	0,0849322	1,69519
**Trdmt1**	tRNA aspartic acid methyltransferase 1	0,0040846	0,136873	1,69553
**---**	---	0,0064741	0,155345	1,69554
**Mcoln2**	mucolipin 2	0,0004165	0,0849322	1,69612
**Sfrp5**	secreted frizzled-related sequence protein 5	0,0026759	0,123786	1,69628
**---**	---	0,0020493	0,116766	1,69643
**Ccr9**	chemokine (C-C motif) receptor 9	0,0233151	0,227279	1,69658
**---**	---	0,0253367	0,231521	1,69697
**Fam46a**	family with sequence similarity 46, member A	0,0008431	0,0864886	1,69742
**Aldh1a7**	aldehyde dehydrogenase family 1, subfamily A	0,0414434	0,268297	1,69955
**Gstm2**	glutathione S-transferase, mu 2	0,0006063	0,0849322	1,69992
**Fam132a**	family with sequence similarity 132, member .	0,0054777	0,148764	1,70134
**Dpp4**	dipeptidylpeptidase 4	0,0087965	0,170577	1,70197
**Gstm3**	glutathione S-transferase, mu 3	0,0459387	0,275912	1,70231
**Camk2n1**	calcium/calmodulin-dependent protein kinase	0,0001792	0,0831415	1,70395
**Phlpp2**	PH domain and leucine rich repeat protein ph	0,0078056	0,165767	1,704
**Fam132a**	family with sequence similarity 132, member .	0,0036144	0,131572	1,70447
**---**	---	0,0012512	0,0999478	1,70509
**Gm20021**	predicted gene, 20021	0,0086303	0,170303	1,70642
**Gstm1**	glutathione S-transferase, mu 1	0,0072441	0,160297	1,70687
**Rabgap1l**	RAB GTPase activating protein 1-like	0,0278772	0,237798	1,70929
**Acp6**	Acid phosphatase 6, lysophosphatidic	0,0232435	0,226903	1,7104
**Tff3**	trefoil factor 3, intestinal	0,032343	0,251402	1,71325
**Dpp4**	dipeptidylpeptidase 4	0,0005081	0,0849322	1,71521
**---**	---	0,0011032	0,0951068	1,71556
**H2-DMa**	histocompatibility 2, class II, locus DMa	0,023653	0,228298	1,71721
**Cyp4v3**	cytochrome P450, family 4, subfamily v, polyp	0,0155204	0,200631	1,72104
**Casp3**	caspase 3	0,0077697	0,165436	1,72172
**Tmem66**	transmembrane protein 66	0,0025347	0,121373	1,72261

Table S1.

**Ppargc1a**	peroxisome proliferative activated receptor, g	0,0019158	0,114127	1,72458
**Slc13a2**	solute carrier family 13 (sodium-dependent di	0,0025449	0,121505	1,72474
**Nek6**	NIMA (never in mitosis gene a)-related expres	0,0094189	0,174332	1,72599
**Fut4**	fucosyltransferase 4	0,0168569	0,207102	1,72627
**Insig1**	insulin induced gene 1	0,0006009	0,0849322	1,7269
**Iigp1**	interferon inducible GTPase 1	0,0256659	0,232457	1,73149
**Tmem229b**	transmembrane protein 229B	0,0196289	0,215831	1,73159
**Ltb4r2**	leukotriene B4 receptor 2	0,0204932	0,218142	1,7324
**Cml2**	camello-like 2	0,0386696	0,263865	1,73615
**---**	---	0,0148819	0,198415	1,73695
**Acy3**	aspartoacylase (aminoacylase) 3	0,0040807	0,136873	1,73713
**Tef**	thyrotroph embryonic factor	0,00132	0,102302	1,74029
**Slc37a4**	solute carrier family 37 (glucose-6-phosphate	0,0001938	0,0831415	1,74043
**Pstpip2**	proline-serine-threonine phosphatase-interac	0,0039125	0,13512	1,74143
**Ccl3**	chemokine (C-C motif) ligand 3	0,0213946	0,22133	1,74166
**---**	---	0,0015387	0,104363	1,74318
**Tmsb15b2 /// Tmsb15l**	thymosin beta 15b2 /// thymosin beta 15b lik	0,0020005	0,115014	1,74344
**9330112F22Rik**	RIKEN cDNA 9330112F22 gene	0,0009826	0,0885303	1,7438
**Ascl2**	achaete-scute complex homolog 2 (Drosophila	0,0365853	0,259258	1,74395
**---**	---	0,0110371	0,183576	1,745
**Tmem56**	transmembrane protein 56	0,001964	0,114711	1,74505
**---**	---	0,0006837	0,0849322	1,74977
**Spns2**	spinster homolog 2 (Drosophila)	0,0023149	0,119793	1,75068
**Prkg2**	protein kinase, cGMP-dependent, type II	0,0011963	0,0985316	1,75481
**Calm1 /// Calm2 /// Calm3**	calmodulin 1 /// calmodulin 2 /// calmodulin 3	0,0073197	0,161207	1,75512
**Tmem41b**	transmembrane protein 41B	0,0116211	0,1854	1,75778
**Gls**	glutaminase	0,0039272	0,135435	1,75975
**Edaradd**	EDAR (ectodysplasin-A receptor)-associated d	0,0176935	0,209737	1,76102
**Slc1a5**	solute carrier family 1 (neutral amino acid tra	8,91E-05	0,0787623	1,76428
**---**	---	0,0099971	0,17805	1,76462
**Ccbe1**	collagen and calcium binding EGF domains 1	0,0187852	0,213166	1,76626
**4930551O13Rik**	RIKEN cDNA 4930551O13 gene	0,0326841	0,251925	1,76744
**Rgs2**	regulator of G-protein signaling 2	0,0054385	0,148673	1,76768
**Tmem139**	transmembrane protein 139	0,0163854	0,204614	1,76891
**Eny2**	enhancer of yellow 2 homolog (Drosophila)	0,0054902	0,148764	1,76963
**Nlrp9b**	NLR family, pyrin domain containing 9B	0,009268	0,173659	1,77334
**Eny2**	enhancer of yellow 2 homolog (Drosophila)	0,0049934	0,14588	1,77817
**Cst6**	cystatin E/M	0,0078581	0,16602	1,77898
**Clec16a**	C-type lectin domain family 16, member A	0,0052898	0,14802	1,77968
**Abca1**	ATP-binding cassette, sub-family A (ABC1), me	0,0390094	0,264304	1,77992
**Maf**	avian musculoaponeurotic fibrosarcoma (v-ma	0,0056034	0,149218	1,78112
**Plek2**	pleckstrin 2	0,0125397	0,189441	1,78139
**Vegfa**	vascular endothelial growth factor A	0,0220204	0,223797	1,78188
**Psmb8**	proteasome (prosome, macropain) subunit, be	0,0191742	0,214649	1,78218
**9130017K11Rik**	RIKEN cDNA 9130017K11 gene	0,0006017	0,0849322	1,78232
**Rnf213**	ring finger protein 213	0,0353101	0,257842	1,78263
**Rab6b**	RAB6B, member RAS oncogene family	0,0211794	0,220911	1,78383
**Abhd6**	abhydrolase domain containing 6	0,0292404	0,241248	1,78678
**Negr1**	neuronal growth regulator 1	0,0457848	0,275912	1,78689
**Abcg5**	ATP-binding cassette, sub-family G (WHITE), m	5,97E-05	0,0726097	1,78767
**Greb1**	gene regulated by estrogen in breast cancer p	0,0092914	0,173841	1,78974
**3110007F17Rik /// Gm2411**	RIKEN cDNA 3110007F17 gene /// predicted g	0,0071861	0,160198	1,7937

Table S1.

**H2-Ab1**	histocompatibility 2, class II antigen A, beta 1	0,0225819	0,225647	1,7942
**Mcoln3**	mucolipin 3	0,0281427	0,238244	1,80128
**Slc7a2**	solute carrier family 7 (cationic amino acid tra	0,0003718	0,0849322	1,80431
**Trpa1**	transient receptor potential cation channel, su	0,0260039	0,233538	1,80633
**Tgfbi**	transforming growth factor, beta induced	0,0118473	0,186102	1,82066
**1700019G17Rik**	RIKEN cDNA 1700019G17 gene	0,0278164	0,237798	1,82134
**Hpgd**	Hydroxyprostaglandin dehydrogenase 15 (NAI	0,0008831	0,0864886	1,82308
**9130004J05Rik**	RIKEN cDNA 9130004J05 gene	0,0384845	0,263699	1,82323
**Tnfsf10**	tumor necrosis factor (ligand) superfamily, me	0,0052329	0,14802	1,82665
**Gls**	glutaminase	0,0013398	0,102769	1,82678
**Pstpip2**	proline-serine-threonine phosphatase-interac	0,0189875	0,213839	1,82727
**Sema5a**	sema domain, seven thrombospondin repeats	0,0006608	0,0849322	1,82805
**Iyd**	iodotyrosine deiodinase	0,0079957	0,167413	1,82849
**Ccdc116**	coiled-coil domain containing 116	0,0008713	0,0864886	1,83261
**Pstpip2**	proline-serine-threonine phosphatase-interac	0,0033006	0,13061	1,83672
**Slc9a2**	solute carrier family 9 (sodium/hydrogen exch	0,0173598	0,20885	1,83893
**Klrd1**	killer cell lectin-like receptor, subfamily D, me	0,0237168	0,228298	1,84074
**Iapp**	islet amyloid polypeptide	0,0028982	0,125052	1,84088
**Aff4**	AF4/FMR2 family, member 4	0,000447	0,0849322	1,84093
**Prkca**	protein kinase C, alpha	0,0461641	0,276216	1,84318
**Gtpbp2**	GTP binding protein 2	0,0140162	0,19432	1,84556
**---**	---	0,0392366	0,264748	1,8504
**Tef**	thyrotroph embryonic factor	0,0011715	0,097786	1,85149
**Ttc39c**	tetratricopeptide repeat domain 39C	0,0128262	0,189985	1,85331
**Gip**	gastric inhibitory polypeptide	0,0057567	0,150585	1,85915
**---**	---	0,0243625	0,229817	1,86248
**Ttc39c**	tetratricopeptide repeat domain 39C	0,0066209	0,156088	1,86295
**Rbm39**	RNA binding motif protein 39	0,0268803	0,235563	1,866
**Hpgd**	hydroxyprostaglandin dehydrogenase 15 (NAI	0,0007142	0,0849322	1,86827
**Hopx**	HOP homeobox	0,0103752	0,179738	1,86848
**Cyp2d22**	cytochrome P450, family 2, subfamily d, polyp	0,0003267	0,0849322	1,8699
**H2-DMb1 /// H2-DMb2**	histocompatibility 2, class II, locus Mb1 /// his	0,0459319	0,275912	1,87063
**Rec8**	REC8 homolog (yeast)	0,0097133	0,176669	1,87585
**Cyp3a11**	cytochrome P450, family 3, subfamily a, polyp	0,0178823	0,210167	1,88333
**Il15**	interleukin 15	0,0039345	0,135435	1,88712
**H2-Q5**	histocompatibility 2, Q region locus 5	0,0348246	0,257329	1,88777
**Dclk1**	doublecortin-like kinase 1	0,0348475	0,257329	1,88919
**Acsm5**	acyl-CoA synthetase medium-chain family me	0,002407	0,120058	1,89064
**Vamp3**	vesicle-associated membrane protein 3	0,003062	0,126444	1,89406
**---**	---	0,004659	0,144019	1,89622
**Gas2l3**	growth arrest-specific 2 like 3	0,027475	0,236926	1,89636
**Slc13a1**	solute carrier family 13 (sodium/sulfate symp	0,0465913	0,276696	1,89656
**B3galtl**	beta 1,3-galactosyltransferase-like	0,0004562	0,0849322	1,89873
**Clic5**	chloride intracellular channel 5	0,0056093	0,149218	1,89901
**Iapp**	islet amyloid polypeptide	0,0251349	0,231021	1,90254
**Lct**	lactase	7,64E-06	0,0457467	1,90704
**Per2**	period homolog 2 (Drosophila)	0,0352573	0,257842	1,91048
**Tnik**	TRAF2 and NCK interacting kinase	0,0193062	0,214959	1,9134
**Muc2**	mucin 2	0,0154325	0,200278	1,91451
**Esrrg**	estrogen-related receptor gamma	0,0400231	0,266274	1,9205
**Clec2e**	C-type lectin domain family 2, member e	0,0043845	0,140446	1,92539
**Tmem229b**	transmembrane protein 229B	0,0058998	0,151693	1,9292

Table S1.

**Esrrg**	estrogen-related receptor gamma	0,0028023	0,124286	1,93175
**Sema5a**	sema domain, seven thrombospondin repeats	0,0018373	0,112105	1,93248
**H2-Q6 /// LOC68395**	histocompatibility 2, Q region locus 6 /// histc	0,034179	0,255386	1,93672
**Pipox**	pipecolic acid oxidase	0,0014675	0,104066	1,93978
**Mosc1**	MOCO sulphurase C-terminal domain containi	0,032357	0,251402	1,94211
**Prss30**	protease, serine, 30	0,0059784	0,152151	1,94478
**Rbm39**	RNA binding motif protein 39	0,0452517	0,275232	1,94568
**Clic5**	chloride intracellular channel 5	0,0016919	0,108097	1,94616
**Casp3**	caspase 3	0,0029007	0,125052	1,9508
**Mep1a**	meprin 1 alpha	0,0004186	0,0849322	1,95439
**Hist1h1c**	histone cluster 1, H1c	0,0086479	0,170303	1,95947
**Nampt**	nicotinamide phosphoribosyltransferase	0,002183	0,117875	1,96157
**Ces2a**	carboxylesterase 2A	0,0262621	0,234224	1,96805
**Nampt**	nicotinamide phosphoribosyltransferase	0,0008297	0,0864886	1,9851
**Btnl2 /// LOC100862597**	butyrophilin-like 2 /// butyrophilin-like protei	0,0237356	0,228298	1,98544
**Afp**	alpha fetoprotein	0,0012301	0,0998479	1,98611
**Ambp**	alpha 1 microglobulin/bikunin	0,0004837	0,0849322	1,98631
**Gpr133**	G protein-coupled receptor 133	0,0250004	0,230775	1,98871
**Acsf2**	acyl-CoA synthetase family member 2	0,0132433	0,191394	1,99299
**Sema7a**	sema domain, immunoglobulin domain (Ig), ar	0,0106022	0,180973	1,99325
**Cyp2b10**	cytochrome P450, family 2, subfamily b, polyp	0,0127947	0,189985	1,99849
**5033423K11Rik**	RIKEN cDNA 5033423K11 gene	0,0257183	0,232579	2,00362
**6030439D06Rik**	RIKEN cDNA 6030439D06 gene	0,0284284	0,238843	2,00465
**Per3**	period homolog 3 (Drosophila)	0,0090863	0,17254	2,01167
**Mfsd4**	major facilitator superfamily domain containir	0,0248125	0,230738	2,01348
**---**	---	0,0405577	0,267148	2,02013
**Adora2b**	adenosine A2b receptor	0,0312873	0,247956	2,02117
**D14Ertd449e /// Gm10395 /**	DNA segment, Chr 14, ERATO Doi 449, express	0,0042002	0,138073	2,02477
**Fut4**	fucosyltransferase 4	0,0172171	0,208575	2,02497
**Bcmo1**	beta-carotene 15,15'-monooxygenase	0,0159218	0,202455	2,02757
**Cyp2b10**	cytochrome P450, family 2, subfamily b, polyp	0,0043553	0,139788	2,02998
**Cyp2b10**	cytochrome P450, family 2, subfamily b, polyp	0,006413	0,154787	2,03321
**H2-Aa**	histocompatibility 2, class II antigen A, alpha	0,0395493	0,26527	2,03653
**Npc1l1**	NPC1-like 1	0,000877	0,0864886	2,0367
**Tnfsf10**	tumor necrosis factor (ligand) superfamily, me	0,0172447	0,208575	2,03777
**Efr3b**	EFR3 homolog B (S. cerevisiae)	0,0099785	0,17805	2,04034
**Osbpl3**	oxysterol binding protein-like 3	0,0062809	0,154177	2,04875
**5033423K11Rik**	RIKEN cDNA 5033423K11 gene	0,0393756	0,264919	2,04972
**Cpm**	carboxypeptidase M	0,0158872	0,202412	2,05235
**Per3**	period homolog 3 (Drosophila)	0,0015294	0,104066	2,05312
**---**	---	0,0256461	0,232457	2,06675
**Mep1a**	meprin 1 alpha	0,0021679	0,117467	2,06831
**Nrg4**	neuregulin 4	0,0004347	0,0849322	2,07139
**Ccbe1**	collagen and calcium binding EGF domains 1	0,0385229	0,263796	2,07174
**---**	---	0,0024359	0,120058	2,07634
**Clic5**	chloride intracellular channel 5	0,0008216	0,0864886	2,07779
**Guca2b**	guanylate cyclase activator 2b (retina)	0,0005377	0,0849322	2,08022
**4430402I18Rik**	RIKEN cDNA 4430402I18 gene	0,000381	0,0849322	2,08837
**Ang4**	angiogenin, ribonuclease A family, member 4	0,0495495	0,282091	2,08977
**Nudt5**	nudix (nucleoside diphosphate linked moiety)	0,0087356	0,170474	2,09307
**Per3**	Period homolog 3 (Drosophila)	0,0022065	0,118676	2,11015
**4933440K10Rik**	RIKEN cDNA 4933440K10 gene	0,0224659	0,225314	2,13143

Table S1.

---	---	0,0082672	0,169163	2,13457
Mertk	c-mer proto-oncogene tyrosine kinase	0,0001324	0,0805578	2,14572
Tnfsf10	tumor necrosis factor (ligand) superfamily, me	0,0053497	0,14802	2,14983
Trdmt1	tRNA aspartic acid methyltransferase 1	0,0203116	0,217253	2,15704
Gstm1	glutathione S-transferase, mu 1	0,0003382	0,0849322	2,16056
---	---	0,0499189	0,282456	2,16176
Chn2	chimerin (chimaerin) 2	0,003765	0,13299	2,16251
---	---	0,006728	0,157284	2,16496
Adora2b	adenosine A2b receptor	0,0427949	0,270276	2,165
Lect2	leukocyte cell-derived chemotaxin 2	0,0008545	0,0864886	2,16539
Nudt5	nudix (nucleoside diphosphate linked moiety X	0,0085983	0,170243	2,16864
1300014I06Rik	RIKEN cDNA 1300014I06 gene	0,0394589	0,264936	2,17362
H2-T10 /// H2-T22 /// H2-T9	histocompatibility 2, T region locus 10 /// hist	0,0237269	0,228298	2,17531
Rgs13	regulator of G-protein signaling 13	0,0340557	0,255162	2,17647
Tgfbi	transforming growth factor, beta induced	5,02E-05	0,0726097	2,18519
Mtmr7	myotubularin related protein 7	0,0037452	0,13299	2,18688
Clic5	chloride intracellular channel 5	0,0002093	0,0834826	2,18869
A630038E17Rik	RIKEN cDNA A630038E17 gene	0,0224179	0,225067	2,19397
Igk-V1 /// Igkv9-120	immunoglobulin kappa chain variable 1 (V1) /,	0,0185286	0,21244	2,19426
Slc23a1	solute carrier family 23 (nucleobase transport	0,0130667	0,190354	2,1991
Dio1	deiodinase, iodothyronine, type I	0,0029967	0,125847	2,19945
Cd209a	CD209a antigen	0,002229	0,11892	2,20394
Tmc7	transmembrane channel-like gene family 7	0,012205	0,187704	2,20975
Tef	thyrotroph embryonic factor	0,0002423	0,0849322	2,21434
Rps4y2	ribosomal protein S4, Y-linked 2	0,0147849	0,198206	2,21439
H2-Q7 /// H2-Q8 /// H2-Q9 ,	histocompatibility 2, Q region locus 7 /// histo	0,027801	0,237798	2,21454
Gdap10	ganglioside-induced differentiation-associated	0,007967	0,167027	2,22134
Osbpl3	oxysterol binding protein-like 3	0,0001007	0,0805578	2,22173
Tgfbi	transforming growth factor, beta induced	2,51E-05	0,0726097	2,22486
Sema7a	sema domain, immunoglobulin domain (Ig), ar	0,0031155	0,127846	2,23346
Chn2	chimerin (chimaerin) 2	0,0002913	0,0849322	2,23427
---	---	0,0005402	0,0849322	2,24139
---	---	5,47E-05	0,0726097	2,25252
Hlf	hepatic leukemia factor	0,0001312	0,0805578	2,26266
Itln1 /// Itlnb /// LOC10086	intelectin 1 (galactofuranose binding) /// intel	0,0144754	0,19689	2,2634
Eepd1	endonuclease/exonuclease/phosphatase fami	0,0014896	0,104066	2,26363
Cym	chymosin	0,0272328	0,236497	2,26397
Iapp	islet amyloid polypeptide	2,92E-05	0,0726097	2,28549
Maf	avian musculoaponeurotic fibrosarcoma (v-ma	0,0092093	0,173397	2,3056
0610005C13Rik	RIKEN cDNA 0610005C13 gene	0,0029626	0,125632	2,32534
---	---	0,0407236	0,267335	2,3547
BC089597	cDNA sequence BC089597	0,0004277	0,0849322	2,35651
Acot12	acyl-CoA thioesterase 12	0,0131459	0,190828	2,36816
Acaa1b	acetyl-Coenzyme A acyltransferase 1B	0,0057165	0,150585	2,37169
Ces1g	carboxylesterase 1G	0,002259	0,119793	2,37979
---	---	0,001427	0,103881	2,38291
2600001M11Rik /// Nudt5	RIKEN cDNA 2600001M11 gene /// nudix (nuc	0,0013774	0,103511	2,4064
---	---	0,0005875	0,0849322	2,40643
Hsd17b13	hydroxysteroid (17-beta) dehydrogenase 13	0,0408106	0,267335	2,44013
Ear3	eosinophil-associated, ribonuclease A family,	0,0274391	0,236926	2,44333
Sema5a	sema domain, seven thrombospondin repeats	0,0137799	0,19383	2,45271
Slc44a4	solute carrier family 44, member 4	0,0160317	0,20288	2,46859

Table S1.

**Reg4**	regenerating islet-derived family, member 4	0,0158116	0,202351	2,46878
**Sst**	somatostatin	0,0027932	0,124286	2,51237
**Gstm1**	glutathione S-transferase, mu 1	0,0006017	0,0849322	2,54021
**Gdpd2**	glycerophosphodiester phosphodiesterase do	0,0003217	0,0849322	2,55215
**2310007B03Rik**	RIKEN cDNA 2310007B03 gene	0,0002055	0,0831415	2,57261
**Slc20a1**	solute carrier family 20, member 1	0,0004119	0,0849322	2,57518
**---**	---	0,0093912	0,174016	2,57546
**Gm10393 /// Gm9780 /// Pl**	predicted gene 10393 /// predicted gene 978(	0,0003255	0,0849322	2,60725
**Fras1**	Fraser syndrome 1 homolog (human)	0,000193	0,0831415	2,66639
**Gstm1**	glutathione S-transferase, mu 1	0,0003376	0,0849322	2,70216
**Ywhaz**	tyrosine 3-monooxygenase/tryptophan 5-mor	0,0007845	0,0864886	2,713
**Agmat**	agmatine ureohydrolase (agmatinase)	0,0002059	0,0831415	2,71734
**Bbox1**	butyrobetaine (gamma), 2-oxoglutarate dioxy	0,0009773	0,0882982	2,72906
**Ifit3**	interferon-induced protein with tetratricopep	0,0265777	0,23541	2,7371
**Per3**	period homolog 3 (Drosophila)	0,0017817	0,109867	2,75033
**Slc20a1**	solute carrier family 20, member 1	0,0003248	0,0849322	2,77176
**Per3**	period homolog 3 (Drosophila)	0,0002369	0,0849322	2,78098
**Bbox1**	butyrobetaine (gamma), 2-oxoglutarate dioxy	0,0180277	0,21057	2,78415
**Per2**	period homolog 2 (Drosophila)	0,0010179	0,0912052	2,81603
**Slc14a1**	solute carrier family 14 (urea transporter), me	0,0018747	0,113543	2,82264
**Pnliprp2**	pancreatic lipase-related protein 2	0,0109809	0,183113	2,85984
**Clps**	colipase, pancreatic	0,004201	0,138073	2,86067
**Gsdmc**	gasdermin C	0,011682	0,1854	2,8762
**S100g**	S100 calcium binding protein G	0,0008205	0,0864886	2,90015
**Otop3**	otopetrin 3	0,0009117	0,0869294	2,98421
**Dao**	D-amino acid oxidase	0,0001386	0,0824086	3,05239
**Hist1h2bc /// Hist1h2be ///**	histone cluster 1, H2bc /// histone cluster 1, H	0,0006852	0,0849322	3,07038
**Ugt2b5**	UDP glucuronosyltransferase 2 family, polype	0,0079144	0,166361	3,08691
**Dap**	death-associated protein	0,0066039	0,156088	3,09163
**Igtp**	interferon gamma induced GTPase	0,0281255	0,238244	3,09668
**---**	---	0,0023258	0,119793	3,10516
**Ccl24**	chemokine (C-C motif) ligand 24	0,0191407	0,214603	3,11524
**Mcoln3**	mucolipin 3	0,019548	0,215725	3,13056
**Clps**	colipase, pancreatic	0,0347464	0,257274	3,23402
**Cth**	cystathionase (cystathionine gamma-lyase)	2,23E-05	0,0726097	3,23901
**D530039A21Rik**	RIKEN cDNA D530039A21 gene	0,0113044	0,18408	3,28731
**Vps52**	vacuolar protein sorting 52 (yeast)	0,0319219	0,250241	3,32227
**Fbp1**	fructose bisphosphatase 1	0,0014429	0,104066	3,35717
**Nrn1**	neuritin 1	0,0071746	0,160198	3,3839
**Sphk2**	sphingosine kinase 2	0,0017442	0,109277	3,40709
**Dap**	death-associated protein	9,20E-05	0,0791844	3,50326
**Dbp**	D site albumin promoter binding protein	0,0006804	0,0849322	3,6216
**Slc36a1**	solute carrier family 36 (proton/amino acid sy	0,0037079	0,132893	3,67383
**Pldn**	pallidin	0,0116769	0,1854	3,77414
**Dbp**	D site albumin promoter binding protein	0,0007397	0,0849322	3,77502
**Usp18**	ubiquitin specific peptidase 18	0,0339439	0,255161	3,85699
**Afp**	alpha fetoprotein	0,0020782	0,116965	3,88046
**Susd2**	sushi domain containing 2	0,0085707	0,170163	3,97551
**Slc5a4b**	solute carrier family 5 (neutral amino acid trai	0,0044399	0,140705	3,98698
**Afp**	alpha fetoprotein	0,0001494	0,0831415	4,03546
**Huwe1**	HECT, UBA and WWE domain containing 1	0,0006023	0,0849322	4,1452
**Slc5a4a**	solute carrier family 5, member 4a	7,95E-06	0,0457467	4,22649

Table S1.

**Cubn**	cubilin (intrinsic factor-cobalamin receptor)	5,69E-05	0,0726097	4,36201
**C920025E04Rik /// H2-T23**	RIKEN cDNA C920025E04 gene /// histocompa	0,0232251	0,226903	4,41743
**Slc14a1**	solute carrier family 14 (urea transporter), me	0,0020934	0,116965	4,42131
**Mtmr7**	myotubularin related protein 7	0,0337883	0,254619	4,57238
**Cubn**	cubilin (intrinsic factor-cobalamin receptor)	0,0005952	0,0849322	4,71539
**Casp9**	caspase 9	4,25E-05	0,0726097	4,79596
**Vwa1**	von Willebrand factor A domain containing 1	0,0015015	0,104066	5,17822
**Gsdmc2 /// Gsdmc4**	gasdermin C2 /// gasdermin C4	0,0033438	0,130662	5,46347
**Ido1**	indoleamine 2,3-dioxygenase 1	0,0176474	0,209439	6,09459
**Hlf**	hepatic leukemia factor	0,0001023	0,0805578	7,63315
**H2-Eb1**	histocompatibility 2, class II antigen E beta	0,0426568	0,270264	9,44286
**Afp**	alpha fetoprotein	0,0001287	0,0805578	9,50652
**Slc23a1**	solute carrier family 23 (nucleobase transport	0,000338	0,0849322	9,63562
**Gsdmc2**	gasdermin C2	0,000932	0,0874427	10,0645

Table S2.

Gene name	Student's t-test q-value	Student's t-test Difference log2
Rce1	**0**	-3,561492284
Tut1	**0**	-3,305575689
Sh2d6	**0**	3,758471171
Spink4	**0**	5,105265935
Hsd17b7	**0**	2,569128036
Anxa13	**0**	-2,605108897
Gsdmc2	**0**	5,261334737
Rpl37	**0**	5,345128377
Cyp3a25;Cyp3a59	**0**	-7,113356908
Abcg5	**0**	-5,796192805
Tbc1d24	**0**	-3,113243103
Prdx6b	**0**	-2,987273534
Avil	**0**	4,290079117
Abcg8	**0**	-4,738356272
Abcc3	**0**	-1,911993027
Zdhhc5	**0**	2,384239833
Sh2d4a	**0**	2,708594004
Lrch4	**0**	4,249725342
Fbp1	**0**	-2,887975057
Stab1	**0**	2,895377477
Swi5	**0**	2,999487559
Mpp6	**0**	-2,880583445
Akr1b7	**0**	-2,065309525
2210407C18Rik	**0**	-2,335286458
Cftr	**0**	-3,430060705
Rps29	**0**	2,873217265
Gstm3	**0**	-3,109055837
Hsd17b2	**0**	-3,94479243
Tmem181a	**0**	-3,374627431
Ang4	**0**	4,230358124
Nup85	**0**	-4,27689298
Duoxa2	**0**	6,045036316
Prkci	**0**	-3,986339569
Tubb3	**0,003647059**	3,697418849
Tmem14c	**0,006842105**	-5,082531611
Rpl39	**0,007027027**	2,251173655
Grb7	**0,007222222**	5,183835983
Hmgcr	**0,007428571**	3,638182322
Rpp30	**0,0094**	3,905248006
Rabac1	**0,009641026**	4,076171239
Krt20	**0,010535211**	-1,219841003
Acot11	**0,010609929**	-1,181177775
Ggct	**0,010685714**	-2,647069931
Xdh	**0,01076259**	-1,944644928
Mkrn1	**0,01084058**	1,084830602
Irgm2	**0,010869565**	-2,418231328
Pnliprp2	**0,010919708**	3,369945526
Sdf4	**0,011**	-0,848723729
Psme1	**0,011081481**	-0,90858078
Krt79	**0,011111111**	4,863065084
Hacd2	**0,011164179**	1,668979009

Table S2.

Gyg;Gyg1	**0,01124812**	1,185599009
Acaa1b	**0,011333333**	-3,87232399
Myo7a	**0,011363636**	-3,533905665
Syn1	**0,011412587**	2,443011602
Ighv8-8	**0,011419847**	-3,535821915
Igf2r	**0,011457627**	-0,957439423
Selk	**0,011507692**	2,700767517
Ccdc6	**0,011522727**	-0,92812411
Serpinb2	**0,011588571**	3,2469546
Efnb2	**0,011596899**	-1,336685816
Rab43	**0,011627907**	-1,592569351
Cpn2	**0,011655172**	-3,556566874
Atg4b	**0,0116875**	-3,031484604
Sqle	**0,011706667**	3,245573044
Ido1	**0,011722543**	-4,336569468
Abhd6	**0,011744681**	-1,424830755
Adh6a	**0,011767442**	-1,785588582
Abcc2	**0,011779528**	-2,036511103
Tubb2a;Tubb2b	**0,011785235**	2,157501221
P4ha1	**0,011790698**	1,914907455
Rptor	**0,011825**	-2,158711116
Snrpd3	**0,011859649**	-0,932710012
Brox	**0,011864865**	-1,242764791
Cyp3a11	**0,011870968**	-1,623118718
Nxf1	**0,011873016**	3,729572296
Scin	**0,011899371**	1,465665817
Agmat	**0,011904762**	-1,608870188
Retnlb	**0,011905882**	2,610887527
S100g	**0,011929412**	-2,619778315
Chga	**0,011945578**	-1,592248281
Rps27l	**0,011955556**	2,935312271
Mttp	**0,011968**	-1,318284353
Me2	**0,011974684**	-1,050725301
Skiv2l2	**0,012**	-1,006560644
Ddc	**0,012**	-1,900332769
Tmprss15	**0,012022346**	-1,745124181
Plac8	**0,012027397**	-1,893887838
Huwe1	**0,012047619**	-1,070891698
Apol10a	**0,012050955**	-3,651602427
Chil4	**0,012064516**	3,631502151
Atpif1	**0,012071429**	-1,138995488
Ugt2b34	**0,012089888**	-1,6533343
Defa23	**0,012110345**	4,673302333
Pnpla6	**0,012128205**	-1,558227539
Foxred1	**0,012131868**	2,891393026
Acss1	**0,012143713**	1,300765355
Gpx2	**0,012162602**	2,747873306
Ugt2b38;Ugt2b5;Ugt2b17;Ugt2b37	**0,012192771**	-5,384475072
Rps27	**0,012194444**	3,971792857
Colgalt1	**0,012195122**	2,395249049
Wdr18	**0,012206452**	-1,057434082
Tmem51	**0,012216867**	-2,16950798

Table S2.

Duox2	**0,012261682**	3,664944967
Rpia	**0,012262295**	-1,442696889
Pon1	**0,012266667**	2,447975159
Uqcrh	**0,012285714**	1,480026245
Cyp2d22	**0,012290909**	-1,778741837
Tmed3	**0,012315789**	1,457261403
Map2k4	**0,012341463**	2,002801895
Krt32;Krt36;Krt33b;Krt31;Krt35	**0,012363636**	-4,286383947
Rbks	**0,012365854**	-1,126300176
Rps26	**0,012366013**	1,061855316
Gmfg	**0,012377358**	2,518304825
Abcb6	**0,012404494**	-3,379390717
Ctse	**0,012424779**	2,956005732
Elac2	**0,012441718**	-1,009771983
Cfap20	**0,012447368**	2,787312826
Fkbp4	**0,012466667**	-0,767496109
Phykpl	**0,012493827**	1,599524816
Nop9	**0,012495238**	-3,652545293
Gsn	**0,012518519**	1,120968501
Smpdl3b	**0,012529801**	-1,686282476
Fastkd2	**0,012535714**	-0,999604543
Baiap2l1	**0,012545455**	-1,11504364
Capn13	**0,012571429**	4,372180939
Rps15	**0,012583333**	1,970491409
Sarg	**0,012596273**	1,181954702
Agpat1	**0,012615385**	1,48637708
Mov10	**0,012648649**	-1,28861618
Lct	**0,01265**	-8,739983241
Clca1	**0,012677966**	1,656977336
Lsm5	**0,012689655**	-2,771149953
Serp1;Serp2	**0,012715789**	4,714055379
Rdh9	**0,012737864**	-3,059501012
Hn1	**0,012763636**	2,202843984
Prkca	**0,012786325**	-1,092013041
Smyd5	**0,012810127**	-1,319195429
Ndufb11	**0,012862745**	1,024429321
Atxn2	**0,012880734**	4,011669159
Brk1	**0,012896552**	-0,595505397
Pla2g4c	**0,012974359**	7,435346603
Gpr128	**0,012990099**	-2,014383952
Papss2	**0,013**	-1,246315002
Cluh	**0,013008696**	2,372016271
Adprhl2	**0,013106383**	4,112414678
Tex264	**0,01312**	3,577977498
Gys1	**0,013142857**	2,389410655
Cyp2b10	**0,013192982**	-3,145587921
Mocs1	**0,01319337**	-1,248012543
Entpd5	**0,013252525**	-1,555236181
Serpinb5	**0,013315789**	1,735587438
Ugdh	**0,013387755**	-1,633443197
Ces1f	**0,013428571**	-6,635749817
Dld	**0,013493333**	0,747339884

Table S2.

Alox5	**0,013525773**	3,543375015
Serf2;Serf1	**0,013672727**	2,210029602
S100a8	**0,013675676**	4,258649826
Micu1	**0,013692308**	-1,277879079
Tjap1	**0,013863014**	-1,001898448
Mtnd2	**0,013925926**	4,348999659
Ndrg2	**0,014055556**	1,690256119
Rnf181	**0,014188679**	-0,997849782
Dclk1	**0,014253521**	2,37528038
Efnb1	**0,014295082**	-1,484781265
Vdr	**0,014457143**	-2,021091461
Fth1	**0,014461538**	-2,648777008
Il18	**0,014468085**	-1,452742259
Slc4a7	**0,014545455**	-0,988777161
Abr	**0,014623656**	-1,194278717
Synrg	**0,014666667**	-1,153437932
Dynll1;BC048507	**0,014702703**	1,697318395
Defa-rs1;Gm14851	**0,014745098**	-2,586245219
B4galnt1	**0,014782609**	-1,257627487
Lgals3bp	**0,014882353**	-1,318120321
Maoa	**0,01504**	-1,756144842
Mlkl	**0,015050847**	-2,075286865
Nmes1;AA467197	**0,015104478**	2,013729731
Ugt1a1	**0,015310345**	-3,938533147
Synpo	**0,015333333**	3,633236567
Ces1	**0,015346939**	-3,87300237
Gstk1	**0,015365079**	-1,66036733
Gm8909	**0,015569231**	-3,428406398
Dnajb2	**0,015666667**	2,325461706
Gsdmc3	**0,0158125**	4,599916458
Sod1	**0,016**	-1,126162211
Otop3	**0,016063492**	-3,814849854
Lhpp	**0,016322581**	-2,440311432
AU040320;Kiaa0319l	**0,016460733**	-1,165500005
Slc39a14	**0,016590164**	-1,748009364
Psme2	**0,016695652**	-1,127153397
Mep1b	**0,016704846**	-2,182977676
Rfc3	**0,016776699**	-0,954274495
Ear1	**0,016778761**	4,545760473
Slc6a6	**0,016807512**	-0,932935715
Trrap	**0,016853333**	-1,769045512
Mgam	**0,016858537**	-1,953552246
Hmgcs1	**0,016866667**	1,427293142
Rpl28	**0,016886792**	1,643463135
Mical1	**0,016928571**	-1,029553731
Pnkd	**0,016929293**	1,870993296
Frg1	**0,016941176**	2,893075307
Uba6	**0,016966825**	-0,766638438
Gimd1	**0,017004484**	-2,249171575
Pdxdc1	**0,017015228**	-0,566835403
Ercc2	**0,017024631**	-1,575855255
Epha2	**0,017047619**	-1,663699468

Table S2.

Slc27a4	**0,017081081**	-0,998261134
Ca2;Car2	**0,017102041**	1,197886149
Plin3	**0,017108911**	-0,998992284
Trim31	**0,017129187**	-1,080083211
Lipt2	**0,017158371**	1,889917374
Soat1	**0,017189744**	0,975967407
Rpl18a	**0,01719403**	2,64995575
Hip1r	**0,017211538**	-0,47799174
Snx5	**0,01722807**	-0,623763402
Cyp2c55	**0,017236364**	-4,454121272
Nmral1	**0,017278351**	-1,02027003
Cbr1	**0,01728**	-1,49989446
Hmgn5	**0,017315068**	-0,991392136
Fhit	**0,017366834**	-3,168016434
Casp6	**0,017367876**	-1,036276499
Dusp3	**0,017394495**	2,075457255
Golm1	**0,017458333**	-0,919048309
Fam83h	**0,017474654**	3,195088069
Ces2b	**0,017555556**	-4,947239558
Cyp4f13	**0,017637209**	-3,455207825
Slc9a2	**0,017652893**	-1,572326024
Aaas	**0,017719626**	-2,597078959
H2-Ab1	**0,017726141**	-1,406604767
Xylb	**0,0178**	-1,732720057
Aoc1	**0,017874477**	-1,265060425
Sprr2a;Sprr2a3	**0,01794958**	4,741847992
Casp1	**0,018025316**	-1,080952962
Itfg3	**0,018101695**	-1,344264348
Lgals6	**0,018178723**	-6,191833496
Cyp4f40	**0,018200873**	-1,851554871
Nfu1	**0,01825641**	2,985860825
Bcl2l14	**0,018334764**	-0,84502538
Fkbp15	**0,018413793**	-1,312388102
Pdzk1	**0,018493506**	-2,791954676
Mrpl20	**0,01857377**	-1,407372157
Tep1	**0,018573913**	-1,190870285
Ptp4a2	**0,018650206**	-0,891342799
Erlin1	**0,01883004**	-2,337661107
Hint2	**0,018894309**	1,480604808
Vwf	**0,018904762**	-1,136359533
Las1l	**0,018971429**	-2,553238551
As3mt	**0,01898008**	-1,024184545
Slc30a10	**0,019056**	-1,246907552
Irak4	**0,019067669**	-1,310754776
Nelfb	**0,01913253**	-1,061116536
Cirbp	**0,019139623**	2,795636495
Gsta4	**0,019209677**	-2,334692637
Pds5b	**0,019212121**	-0,615970612
Gpd1	**0,019285171**	-1,309500376
Aif1	**0,019287449**	-1,051569621
Rpl24	**0,019328125**	1,247048696
Vipr1	**0,019358779**	-2,454165141

Table S2.

Prdx6	**0,019403922**	-0,653203328
Nr3c1	**0,01943295**	-1,128904978
Zfyve1	**0,019480315**	-1,470607758
Kiaa1467	**0,019507692**	-1,794165929
Tecr	**0,019583012**	0,534184774
Arhgap26	**0,01962963**	-1,252799352
Rpl27a	**0,019658915**	1,28395017
Sh2d7	**0,019702602**	4,05430603
Cs;Csl	**0,019735409**	0,905344645
Sh3bp1	**0,019776119**	-1,117568334
Sdc1	**0,019850187**	-1,176562627
Fdft1	**0,019896679**	2,245715459
L3mbtl1	**0,021**	-2,91109848
Pdlim2	**0,021421245**	-1,835542043
Rab1A	**0,021503546**	2,857599894
Coq6	**0,021580071**	-0,554960251
Itga9	**0,021605178**	-1,991235733
Wdr44	**0,021657143**	1,312557856
Gm28046;Rdh1;Rdh19	**0,021672727**	-2,405990601
Ncapg	**0,021675325**	-0,93806076
Ubl3	**0,021734767**	-1,244356791
Map3k2	**0,021745928**	-1,901062012
Hadh	**0,021751825**	-0,979709625
Stag2	**0,0218**	-0,786968231
Akp3	**0,02181295**	-1,83868281
Pld1	**0,021816993**	-0,937466304
Swap70	**0,02182716**	-0,883930206
Micu2	**0,021830986**	-1,033436457
Crot	**0,02187291**	-1,141318003
Enpp4	**0,021888525**	4,744160334
Cpq	**0,021891697**	-1,242900848
Rpl29;Gm17669;Gm10709;Gm3550	**0,021894737**	1,601357142
Msrb3	**0,021908127**	1,488018036
Me1	**0,021946309**	1,403251648
Adap1	**0,021960526**	-0,981724421
Dpm3	**0,021962733**	3,85180219
H2-Aa	**0,021971014**	-1,777825038
Snx4	**0,022006116**	-1,09903717
Chgb	**0,022020202**	-1,652362823
Pcsk5	**0,022031153**	-2,311508814
Ear6	**0,022033003**	1,575095495
Tap2	**0,022041958**	-0,957591375
Htatip2	**0,022057143**	-1,190601349
Tmem258	**0,02207362**	2,564788183
Arhgap17	**0,022094595**	-1,078070323
Cog5	**0,0221**	-0,439172109
Tomm70a	**0,02210596**	-0,81281662
Usp10	**0,022119298**	-1,061969121
Kiaa0196	**0,022127389**	-1,033221563
Fip1l1	**0,022129032**	4,507869085
Ankh	**0,022141538**	-3,387449265
Sympk	**0,022169279**	-1,016576767

Table S2.

Ear2	**0,022169492**	4,288029353
Kctd12	**0,022179402**	1,897732417
Aadac	**0,022198083**	-1,696587245
Ewsr1	**0,022238994**	0,967274348
Tmsb10	**0,022244898**	2,752635956
Pbld2	**0,022269231**	-1,389726639
Ppid	**0,022309148**	-0,810413361
Ldhb	**0,022317073**	1,190830866
Vps53	**0,022320819**	-1,693659465
Sfn	**0,022340836**	-0,793066661
St3gal4	**0,022355401**	-0,930872599
Larp4b	**0,022379747**	3,507967631
Txnrd2	**0,02239726**	-1,246374766
Otc	**0,022474227**	-1,112293879
Mia3	**0,022551724**	-1,246325175
Treh	**0,022629758**	-1,9245224
Ablim1	**0,022662614**	2,135648092
Apoc2	**0,022708333**	-1,906639099
Nup210	**0,023975758**	-0,738474528
Snf8	**0,024036036**	-0,898511251
Gm14409	**0,024108434**	-1,578564326
Ptgis	**0,024181269**	5,148537318
Adipoq	**0,024935065**	-0,916569392
Eea1	**0,024973913**	-0,623357773
Rnf114	**0,025**	0,794337591
Gstm6	**0,025002985**	-3,829331716
Snx2	**0,025046512**	-0,758053462
Cuta	**0,025065274**	-0,882174174
Atp7a	**0,025077844**	-1,092701594
Ccdc124	**0,025095101**	1,188521703
Gm6086;Gm9994;Gal3st2	**0,025119534**	-4,114041011
Cst3	**0,02513089**	3,375139872
Tm4sf5	**0,025147929**	-1,671864192
Rabl3	**0,02516763**	-0,813690821
Cggbp1	**0,025170213**	1,451763153
Ankmy2	**0,025173184**	-1,12467893
Bzw2	**0,025192982**	-0,895811081
Usp9x	**0,02519685**	2,811337789
Tns3	**0,025210811**	0,934647878
Fen1	**0,025222552**	-0,603568395
Tmsb15l;Tmsb15b1;Tmsb15b2	**0,025237333**	-1,282214483
Gusb	**0,025243697**	-1,133391062
2010107G23Rik	**0,025263158**	-1,507961273
Tom1l2	**0,025266862**	3,037590027
Elmo1	**0,025269122**	-0,687072754
Slc44a4	**0,025279133**	-1,350074768
Cct6b	**0,025297619**	-0,923368454
Ddhd2	**0,025304813**	-1,515966415
Syne2	**0,02530659**	-0,939463298
Slc33a1	**0,025307479**	-0,902560552
Hspb1	**0,025314607**	1,95467631
Galnt4	**0,025329815**	-0,668472926

Table S2.

U2af1	**0,025340659**	1,287786484
Nub1	**0,025340909**	-0,793923696
Wdr5	**0,025341176**	-0,873895009
Stat6	**0,025347826**	-1,068949382
Pafah1b3	**0,025372654**	-0,946840922
Itih4	**0,025377778**	-0,956794103
Lig1	**0,02537931**	-1,04782486
Rprd1b	**0,025385915**	-0,739318212
Ly75	**0,025396825**	-1,303162893
Clock	**0,025410468**	-1,066561381
Larp7	**0,025413105**	-0,966107686
Tmem120a	**0,025415929**	-1,27632459
Chchd5	**0,025416894**	-2,889750799
Pdpn	**0,02544086**	-2,029337565
Myof	**0,025448468**	0,618940353
Pnn	**0,025457627**	-0,602099737
Rpl3l	**0,02546114**	1,453545888
Acat1	**0,025464191**	0,888262431
Rab4a	**0,025480663**	-0,955922445
Pstpip2	**0,025485714**	-0,821707408
Cyp2d26	**0,025486339**	-1,624536514
Ppm1g	**0,025509434**	-0,532764435
Kpna4	**0,025556164**	-0,830005646
Htt	**0,025649485**	-0,836494446
Ppa1	**0,025715762**	-0,907272339
Tbcd	**0,025902314**	-0,759945552
Clic5	**0,02605102**	-1,336146673
Dram2	**0,026117647**	2,036824544
Fuca1	**0,026184615**	-1,290784836
Arpc5l	**0,02626972**	-1,118665695
Rpl14	**0,02648731**	1,531932195
Hsd17b11	**0,026756892**	-1,119944255
Sptb	**0,026762376**	-2,831659953
Tmem126b	**0,026824121**	-1,579915365
Agrn	**0,026828784**	-0,694877625
Nt5e	**0,026891688**	-0,777801514
Nvl	**0,026895522**	1,025319417
Fry	**0,026959596**	-0,9130675
Aldh18a1	**0,026962594**	-0,975054423
Hspb6	**0,026992665**	4,935557683
Actg1	**0,027027848**	5,218947093
Krtcap2	**0,02703**	2,742132187
Rnaset2	**0,027058824**	-1,298467
Pepd	**0,027125307**	-1,237759272
Sec14l2	**0,02715122**	-1,192654292
Mcm5	**0,027192118**	-0,868244807
H2-T23	**0,027259259**	-2,324245453
Inpp5b	**0,027271845**	-0,759786606
Gstm4	**0,0273382**	-1,368157705
Srrm1	**0,027864407**	-1,024168015
Slc35a3	**0,028**	1,55444018
Lyz2	**0,028064665**	3,661554337

Table S2.

Nanp	**0,028073394**	-1,717502594
Mrps23	**0,02812963**	-0,817617416
Hsph1	**0,028137931**	-0,703200022
Gm10094;Sap18	**0,028194896**	1,316384633
Ces1e	**0,028260465**	-1,657039642
Sis	**0,028320366**	-1,518795013
Idh3a	**0,02832634**	0,660054525
Calm1;Calml3	**0,028348235**	-0,913917542
Trim16	**0,028392523**	-1,250802994
Ccdc90b	**0,028415094**	-1,176068624
Wdr91	**0,028433735**	1,878442128
Apoa2	**0,028436364**	-0,973324458
Esyt1	**0,028459016**	0,772343953
Eln	**0,02848227**	-0,898040136
Slc30a5	**0,028501139**	-0,898660024
Abcb1a;Abcb1b	**0,028502415**	-1,41579628
Kiaa0020	**0,028523702**	-1,473737081
Dak	**0,028525822**	-0,971940358
Mthfd1l	**0,028526316**	-0,921475093
Tdrd7	**0,028549763**	-1,261979421
Tab1	**0,02856621**	-1,218947728
Ptprf	**0,028588235**	-2,240425746
Mki67	**0,028594724**	3,788967768
Bche	**0,028617577**	-2,413056691
Btaf1	**0,028653061**	-0,90567716
Cyp51a1	**0,028663462**	2,664209366
Rbm3	**0,028685714**	6,086173375
Rhpn2	**0,028711712**	-0,982477824
Coa3	**0,028732143**	1,805744807
Psmg3	**0,028754177**	-3,377598445
Znf768	**0,028796421**	1,412010829
Caprin1	**0,028860987**	-0,490481695
Pls1	**0,028925843**	-1,017208099
Tm4sf20	**0,0296**	-1,765931447
H2-T3	**0,029665924**	-2,215529124
Ppp1r1b	**0,029791574**	1,754562378
Srsf2	**0,030740088**	0,808054606
Commd6	**0,030807947**	1,78756841
Trpm4	**0,030876106**	-1,451647441
Msmo1	**0,030945055**	2,313344955
Plekho2	**0,031299781**	-0,782022476
Cth	**0,031368421**	-1,280345917
Rnf123	**0,031389978**	1,956497192
Nlrc4	**0,031446541**	-1,6463871
Stmn1	**0,031458515**	-0,906469345
Timm9	**0,0315**	-1,006853104
Pfdn2	**0,031512605**	-0,633898417
Palld	**0,031516949**	2,618036906
Arhgap27	**0,031547826**	-1,248179754
Snx7	**0,031568035**	-1,134731293
Clpb	**0,031578947**	-0,602220535
Ndufb4	**0,031583864**	1,237918218

Table S2.

Pdhb	**0,031636364**	1,092151006
Ahcyl2	**0,03164557**	-0,57027626
Arhgdib	**0,031651064**	1,461378098
Slc16a1	**0,031656652**	0,789677302
Gbp1	**0,031704989**	2,170850754
Lpp	**0,031712474**	1,40132014
Rps6ka1	**0,03171855**	-0,539976756
Hmgcl	**0,031724731**	-0,790173848
Aldh1a1	**0,031786325**	-1,405832926
Msh2	**0,031832636**	-0,73976771
Clrn3	**0,03185439**	0,745613734
Bzw1	**0,032267223**	-0,72905604
Lpcat3	**0,032390852**	-1,075717926
Sarnp	**0,032458333**	-0,438947042
Dus3l	**0,032514286**	1,84869194
Rpe	**0,032539419**	-0,764565786
Echs1	**0,032557769**	0,979631424
Cluh	**0,032560976**	-0,625062307
Atg16l1	**0,032580777**	-0,883625031
Zfand2b	**0,032622754**	1,997732798
Rgs10	**0,032627291**	-0,807399114
Krt13	**0,032647541**	-2,457819621
Fhl2	**0,032688**	3,294181188
Ap4s1	**0,032714579**	-1,150419235
Clic6	**0,032753507**	-0,946733475
Rpl21	**0,032763419**	1,340487798
Smdt1	**0,032781893**	2,624276479
Ybx3	**0,032819277**	1,580254237
Atp5j	**0,032849485**	-0,918667475
Pkp1	**0,032874494**	-1,264663061
Col12a1	**0,032880952**	-0,679506938
H2afx	**0,032885312**	1,595064163
Nbeal1	**0,032917355**	-1,722054164
Srsf9	**0,032941176**	1,152037938
Strbp	**0,032951613**	-0,978863398
Cfi	**0,032985507**	-0,853706996
Arhgap18	**0,033018182**	-0,816678365
Pus1	**0,033449505**	-0,708231608
Man2b1	**0,033897233**	-0,776280085
Sult1b1	**0,034074951**	-1,015797933
Lipe	**0,034251969**	-1,611990611
Ceacam1	**0,034658869**	-0,924058914
Mapk9	**0,034682353**	-0,929154078
Calml4	**0,034726563**	-1,019316355
Cap2	**0,034750491**	0,798754374
Rpl31	**0,034794521**	1,859345118
Txnrd1	**0,034930769**	-1,037974675
Eif1ad	**0,034937984**	1,546024323
Suclg1	**0,034998073**	0,865495682
Slc25a12	**0,035005825**	0,625774384
Mtch1	**0,035065637**	0,820450465
Dhrs11	**0,03507393**	-1,187800725

Table S2.

Alpi	**0,035133462**	-1,281256358
Stard4	**0,035586207**	1,176367442
Apoa4	**0,035654511**	-1,053254445
Ifi47	**0,035690566**	-0,998904546
Cyp2c66	**0,035758034**	-1,856433868
Ntmt1	**0,035783178**	-2,27613767
Csnk2b	**0,035825758**	1,164508184
Ppcs	**0,035850187**	-0,779385885
Nup133	**0,035879473**	3,285128276
Col4a3bp	**0,035880597**	-1,147264481
Snx1	**0,035885496**	-0,605102539
Pmm1	**0,035893738**	2,805149078
Hectd1	**0,035917448**	0,810800552
F13a1	**0,03593321**	4,59022522
Fuca2	**0,035954111**	-0,87741979
Rdh7	**0,035961977**	-1,943016052
Hook1	**0,035984962**	-0,439746857
Lgals2	**0,036**	-0,88218689
Cdcp1	**0,03601444**	-1,27272288
Tmco1	**0,036030476**	1,306588491
Rps9	**0,036067039**	1,459582647
Rpl18	**0,036079566**	1,968537649
Psmd8	**0,036107914**	-0,393330892
Mrps35	**0,036144928**	-0,705278397
Phb	**0,036172973**	1,032182693
Rab24	**0,036200717**	1,552565893
Ttr	**0,036210526**	-1,317406336
Fam91a1	**0,036251852**	-0,748435338
Fn1	**0,036265709**	1,57855161
Hccs	**0,036276364**	-1,085205714
Ugt2a3	**0,036342441**	-1,635721207
Tpr	**0,036408759**	-0,409330368
Tubb6	**0,03647532**	1,76622963
Commd7	**0,036493554**	-2,959668477
Ahcyl1	**0,036522361**	-1,136145274
Rpl26	**0,036542125**	0,838667552
Stk3	**0,036560886**	-1,355829239
Ifit1	**0,036609174**	-1,179703395
Cast	**0,036614286**	1,734460831
Nmnat3	**0,036628466**	-3,054054896
Crk	**0,036676471**	1,487795512
Samhd1	**0,036862191**	-0,644424438
Dpp4	**0,036927434**	-1,218521754
Enpep	**0,036992908**	-1,05840683
Dsp	**0,037058615**	-0,451749166
Lamtor1	**0,037124555**	0,535449346
Krt18	**0,037178131**	0,685305278
Akr1b8	**0,037190731**	-1,115992864
Rock2	**0,037209139**	-0,640602748
Dera	**0,037274648**	-0,515831629
Rpl6;Gm5428	**0,037405772**	1,634690603
Epb41l3	**0,037469388**	0,887992223

Table S2.

Eepd1	**0,03753322**	-2,080243429
Nif3l1	**0,03759727**	-0,691724141
Tsc22d1	**0,037661538**	-1,506918589
Cisd1	**0,037693333**	1,255963643
Supt16;Supt16h	**0,037698816**	-0,516705195
Commd8	**0,037726027**	-0,531784058
Gprc5a	**0,037731343**	1,86158371
Gclc	**0,03775626**	-1,005069097
Mrps15	**0,037762712**	-0,998849869
Sumo1	**0,037790738**	-0,706454595
Nhp2l1	**0,03779402**	1,392817179
Dis3l2	**0,037819398**	-0,901740392
Ptk7	**0,037844595**	-1,664564133
Mrrf	**0,03785567**	-0,670604706
Vdac1	**0,037856905**	1,021642685
Pip4k2a	**0,037874172**	1,527876536
Cct5	**0,037882747**	-0,390085856
Nostrin	**0,037920826**	-0,928986867
Ces2a	**0,03794087**	-1,654364904
Triap1	**0,037946309**	-1,08973376
B4galnt2	**0,037986207**	-1,018287023
Hsd17b6	**0,038006969**	-3,116512299
Ace	**0,038010084**	-1,650245667
Oxct1	**0,038016529**	0,931944529
Myo1f	**0,038051813**	1,967356364
Gng5	**0,038073298**	-1,069854101
Rpl13	**0,038074074**	1,37818718
Ctsh	**0,038117647**	-0,89840126
Mettl7b	**0,03813828**	-1,469804764
Rps2;Gm5786;Gm8225	**0,03813986**	0,851568858
Mapk10;Mapk8	**0,038158416**	-1,431585312
Wipf1	**0,038183709**	1,842782338
Rpl27	**0,038206655**	2,148014704
Usmg5	**0,03825**	2,292674383
Smc1a	**0,038273684**	-0,479103088
Stat1	**0,038728171**	-0,82054774
Pdha1	**0,038784893**	1,247526805
Dynll2	**0,038848684**	1,458118439
Atp5i	**0,038915584**	2,368718465
Myo9b	**0,038932039**	-0,966662089
Nup107	**0,038944262**	-0,774672826
Anxa1	**0,038978862**	0,737284342
Tm4sf4	**0,038995138**	-1,550079981
Dnajb12	**0,039019608**	-0,861607234
Smarcc2	**0,039042345**	-0,576186498
Sep-02	**0,039069467**	0,717487335
Pvr	**0,03908347**	-1,239006678
Dnaaf5	**0,039106036**	-0,734517415
Muc13	**0,039466238**	-0,96462059
Guk1	**0,039529791**	-0,501396815
Dpy30	**0,039593548**	-0,797250748
Nudt5	**0,039820225**	-1,046974182

Table S2.

Vars2	**0,0403776**	-0,691696803
Anxa11	**0,040442308**	-0,652862549
Psmd1	**0,040498403**	-0,517396927
Rpl36a	**0,04066563**	1,749471029
Cct6a	**0,040728972**	-0,487961451
Nelfcd	**0,040746411**	-0,931589127
Gm9825;Rnps1	**0,040778481**	1,232631683
Npl	**0,040792512**	0,806037903
Luc7l	**0,040833068**	0,716957092
Mfsd6	**0,040843106**	1,254755656
Apoa1	**0,04085625**	-1,048468272
Casp7	**0,040898089**	-0,694763819
Pnp	**0,040907937**	-0,803513209
Rpl32	**0,040920188**	0,885107676
Bub3	**0,040925466**	1,38645045
Erap1	**0,040984326**	-0,883227666
Lancl1	**0,041048666**	1,174866994
Mrpl48	**0,041072868**	-0,697295507
Rtn4	**0,041093207**	0,696809769
Tuba1c	**0,041113208**	2,878885905
Sh3gl2	**0,041177953**	-0,75110817
Adh1	**0,041242902**	-1,150129954
H2-K1	**0,041498452**	-0,539868673
Golga4	**0,04208642**	-0,735253016
Ptk2	**0,042151468**	-0,681201299
Smarcd2	**0,042664615**	-0,655321757
Hmgn2;Gm6594;Gm16494	**0,042730354**	4,267596563
Ublcp1	**0,043354839**	-0,755832036
Rell1	**0,043540582**	-0,738373439
Gbp2b	**0,043607362**	-1,197898865
Phospho1	**0,043615267**	-2,279228846
Cul5	**0,043681957**	-0,640961329
Sgpl1	**0,043689024**	-0,982479095
Lrmp	**0,043726444**	2,872276306
Ccdc51	**0,043792998**	-1,082413991
Dpysl3	**0,043975721**	0,651519775
Psmd6	**0,044536953**	-0,439629873
Parp14	**0,044538578**	-0,670635223
Gnb2	**0,04460423**	2,499809901
Spg21	**0,044606061**	-1,924441655
Smtn	**0,044813253**	0,887659709
Adh5	**0,044884211**	-0,426019669
Rpl36	**0,044940476**	1,105434418
1700019G17Rik	**0,045007452**	-1,620560964
Slc25a10	**0,045021021**	-0,999839147
Ndufs5	**0,045074627**	1,115752538
Chmp3	**0,045091454**	-0,565975825
Prpf19	**0,045139466**	-0,499071757
Pipox	**0,045142003**	-1,303236008
Rsrp1	**0,045206538**	2,498725255
Endod1	**0,045209581**	1,964183807
Prdx5	**0,045226667**	0,558417002

Table S2.

Col6a4	**0,045295858**	-0,784571966
Nucks1	**0,045563422**	2,104935964
Abcd1	**0,045630724**	-0,883203506
Atp5d	**0,045964706**	1,560153961
Sec61b;Gm10320	**0,046032401**	1,578626633
Pfdn6	**0,046040936**	-0,587399801
Acad9	**0,046087464**	-0,554172516
Arid1a	**0,046108346**	-0,794276555
Pds5a	**0,046154745**	-0,414993922
Cideb	**0,046171761**	-1,262236913
Exoc3l4	**0,046175953**	-1,162048976
Vdac3	**0,046238372**	1,199310303
Nqo2	**0,046243759**	-1,237989426
Trim12c;Trim5;Trim12a	**0,046548621**	-0,742394129
Lrrc16a	**0,046857971**	-0,926128387
Apoc3	**0,046969609**	-3,977083206
Fnbp1l	**0,047031519**	-0,719475428
Myo1a	**0,047080925**	-0,746494293
Lmod1	**0,047098996**	0,657659531
Ppip5k2	**0,047113019**	-0,754199346
Dnajc19	**0,047166667**	-0,562796911
Pum1	**0,047234532**	-0,826681773
Mgst1	**0,047302594**	-1,398954391
Ear3	**0,047370851**	6,334740321
Mrpl4	**0,047651429**	-0,620420456
Rras2	**0,047834993**	0,710918427
Pdcd4	**0,047903134**	-0,899449031
Eef2	**0,047943343**	0,680960337
Ndfip2	**0,047971469**	-1,071377436
Idh1	**0,048011348**	-0,815279643
Ppp2r1b	**0,048079545**	-1,313825607
Eif2ak2	**0,048209335**	-0,967852275
Lmf1	**0,048231312**	1,376603444
Trap1	**0,048299435**	-0,454300563
Ecm29;AI314180	**0,048309859**	-0,47129631
Atp6v0a2	**0,048405063**	-0,619696935
Aco2	**0,048640449**	0,72379303
Apoc1	**0,048762973**	-1,231443405
Timm13	**0,048885154**	-0,794553121
Lamp1	**0,049006993**	-0,786970774
Ddx58	**0,04947486**	-0,579592387
Postn	**0,04962117**	0,450763702
Pbxip1	**0,049690377**	1,40253067
Tnpo1	**0,049713491**	-0,571358999
Arfgap3	**0,04999455**	0,850307465
Aga	0,050062756	-1,271138509
Fnbp1	0,050131148	-0,677406947
Vapb	0,050194787	1,650157293
Gucy2c	0,050199726	-1,709810257
Fcgrt	0,050258953	-0,733259201
Tuba4a	0,050263736	0,975549698
Ppp1r14a	0,050268493	2,832772573

Table S2.

Pex19	0,050328276	-0,44808197
Mp68	0,050332875	1,597424825
Rnasel	0,05039779	-1,009862264
Bag1	0,050467497	-0,839878718
Rp2	0,050537396	-0,555203756
Pdcd6	0,05060749	-0,876881917
Hsd17b4	0,050677778	-0,905937831
Rab3d	0,050695652	1,999736786
Pafah1b1	0,050764626	-0,526280085
Sbds	0,051307172	-0,614035924
Stx3	0,051327001	-1,121767044
Enoph1	0,051336032	-0,73050944
Fam45a	0,051376694	-0,998638789
Slc25a15	0,051380888	-1,176104863
Dazap1	0,051405405	0,889090856
Rpl7	0,051450135	1,221317927
Flrt3	0,051591398	1,028064728
Upf2	0,051672483	-0,909229914
Dap3	0,051804749	-0,73461024
Serpinc1	0,051873184	-0,531175613
Heatr5a	0,051899868	-0,794790904
Defcr20;Defa20	0,051941799	-1,664696376
Urod	0,052010596	0,658822378
Lss	0,052069333	0,592772166
Parp4	0,052079576	-1,253737768
Srsf1	0,052115789	1,700413386
Ftl1;Ftl2	0,052138852	-1,263885498
Ly6d	0,052148738	0,964125951
Cyp4f14	0,052193029	-1,350762685
Cog6	0,052208556	-0,642605464
Wasl	0,052218085	-1,321183523
Exoc2	0,052278447	-1,64553833
Mzb1	0,052287617	0,97389857
Capzb	0,053038008	1,913194021
Slc30a7	0,053107612	-0,817147573
Serpind1	0,053177398	-1,371018092
Entpd7	0,053375163	-0,838511149
Fau	0,053413299	2,046482722
Palld	0,053445026	1,336737951
Far2	0,053483029	-2,842480342
Aox2	0,05365625	-3,188500722
Kif21a	0,054013004	-1,155059179
Smim7	0,054238961	-2,983146667
Psmb4	0,054259067	1,286298116
Xkr9	0,054329442	-2,575997035
Gbp4	0,054525226	-0,896022161
Vps26b	0,054573643	1,011802673
Naa10;Gm16286;Naa11	0,054812903	1,751116435
Ube2l6	0,055109395	-1,270746867
Gla	0,055127086	-0,775003433
Pdap1	0,055180412	1,092500051
Dpyd	0,055192848	-0,705606461

Table S2.

Clcn3;Clcn5;Clcn4	0,055197943	-2,964677811
Slc9a3r1	0,055232529	-0,779261271
Tra2b	0,055263427	1,616770426
Col3a1	0,055292308	1,420875549
AK157302;Isca1	0,055302799	2,007006327
Rpa1	0,055334187	-0,523347855
Gbp7	0,055335025	-1,007317225
Cab39	0,055373248	-0,68445905
Usp5	0,055443878	-0,342664083
Nhp2	0,05599493	4,987787247
Abcf3	0,056115723	-0,847537359
Lrg1	0,056130489	-0,984330495
Pak4	0,056186398	-0,711470922
Ces1d	0,056201005	-1,262921651
Gnaq	0,056222785	1,042146683
F11r	0,056257251	-0,704840978
Asah2	0,056268015	-1,849491119
Cdhr2	0,056285714	-1,222830455
Dffa	0,0563	-0,66626358
Syngr2;Gm20708	0,056328283	-1,621014277
Cnpy2	0,056370463	1,452477773
Stx4	0,056871731	-0,655162811
Cul1	0,056942643	-0,501063029
Gadd45gip1	0,057013733	-1,099277496
Puf60	0,057069652	-0,586044947
Amacr	0,05712795	-1,072612127
Tial1	0,05732852	1,054572423
Serpinh1	0,057370192	0,684633255
Flna	0,05739759	1,082954407
Myo6	0,057440576	-0,612480164
Pde1c	0,057466828	1,526550929
Atp2a3	0,057478908	0,685419083
Slc9a3	0,057482014	1,023724874
Acsf3	0,057490099	-0,872204463
Timm44	0,057536232	-0,482208888
Myg1	0,057561338	-0,407044729
Reg4	0,057605804	1,283205668
Rad23a	0,057650246	-0,670967102
Hist1h1d	0,057655502	1,961102168
Ece1	0,057675545	-2,790506999
Gosr1	0,057675676	-0,616116842
Pfkp	0,057721332	-0,656349182
Map2k1	0,057724551	-0,597300212
Srsf7	0,057745455	1,372319539
Mtmr9	0,057746617	0,728550593
Coro2a	0,057792593	-0,712572734
Tgtp1	0,057815534	-0,698940913
Wars	0,05786403	0,845104853
Shmt1	0,057869939	-0,513204575
Mtdh	0,057885784	0,81748263
Amfr	0,057956204	-0,677890778
Cul3	0,058017136	-0,402750015

Table S2.

Lyplal1	0,058026797	-1,651902517
Kiaa1598	0,058026862	-0,717305501
B3galtl	0,058088235	-2,853913625
Scamp1	0,058097561	1,105203629
Derl2	0,0580978	3,618584951
Gstt1	0,058566308	-0,964882533
Atrx	0,058958383	-0,326032003
Pdzd3	0,059028571	-1,408393224
Gabpa	0,059064439	-0,707305272
Srsf10	0,059098927	1,891563416
Stk17b	0,059103203	0,737965266
Cdh1	0,059173397	-0,69462649
Itga7	0,05934434	0,812956492
Sra1	0,059388954	-0,90558815
Purb	0,059396938	0,823066076
Lsm6	0,059414404	-0,451944351
Mrpl47	0,059458824	-0,60762469
Plcb3	0,059464455	-0,647431691
Bst2	0,059484634	-1,00206248
Gsdmdc1	0,05955503	-0,543046951
Pgd	0,059699883	-0,51453654
Rpl4	0,059769953	0,693824768
Gbp2	0,059868852	-0,676945368
Ccz1	0,060102924	0,393334071
Gm20498	0,060294393	-0,65137736
Golt1b	0,060372526	4,73724556
Stx8	0,06038273	-1,210159938
Pde4d	0,06044289	-0,813294093
Iyd	0,060524362	3,799734751
Ppp6r2	0,060534884	-3,573204676
Rac2	0,060594657	0,58452034
Endog	0,060605505	0,862915675
Hmgb2	0,060675086	-0,495391846
Ces2e	0,060681344	-0,912021637
Smarcc1	0,060744828	-0,859287262
Gigyf2	0,060762125	1,478230158
Zbtb7a	0,060805461	1,063374201
Acap2	0,060808173	-0,452611287
Aifm1	0,060813714	0,752768834
Fhl1	0,06081473	1,08566157
Svil	0,060828829	0,554745992
Ormdl3	0,06083237	3,542927424
Gltp	0,060862302	-0,615351995
Hat1	0,060874715	1,380783717
Ap3m1	0,060877273	-0,342707316
Slc6a20a;Slc6a20b	0,060879819	-2,324510574
Esrra	0,060883295	2,607163111
Btnl2	0,060884793	-1,439641953
Lasp1	0,060885845	1,828751246
Ufd1l	0,060897407	2,551531474
Fabp4	0,060902778	1,037958145
Hibch	0,060931073	0,645234426

Table S2.

U2af2	0,060944128	0,659424464
Hspa4l	0,060951302	-0,505006154
Csrp1	0,060953036	1,339885076
Anapc2	0,060955017	-0,713134766
Eml1	0,061	0,701575597
Psma7	0,061025872	-0,618306478
Prap1	0,061110112	2,431682587
Nup37	0,061449724	-0,678745906
Gnb1	0,061517699	0,840869904
Sgcd	0,061548822	-0,596571604
Ppp1r11	0,061571587	1,28459994
Akr1c19	0,061585825	-1,483643214
Tmem11	0,061625	1,386681239
Man1b1	0,06164	-0,812175751
Lztfl1	0,061654102	-0,542770386
Eps8	0,061667034	-0,715202967
Cdhr5	0,061679731	-1,150344849
Rpl7l1	0,061680968	-0,858390808
Cdv3	0,061693855	3,578384399
Ifi35	0,061708565	-0,462896983
Samm50	0,061735099	0,835050583
Ptms	0,061748879	4,750586828
Lrrc57	0,061748899	-1,121085485
Psma3	0,061762864	-0,437901815
Mrpl45	0,061777283	1,195006053
Igkv5-39;Igkv5-43;Igkv5-45	0,061846154	-2,042988459
Mia2	0,061978022	-2,112768809
Ces2c	0,062010977	-1,179625193
Cct7	0,06204386	-0,444855372
Galns	0,062659365	-0,864056269
Ankhd1	0,06307221	1,960585276
Rpl8	0,063138798	0,884856542
Gpc4	0,063318777	-1,046801885
Pla2g12b	0,063350055	-1,539241155
Wbp11	0,063534783	-0,675906499
Ptprc	0,063551198	-0,438691457
Ap3s1	0,063603917	1,280476252
Eif4g2	0,063826276	-0,542579651
Gak	0,064051392	-0,725891749
Rnf14	0,06406015	-0,703864415
Myo1d	0,064120043	-0,701990763
Pdcd6ip	0,064129032	-0,44161733
Casp3	0,064164859	0,739978155
Sdha	0,064188841	0,676297506
Dhx38	0,064198062	-0,649434408
Pgam1	0,064216216	0,60993576
Cryzl1	0,064267241	-0,946331024
Tfg	0,064285714	-0,612321854
Heatr1	0,06433657	-1,312794367
Tinagl1	0,064355363	1,593064626
Chka	0,064385593	-1,974610647
Pex11g	0,064403397	-1,318725586

Table S2.

Atp2b3	0,064406048	-1,320290883
Hexb	0,064431624	-0,807266235
Polr1c	0,06444773	-2,214039485
Ca3	0,064453871	1,91969045
Btf3l4	0,064471838	1,118312836
Cops4	0,064495197	-0,571889242
Sfxn1	0,064500535	-0,814204534
Ifih1	0,064515856	-0,929358164
Psma4	0,064540426	-0,480340322
Ctnnd1	0,064545842	-0,512292226
Mvp	0,064584127	-0,766939799
Gsta1;Gsta2	0,064609159	-1,193387349
Psmd10	0,064632911	-0,630029043
Cwc22;Gm13695;Gm13697	0,064674394	-0,648171107
Fkbp8	0,064715789	-0,46151034
Dhrs4	0,064877339	-1,043318431
Lgalsl;Pick1	0,064908517	-0,844539007
Dao	0,064944849	-1,636562347
Acly	0,0650125	0,386035919
Txndc9	0,065058824	0,499806722
Cul4a	0,065080292	-0,638242086
Fam84b	0,065133956	-0,770782471
Mtco1	0,065148225	2,748898824
Rpl23	0,065165289	0,743775686
Sipa1l3	0,065186722	-1,096872965
Anpep	0,065205366	0,475298564
Smek1	0,065216301	-0,461912155
Gpr39	0,065232678	-1,224706014
Slc25a20	0,065284519	-1,249247233
Cdkn2aip	0,065300207	-0,581180573
Ttc39b	0,06535288	-1,170302073
Actr3	0,065359916	-2,737773259
Mapk13	0,065367876	-0,629271825
Gm9791;Carhsp1	0,065421384	1,066743851
Mrps28	0,066024742	-1,533112208
Scp2	0,066271605	-1,133259455
Atp5e	0,066339856	0,62932841
Mptx2	0,067103803	-1,007192612
Nagk	0,067162218	-0,858369827
Cstf1	0,067337423	2,122397741
Eef1d	0,067338776	1,527621587
Ruvbl2	0,067385246	-0,385907491
Cyb5r3	0,067406346	-0,608544668
Igtp	0,067407559	-1,693042755
Cndp2	0,067454359	-0,852088928
Siae	0,067608563	-0,731751124
Wasf2	0,067633401	0,821416855
Api5	0,068386572	-0,566843669
Zyx	0,068560976	2,951136907
Slc5a11	0,068596954	-1,145147324
2010002M12Rik	0,068933602	-1,428939819
H1f0	0,068960486	0,574853261

Table S2.

Sft2d2	0,069003021	-0,670342763
Dync1li1	0,069005045	-0,592533747
Tgoln1;Tgoln2	0,069030426	-0,803821564
Ctsc	0,069072581	-0,485748927
Tardbp	0,069074747	1,388399124
Nqo1	0,06914459	-1,379800161
Ppp2r2a	0,069214575	1,053480784
Nudt19	0,069278392	-0,624025345
Rab22a	0,069562249	0,732657115
Acta2	0,069825476	2,263641993
Pfdn1	0,069983968	-0,288194656
Synm	0,070038038	0,897293727
Tomm34	0,070166008	-0,485054652
Tnks1bp1	0,070205384	1,341393789
Cspg4	0,070211254	1,750449498
	0,07023541	-0,733788808
Hddc2	0,070239841	-0,982290268
Coro1a	0,070275449	0,702304204
Ctnnbip1	0,07030495	-0,779797872
Dlgap4	0,070309524	-0,848748525
Sco1	0,070345654	-0,652879715
Dock2	0,070379345	-0,602797826
Psmd11	0,07038408	-0,3793691
Oplah	0,070416	0,896004359
Ccndbp1	0,070449304	-1,065711339
Vamp8	0,070454183	1,419296265
Cnot1	0,070631164	-0,514031092
Aldh3a2	0,070757129	-0,866738637
Smchd1	0,070826772	-0,565133413
Ube4a	0,070896552	-0,83623759
	0,071033399	-0,446673711
Rpl38	0,071053974	1,264673869
Psmc4	0,071329412	-0,359739304
Ppap2a	0,07148728	-1,193191528
Timm23	0,071557297	3,752003988
Tcf25	0,071769306	1,026950836
Rnase1	0,071902344	3,037406921
Ncapd2	0,071921951	-1,570923487
Gstm7	0,072186589	-1,209343592
Fcgbp	0,072256809	-0,93178304
Mafb	0,072265107	1,329774857
Tln2	0,072327167	-0,695045471
Gpkow	0,072745894	1,255898794
Csrp2	0,072816248	2,963793437
Pvrl2	0,072872587	-0,904327393
Vav1	0,07287767	1,115683238
Prkcd	0,072886738	-0,705982844
Faf2	0,072957364	0,859593074
Pigs	0,073028128	-0,786390305
Rps5	0,073154957	1,439231237
Dpt	0,073183908	2,026250839
Ube4b	0,073193858	-0,530787786

Table S2.

Fam173a	0,073225434	1,822561264
Ogfr	0,073254075	-0,578460058
Npc2	0,073264169	-0,536258062
Reep6	0,073296046	-1,413695017
Itpk1	0,073301435	-0,658340454
Echdc1	0,073334615	-0,803115845
Exoc5	0,073349904	-0,641887029
Lmbrd2	0,073409742	0,716485341
Gng2	0,073469466	-0,540301641
Smndc1	0,073527066	1,71032842
Ppp6r1	0,073555977	-0,540231705
Rps23	0,073596958	0,768281301
Ctss	0,073605338	-0,889523824
Sar1b	0,073619048	-0,403778712
Metap1	0,073666984	-0,593153
Atp6v1b2	0,073986755	0,926062902
Clpp	0,074043602	0,800431569
Aldh1a2	0,074056818	-0,660118103
Ugt1a7c	0,074139623	-0,986143748
Chordc1	0,074151229	-0,518814087
Snx12	0,074209632	0,901203156
Ptpn2	0,074344958	0,633705775
Btf3	0,074391714	1,035221736
Efemp1	0,074664158	1,006177266
Eed	0,074721805	-0,809532166
Gm15800	0,074738028	0,780143102
Acot7	0,074795497	0,662768046
Nlrx1	0,074841612	4,562434514
Ces2g	0,075363296	-0,770647685
Gdpd1	0,075487371	-0,703442256
Ube2c	0,075519553	-1,554839452
Cav1	0,075589935	0,786480586
Igkv4-57	0,075645191	-0,617978414
Eftud1	0,075660448	-0,463223139
Mpst	0,07568	0,758391062
C2cd2l	0,075715888	-0,351538976
Smarce1	0,075799257	-0,542469025
Tbc1d8b	0,075825441	-0,516049067
Gpaa1	0,075825926	-0,945646922
Tpm3-rs7	0,075837188	1,377702713
Pak1ip1	0,075851577	-0,800358454
Ccdc58	0,0758962	-0,605838776
Gsr	0,075940905	-0,586109797
Eif1ax	0,075945155	1,570701599
0610037L13Rik	0,07595225	1,013126373
Cmas	0,075970696	-0,448284149
Fxr2	0,07599633	2,156080882
Polr2b	0,076011091	0,44370079
Spcs2	0,076014639	0,889275233
Zranb2	0,076022059	1,814358393
Urb2	0,07604033	-1,336606344
Gk	0,076084871	0,804713567

Table S2.

Dnajc10	0,076091996	-0,449935277
Fbxo3	0,076102004	1,756137848
Aamdc	0,076113242	-0,931056976
Degs2	0,076117972	1,636175791
Tubb5	0,076134668	1,191348394
Vdac2	0,076162063	0,918781916
Manba	0,076171376	-0,869966507
Arl2	0,076240876	-1,331397374
M6pr	0,076618182	-0,631246567
Rb1cc1	0,076681777	-1,195901235
Pfdn4	0,076726612	1,049681981
Ndufa4	0,076751361	-0,568642298
Tomm22	0,076946703	1,167847951
Psmd12	0,077016275	-0,513784409
Ttc7;Ttc7a	0,077085973	-2,33743604
Acnat1;Acnat2	0,077146979	-1,220453262
Lzic	0,077155797	-0,724756877
Pml	0,077189189	-0,436201731
Rps13	0,077216606	0,990708669
Zg16	0,077482944	-1,721083959
Ifitm2	0,0775	1,071400325
Ddb1	0,077552561	-0,519213994
Znfx1	0,077569757	-1,008583069
Golga5	0,077763441	-0,521161397
Tmc4	0,077833184	-1,198502223
Adar	0,077962399	-2,407038371
Ergic1	0,078107335	2,27572759
Mat2b	0,078292335	-0,423859278
Gcc1	0,078362177	-1,124660492
Spag9	0,078380697	-0,447734197
Fam3c	0,078432143	-0,436314265
Dnajc9	0,079462423	-0,527142207
Pi4k2b	0,079516444	-1,152666728
Add3	0,079523979	0,751565297
Optn	0,079532743	-0,714621862
Hcn3	0,079570922	1,994950612
Fbln5	0,079587189	0,924378713
Arf1;Arf3;Arf2	0,079603189	-0,494714101
Bclaf1	0,079641526	0,754449844
Prpf6	0,079658059	-0,445624034
Hgs	0,0799188	-0,400697708
Pnp2	0,079989399	-4,926720937
Akr1e2;Akr1e1	0,080119718	-0,665808996
Pcbp1	0,080169312	-0,583694458
Nckap1	0,080179262	-0,594772975
Fam21	0,080190308	0,674016953
Lrpap1	0,08024978	-0,533009847
Dock8	0,080492576	-0,673945745
Triobp	0,080562937	1,140656789
St14	0,080575944	-0,541113536
Fdps	0,080607018	0,471289317
Supt6h	0,080613497	-0,455607096

Table S2.

Tbc1d15	0,080633421	-0,349796931
Pttg1ip	0,080704028	-0,506031672
Cyp3a13	0,081843206	-0,820359548
Iap	0,081905759	-1,633835475
Aamp	0,08191456	-0,772698085
Mme	0,081928634	-1,037740707
Npc1l1	0,082163478	-0,859190623
Rab10	0,082199826	-0,318255107
Ptbp3	0,082354167	-0,385274251
Gaa	0,082588031	-0,584624608
Pla2g7	0,082823529	1,861016591
Prg2	0,082826575	1,598861059
Dab1	0,082852204	-1,531134923
Lgals3	0,082895238	0,751925151
Crebbp;Ep300	0,0828981	-1,176446915
Nsf	0,082901288	-0,364426295
Psph	0,082926244	-0,678372701
Ints2	0,082967071	-3,485375086
Specc1	0,082972509	-0,711915334
Prss32	0,083043852	-1,120555242
Psmf1	0,083115318	0,835248311
Ict1	0,083151724	-0,571337382
Abcf2	0,083186908	0,550903956
	0,083301967	1,682090123
Myo7b	0,083328192	-0,673455556
Evl	0,083373288	-1,222658793
Mkl2;Mkl1;Myocd	0,083448511	0,673758825
Ndufa12	0,083491901	0,957555135
Uqcrq	0,083519591	1,054520925
AI837181;Bles03	0,083521368	1,346177419
Myl10	0,083545687	2,582544963
Bri3bp	0,08356314	-2,169375102
Hnrnpa1	0,083591837	0,732633591
Erbb2ip	0,083650255	-0,513291677
Prpsap1	0,083721325	-0,734642665
Dock1	0,08383715	-0,63187472
Cyp4b1	0,083841618	-1,777491887
Pex13	0,083881356	-0,745137533
Fez2	0,083898391	-0,757603963
Pcnp	0,08391231	1,991474152
Ipo4	0,083932318	-1,005797068
Trim32	0,083942761	-2,351626714
Hnrnpll	0,083966216	0,395673116
Srsf4	0,083983122	1,463204702
Myl12a	0,084037194	-0,605130514
Flnc	0,084275399	1,040359497
Memo1	0,084302775	4,000252406
Dctn3	0,084346218	-0,44177564
Decr1	0,084567114	-0,783258438
Cog4	0,084830821	-0,491776148
Stat3	0,084856187	-0,570643743
RtcA;Rtca	0,084901928	0,50756518

Table S2.

H2-Q2		0,084927197	-1,701557159
Gpx3		0,084975773	-0,615243912
	Sep-05	0,085205342	-0,60687383
Snrnp200		0,085331126	-0,361134211
Hs1bp3		0,08534	-2,008931478
Exoc8		0,08536311	-0,744883219
Gbe1		0,085379768	1,352414449
Akap1		0,085401823	-0,640282313
Ears2		0,085411176	-1,030968348
S100a13		0,085450622	-1,012756348
Tmem9b		0,085458784	-0,681854884
Mrpl40		0,085497521	-0,753229777
Dctn5		0,085521595	1,618413289
Tpm2		0,085592685	1,642026265
Npepps		0,085663894	-0,519525528
Pgk1		0,086011561	0,424432119
Hnrnpl		0,086306931	1,098185857
Ak4		0,08670404	0,520548503
Cxadr		0,087114403	0,615279516
Tlr3		0,087147541	-1,112639109
Gcc2		0,087186161	-0,531511943
Prpf4b		0,087219032	-0,748589198
Actr5		0,087250616	-1,330102921
Ccbl2		0,087279279	2,379329046
Exoc4		0,087281633	-0,759191513
Cd38		0,087282219	-0,35177803
Gm10020;Rpl15		0,08729064	0,742736181
Nudt9		0,087309329	-0,510004679
Ctdp1		0,087322368	-1,135655721
Phactr4		0,087352941	-0,998908361
Ccdc9		0,087424366	1,883248011
Sephs1		0,087432763	-0,462872823
Smc4		0,087941368	-0,413217545
Mettl16		0,087980472	-1,186934153
Tsr1		0,088267958	0,987888972
Pecr		0,088310428	-0,847320557
Zc3h15		0,088339257	1,168912888
Gnai3		0,08834332	-0,391196569
Ppp2r4		0,088377419	-0,402921041
Cat		0,088381877	-1,568431854
Pip4k2c		0,088406124	-0,503161748
Nme3		0,088414911	-0,510679245
Eif4b		0,088448052	0,97780482
Epn1		0,088469076	-0,923888524
Pdlim7		0,088486618	1,194465637
Fkbp10		0,088519903	1,322974523
Eif3i		0,088534622	1,259031296
Skap2		0,088537409	1,448937098
Nup62		0,088540193	-0,565892537
Dst		0,08859187	-2,448271434
Ehbp1l1		0,088888532	1,515662511
Hnrnpa3		0,088926282	-0,68245252

Table S2.

Hsp90aa1	0,088959872	-0,406614939
Nxn	0,089047238	2,17766571
Olfm4	0,089665867	2,165515264
Lrp1	0,0897376	-0,576145172
1700021F05Rik	0,089796178	-2,458669027
Dimt1	0,089811502	-0,726050059
Eif3d	0,089812995	0,658732732
Agr2	0,089840063	0,602261861
Scarb2	0,089867729	-0,831349055
Rpl13a	0,089884219	0,627059301
Thoc1	0,089939394	-0,440570831
Atp6v0c	0,089955556	-0,634901683
Sgsh	0,089979316	-0,862370173
Fkbp2	0,090006359	1,207150141
Glud1	0,090011173	0,73052152
Hectd3	0,090027006	-0,402393977
Eif4ebp2	0,09085624	1,528635025
Zc3h14	0,090901899	1,053800583
Nln	0,090928063	-0,819178263
Arl8a	0,091318074	-0,840288798
Dsc2	0,092	1,066900253
Gna13	0,092072498	-0,520531972
Acads	0,092107002	-0,34917895
Arfrp1	0,092132075	-0,925830841
Safb	0,09214511	0,453071594
Exog	0,092241569	-1,161149343
Cyb5b	0,092243941	-0,661576589
Ap2a1	0,092266458	-0,516294479
Ldb3	0,092291308	0,755791982
Tbxas1	0,092298507	1,032457352
Trmt11	0,092313972	-0,94117101
Snrnp70	0,092316119	1,457857768
Fads2	0,09243125	-1,09866333
Trappc4	0,092711944	-3,044878006
Tma7	0,092836193	1,43784078
Aldh3b1	0,093063133	-0,77699852
Gba	0,093274143	-0,795630773
Coro1b	0,09341323	0,920312881
Ywhab	0,093427683	0,797411601
Phpt1	0,093451437	-0,576435089
Fgfr1op2	0,09365251	-0,629852931
Ipo7	0,093653251	-0,567979177
Psmb6	0,093661253	0,711678187
Dctn2	0,093724884	-0,460711797
Flnb	0,093725794	0,668052038
Ybx1	0,09379845	1,155272166
Comt	0,093808135	0,41416804
Dnajc13	0,093822086	-0,558385849
Dbr1	0,093854037	1,087060928
Baz1b	0,093854406	-0,404762904
Mipep	0,093871218	-1,065658569
Myh11	0,093880184	1,917058945

Table S2.

Ptcd3	0,093944615	-0,631235758
Ttc37	0,093952344	-0,488461812
Ndrg1	0,093981623	1,062459946
Gata6	0,094016936	-1,031960169
Raph1	0,094070988	1,492358526
Gtf3c1	0,094089368	-0,448101679
Sub1	0,094161912	1,013185501
Sypl	0,094255547	-0,627687454
Kcmf1	0,095041221	1,981004079
Pdlim3	0,09506331	1,231671651
mt-Co3	0,095113827	-1,305196762
Cd2ap	0,095186544	-0,396835327
Dcaf11	0,095917683	2,9399484
Cdc23	0,096085301	-1,532351176
Sptlc2	0,096106545	-0,635945002
Rpl10;Rpl10l	0,096506061	0,825415293
Fv4	0,096526236	-2,239855448
Ahctf1	0,096526874	-1,05875206
Epx	0,096567527	1,275757472
Enah	0,096579227	0,603033066
Elmo3	0,09664085	-0,640479406
Pank1	0,096704992	-1,024641673
Ranbp1	0,096714286	-0,414890289
Trim33	0,096729465	-0,465541204
Col4a1	0,09675	1,098623276
Sin3a	0,096770504	-0,420703888
Itga3	0,096790977	-0,67628479
Coq4	0,096802413	-0,611923854
Cox5b	0,096875472	1,709580104
Klc1	0,096879154	-0,557301839
Hist1h2af;Hist1h2ah;Hist3h2a;Hist1h2ab	0,096952381	1,395296733
Fyb	0,097024793	1,785614014
Heatr5b	0,097147147	-0,604761124
Ralgapa2	0,097524381	-0,618869146
Cyp2c65	0,097529235	-0,91418457
Cog1	0,097728839	-0,611728668
Nedd4	0,098086826	0,736923854
Fam175b	0,098100224	-0,593427658
Slc40a1	0,098212257	-2,172388713
Tor1b	0,098475764	-0,657488505
Armt1	0,098549254	-0,851600011
Rap1gds1	0,098597171	-0,73482577
Mob1a	0,098622853	-0,522413254
Cbx3	0,098666667	0,715023041
Papola;Papolb	0,098670641	-0,473752975
Ints3	0,098818114	-0,750123978
Cfl1	0,098836795	0,919425964
Jagn1	0,098866864	2,915216446
Nop58	0,09889153	-0,328419367
Pycard	0,098940044	-0,423254649
Gip	0,098965056	-2,714659373
Tgfb1i1	0,098994811	1,369655609

Table S2.

Vps51	0,099013333	-0,804463704
Trappc10	0,099435746	-2,283254623
Capg	0,099509239	0,881828308
Ap2a2	0,099743552	-0,48855718
Pspc1	0,099814022	1,844144185
Ndufa6	0,099817109	-0,623378118
Ssbp1	0,099846834	-0,619399389
Slk	0,099873436	0,706094106
Defa17;Defa1	0,100411765	0,94588089
Gm20425	0,100422907	-0,65480423
	0,100496694	1,076552709
Klc4	0,100530792	-0,490211487
Capn1	0,100604549	-0,517156601
Txndc12	0,100709158	-0,441977819
Mrps30	0,100825769	0,952946345
Ngp	0,100828091	2,44554011
Isoc1	0,100838568	-0,659318924
Csnk1g3;Csnk1g2;Csnk1g1	0,100845029	-1,265940984
Ppp2r5c	0,100864234	-1,425207138
Alad	0,100999272	-0,6354599
Arhgdia	0,101072886	0,556073507
Pank4	0,101146608	-0,524669011
Nlrp6	0,101580786	-0,625190735
Pcp4l1	0,101587209	1,329072952
9030617O03Rik	0,101597091	-0,529949188
Mapk14	0,10191582	-0,489526749
Srp19	0,101989833	1,200789134
Soat2	0,102021755	-1,617880503
Cc2d1b	0,102137581	-1,229744593
Ckb	0,102211594	0,931266785
Fgd4	0,102735166	-0,549151738
Taldo1	0,102999277	-0,481160482
Psma1	0,103	-0,388711929
Nufip2	0,103080029	0,595929464
Chmp4b	0,103154401	1,036101023
Ywhag	0,103228881	0,640263875
Ints1	0,103400576	-0,699691137
Dek	0,103781138	-0,47618103
Col1a1	0,104336691	1,416500727
Uchl3;Uchl4	0,104338594	-0,623804092
Rexo2	0,104413496	0,994441986
Lsm4	0,104456165	-0,386030833
Selm	0,104478632	1,539440155
Txn2	0,104488506	-0,755976359
Glod5	0,104500359	-0,857601802
Tsg101	0,10453067	0,341447194
Epb4.1l1;Epb41l1	0,104581246	-0,561503728
Sec23a	0,104586552	-0,474493663
Srd5a3	0,1046	3,151032766
Eif2s3y	0,104605282	1,661019643
Sbno1	0,10465616	1,343890508
Cul4b	0,104668563	-0,799954732

Table S2.

Ppp1r12b	0,104674768	1,180779775
Gid8	0,104731183	-1,858258565
Sorbs2	0,10474306	0,760823568
Cask	0,105060412	-0,527471542
Zmpste24	0,105497872	-0,301514308
Hnrnpa2b1	0,105572747	1,147216161
Limd1	0,105647727	0,805894216
Myadm	0,105882519	2,593439738
Bnip1	0,105936215	-0,487975438
Akr1c14	0,105951909	-0,98587354
Timm8b	0,105957507	1,281712214
Rae1	0,106320848	0,922407786
Isyna1	0,1065	-0,430999756
Arfip1	0,106599859	-0,580221812
Cda	0,106863188	-0,811645508
Gna11	0,107123326	-0,579512914
Tra2a	0,107394366	2,044005076
Eml2	0,107488045	0,814949671
Gsto1	0,107563688	-0,799550374
Arg2	0,107887561	-0,582375844
Pmpcb	0,108190877	-0,50075531
Mlycd	0,108266854	-1,447940191
Cnn1	0,108670407	0,821219126
Vps11	0,108694678	-0,990153631
Man1a1;Man1a	0,108713786	-0,660155614
Lrrfip2	0,108770848	-0,594179789
Ilf2	0,108824598	-0,450750987
Synpo2	0,108900699	0,86918513
Gk5	0,108921788	-1,868586222
Frk	0,10893231	0,850708008
Stxbp2	0,108951185	-0,468873978
Galk2	0,109029758	-0,634461085
Sec14l1	0,109037448	-0,858029048
Trmt10c	0,109052925	0,680544535
Pts	0,109094444	-0,934797287
Rpsa	0,109105263	0,981623332
Golph3l	0,109113116	-0,575265249
Crnkl1	0,109119889	-0,829931895
Slc26a2	0,10912892	-1,152537664
Alox5ap	0,109159944	1,564374288
Myef2	0,109170257	0,763044357
Stx12	0,109235908	-0,5946153
Vimp	0,109732044	-0,878411611
Sorbs1	0,109798064	4,044142405
Ap1m1	0,109807878	-0,419383367
Sec31a	0,110008276	-0,348185221
Dlg1	0,110084196	-0,519637426
Fbp2	0,110089593	-0,692427317
Dlst	0,11015978	0,299636841
Pdlim1	0,110403304	1,0241038
Thoc6	0,110408247	-0,608630498
Myo15b	0,110412655	-0,522699992

Table S2.

Akap7	0,110503775	-2,033852895
Blmh	0,110521262	-0,613218943
Muc2	0,11057967	1,645459493
Rps27a	0,11083756	1,579205195
Stx5a;Stx5	0,110980822	-0,508652369
Ndufa3	0,111323751	2,182051341
Bin3	0,11157377	-0,810621262
Apob	0,111645051	-0,523034414
Itga1	0,111650034	-0,387289047
Rbm42	0,111726402	0,614188512
Adsl	0,111789905	-0,449701309
Kif1c	0,112185412	-0,783636093
Cd63	0,112206242	-1,185492198
Gns	0,11226158	-0,751635234
Mrps9	0,112282417	-0,560604731
Adipor2	0,112358696	-1,292947769
Btnl1	0,112435078	-0,737805049
Wdr36	0,112511565	-0,523305893
Aldh4a1	0,112541272	-0,71637853
Serpinb6a;Serpinb6	0,112545085	-0,77947998
Nudt14	0,112588155	1,388243993
Tspan7	0,112617468	-1,967669805
Adss	0,112693767	0,573067983
Bud31	0,112695064	0,661785126
Camk2g	0,112772973	-0,409164429
Sec16a	0,112920999	0,633406957
Paxbp1	0,113090418	-0,868236542
Preb	0,113265003	0,907388687
Bag3	0,113490566	0,62516276
Bcas2	0,113504711	-0,506216685
G3bp1	0,113511769	0,696673711
Ctcf	0,113581145	-0,472998937
Psmd9	0,113825269	1,698478063
Nosip	0,114143721	2,510560989
Erh	0,114158389	0,706209819
Mgat4a	0,114340483	-2,002847036
Tpm1	0,114358149	1,157142639
Rassf2	0,11503282	-1,816136042
Vkorc1l1	0,115349398	-2,34822909
Pabpn1;Gm20521	0,115355184	1,667316437
Exosc10	0,115601329	-0,553450902
Ndufa9	0,115619681	0,580279032
Map1s	0,115696607	0,356469472
Casp8	0,115708751	-0,514218012
Slc25a24	0,115750332	-0,547369639
Cers2	0,115773635	0,996796926
Stim1	0,115786096	0,356820424
5330417C22Rik;Kiaa1324	0,115850766	1,273691177
Rfk	0,11591461	0,933195114
Exosc3	0,115928	-2,096190135
Bag6	0,115991989	-0,416228612
Kxd1;Uba52;Gm8797;Ubc;Ubb;Gm7808	0,116029197	-0,498320262

Table S2.

Ube2j2	0,116724138	-0,797634761
Ppp2r1a	0,117388999	-0,27206103
Oasl1	0,117466578	0,624857585
Itgam	0,117544371	0,64575386
Cldn7	0,11760582	-0,735427221
Iqgap2	0,118170522	-0,518791199
Cacybp	0,118446499	-0,512183507
Dapk2	0,118671937	-0,559451421
Itgal	0,118750165	-1,338224411
Cwc15	0,118759076	1,508207957
Pde3a;Pde3b	0,118828496	-0,937834422
Prcp	0,119699803	-0,43997256
Pkp3	0,119750164	-0,584721247
Tpd52	0,119828947	-0,422547658
Decr2	0,119923784	-0,898122152
Clpx	0,119984242	-0,509509405
Lmtk2	0,119992136	-1,702850978
Ctnna1	0,12007082	-0,480250041
Anapc5	0,120104575	-1,776254654
Cd36	0,120149606	-0,842153549
Mta3	0,120183126	-0,498456319
Mtpn	0,120206942	0,366118113
Cpsf6	0,12026178	0,756865819
Dcaf8	0,120512084	-0,522324244
Ube2v1;Gm20431	0,120610966	0,601768494
Fabp1	0,120683561	-0,74195226
Rint1	0,120694408	-0,810138067
Napg	0,120722295	-0,442427953
Selo	0,120747567	1,515878677
Ppp1r12c	0,120772934	0,71039772
Sh3bgrl3	0,120778646	1,684699376
Lnp	0,120801044	-0,528006872
Gm9242;Gm8991;Gm6793	0,120825974	1,155252457
Vac14	0,120857329	-0,503797531
Thoc5	0,120866753	-1,162792842
Appl1	0,120944984	0,977307002
Nrp1	0,121023316	1,092204412
Iars2	0,12110175	-0,459414164
Tln1	0,121120623	-0,314614614
Chchd7	0,121362637	1,608235677
Tprn	0,121372093	1,609033585
Afg3l1	0,121381536	-0,81233724
Ufm1	0,122650323	0,948375066
Cog3	0,122659794	-0,49524498
Ugt1a9	0,122738878	2,536875407
Glrx	0,122794591	0,726094564
Fmo2	0,122934363	0,611385981
Mrpl1	0,123490675	-0,836114248
Mgst3	0,123578406	1,779558182
Ugp2	0,123961588	-0,552076976
Slc39a5	0,12396929	-1,492076238
Gramd3	0,123969309	-1,438119888

Table S2.

Pigr		0,124040999	-0,512158076
Cd48		0,124048531	-0,598302205
Igkc		0,124056519	-0,544056575
Tns1		0,124058265	0,50898997
Tgm2		0,124064185	0,728486379
Plxnb2		0,124111392	-0,452699661
Tha1		0,124119974	-0,895865122
Col6a3		0,124120513	-0,798720042
Ube2d2		0,124127796	0,791987737
Zyg11b		0,124136882	-2,099651337
Zdhhc13		0,124200128	-0,382118861
Mfap1		0,124208122	0,875172297
Fam3d		0,124215599	-0,593392054
Hmbs		0,124249842	-0,468034744
Slc25a45		0,124286984	-0,859263102
Ranbp2		0,124306122	-0,235385895
Ccdc22		0,124307497	-0,612024307
Nfu1		0,124320815	-0,501097361
Grem1		0,124328283	0,94564565
Sec31b		0,124341556	-1,174378077
	Sep-08	0,124386523	-0,526742935
Lpar1		0,124392607	-1,331862768
Sec16b		0,1244	-0,72421964
Smim1		0,124406822	1,853808085
Nubp1		0,124465649	1,805844625
Dars		0,124474149	-0,52428182
Mier1		0,124485461	-0,731676102
Cpt1a		0,124487131	-0,533191045
Gde1		0,124565327	-0,820749919
Nt5c2		0,12457071	-1,727078756
Ube2j1		0,124578616	0,731403351
Hmgb3		0,124596977	-0,514352798
Ppp2cb		0,124642409	0,608294169
Mrpl37		0,124657017	-0,439083735
Cald1		0,124675488	2,121192296
Emc8		0,124805016	1,059455872
Minos1		0,125278195	2,142402013
Pmpca		0,125553471	-0,637946447
Alox15		0,125554443	0,864356995
Myl9		0,125633062	0,786079407
Glrx3		0,1258175	-0,351596832
Ppm1a		0,125931293	-0,553143183
Mt1		0,126304619	-1,72482427
Ppp6r3		0,126450405	-0,481657664
Zcchc8		0,126533666	-1,473697662
Ntan1		0,126899813	1,165812174
Nckap1l		0,126973209	1,526339213
	Sep-04	0,126978829	-0,332975388
Plscr1		0,127134328	-0,607616425
Ca1		0,127415786	2,695355733
Cfl2		0,127530726	0,696044286
Ctsa		0,12755528	-0,663454692

Table S2.

Gcsh	0,12782889	-0,563891729
Hsd17b8;H2-Ke6	0,127908189	0,721164068
Oard1	0,128047088	-1,866052628
Pmvk	0,128467822	1,79498291
Ifi205b;Ifi205a	0,128525153	-0,485136668
Gzmb	0,128547368	-0,687476476
Ptk2b	0,128604052	-0,288858414
Ndrg3	0,128619545	-0,829418182
Stub1	0,128638219	2,364429474
Ttc27	0,128644444	-1,65973409
Parva	0,128669549	0,489562352
Uqcrfs1	0,128683047	0,493347804
Mrps22	0,128692972	-0,422672272
Rab5a	0,128698647	-0,375459035
Cd99	0,128736453	1,035401026
Gja1	0,128749073	1,420127233
Ubl7	0,128772363	-0,52073733
Fam3a	0,128777846	1,098450979
Cog7	0,128815773	-1,012372971
Rbm22	0,128956468	1,277975082
Htatsf1	0,128997549	-0,621171951
Krt23	0,129562768	1,423196793
Tpk1	0,130174924	-0,573333104
Ddi2	0,130186047	1,833251953
Prrc2a	0,130211362	0,662080765
Mybbp1a	0,130220049	-0,439235687
Mrpl49	0,130320927	-0,536677043
Gm10639	0,130336996	-1,133473078
Aqp1	0,130609013	-1,052455902
Cct4	0,130688605	-0,279613495
Alyref	0,130736458	0,974545161
Mrpl14	0,130768293	2,226870855
Agps	0,130851582	-0,512575785
Fam195a	0,130981774	1,295087814
Mfn1	0,131061398	-1,300952276
Pex26	0,131097635	-2,013872782
Lrpprc	0,131106796	-0,35324351
Ipo11	0,1311864	-0,564427694
Hspd1	0,131665455	-0,296750387
Pgap1	0,132089588	0,553303401
Actr1b	0,132169594	0,920545578
Vwa8	0,132263603	-0,577835719
Atp1b3	0,132343618	0,625834147
Cpox	0,132357272	-0,605166753
Nae1	0,132437198	-0,595383326
Acin1	0,132454381	0,799924215
Vapa	0,132615199	0,650423686
Kif13b	0,133217601	-0,622324626
Commd1;Gm28048	0,133412048	-1,128419876
Dync1h1	0,133736303	-0,362977346
Prkcdbp	0,13378125	0,585568746
Hdgf	0,133860409	0,350871404

Table S2.

Zcchc6	0,133861696	-1,176610311
Atg5	0,134126126	-0,525431315
Sf3b1	0,134201681	-0,462450663
Cox6b1	0,134258993	0,283259074
Pdhx	0,134265147	-0,313009898
Nupl1	0,134303176	-0,71104304
Slirp	0,134488623	0,555603027
Luzp1	0,13470018	-0,457849503
Ube2m	0,134995816	0,839212418
Wfs1	0,135051374	-0,396208445
Cryl1	0,135076555	-0,706940333
Nop2	0,135491343	-0,456285477
Uck1	0,135546539	0,857379278
Clip2	0,13616935	-0,426096598
Krt14;Krt42	0,1362283	-0,720375061
Seh1l	0,13630934	1,573425929
Tbl1xr1	0,136328767	-1,099113464
Sirpa	0,136390476	-0,890323003
Slc5a4a	0,136410012	-2,90443484
Atp13a1	0,136467933	0,468450546
Rbmxl1;Rbmx	0,136527003	0,805331548
Clta	0,13654902	1,263444265
Actb	0,136652432	0,606267293
Pcid2	0,136687204	-1,020978928
Pex11a	0,136768228	-2,992007573
Srrm2	0,136876258	1,187671661
Suclg2	0,136929627	-0,539364497
Gata4	0,136995272	2,580486298
Slc29a1	0,137010651	-0,598936717
Krt19	0,137304194	-0,396555583
Myo5c	0,137472566	0,661174774
Galnt10	0,137525943	-1,110596339
Rps24	0,137553719	0,678986231
Cyp2j6	0,137558044	-0,767499924
Sae1	0,137672556	-0,344227473
Ttc4	0,138015303	-1,028274536
Mcu	0,138127059	-0,493385315
Smc3	0,138271605	-0,427903493
Cyp4v2;Cyp4v3	0,138343126	-1,23109436
Trmt112	0,138353079	-0,563233693
Rtcb	0,138368545	0,510473251
Snrpg	0,138388726	-0,418886185
Akap2;Pakap	0,138472173	1,140824636
Cct2	0,138552647	-0,414581299
Trip11	0,138553341	-0,505130132
Fxn	0,138554133	-0,683952332
Sec24d	0,13855562	-0,267794927
Pex16	0,138636364	-0,590126673
Gda	0,138676744	-0,570744832
Pafah2	0,138717201	-0,75302887
Acaa1a	0,138745465	-0,562837601
Pa2g4	0,138775131	0,484883626

Table S2.

Parp9	0,138798133	-0,428059896
Sec23b	0,138826698	0,539571126
Nadk2	0,138854471	-0,652941386
Rbbp7	0,138857806	0,326527913
Trim25	0,138879159	-0,408983231
Khk	0,138935673	-0,402839661
Mrps16	0,138938444	-1,055700302
Ndufc2	0,13896028	1,157982508
Rps28;Gm10263	0,139269142	0,973885218
Znf326;Zfp326	0,139366957	-1,153343836
Prps2	0,139440139	-0,5146993
Acbd3	0,139520833	0,356587092
Dstn	0,139544509	0,535532633
Fus	0,139601621	0,777434031
Cnbp	0,139682503	1,211012522
Unc45a	0,140136259	-0,36472257
Slc12a7	0,140217215	-0,57794253
Acy1	0,140364686	2,375192642
Smad4	0,14050519	-1,083786011
Mtmr12	0,140901441	0,853798548
Hist1h1b	0,141676454	-0,644037247
Smap;1110004F10Rik	0,141707373	1,858599981
Thoc7	0,141744534	-0,369455973
Txnip	0,142021852	2,152407964
Ncf1	0,142570936	1,076073329
Xpo7	0,142581609	-0,564229965
Sh3bgrl	0,142966705	-0,400782903
Ap3d1	0,143079748	-0,56291453
Cct8	0,143383028	-0,381727854
Dhrs7	0,143465903	0,677504857
Ptrhd1	0,14366323	-0,835269928
Gls	0,144084717	-0,580380758
Itga5	0,14419189	0,725394567
Rps10	0,144244851	0,636017481
Tacc2	0,144274286	-0,907762527
Ndufaf1	0,144292739	-0,458191554
Slc3a1	0,144447489	-0,901469549
Ubr5	0,144499715	-0,477948507
Ap1s3	0,144551881	-1,44691658
Pex5	0,14462116	-0,525147756
Rabep2	0,144703472	-0,62690417
Trappc8	0,144785877	-0,527493795
Tsn	0,144809117	-0,466665268
Rheb	0,145220647	-0,44887797
Agmo	0,145265306	-1,017875036
Ssu72	0,145300739	-0,649481455
Ncbp1	0,145303065	-0,430857976
Slc37a4	0,145330312	-1,451365153
Thrap3	0,145385576	0,521596909
Serpinb6b	0,145468182	-1,599393845
Raver1	0,145501699	0,772186279
Tubb4b;Tubb4a	0,14571267	0,487578074

Table S2.

Mrpl12		0,145795133	-0,653169632
Abcf1		0,145813273	0,605014801
Acp2		0,145869074	-1,03071022
Lcmt1		0,145885006	-0,360371908
Mospd2		0,145895329	-2,832003911
Sart3		0,145906162	-0,423801422
Lrrc40		0,145915398	-0,649904251
Rps8		0,145918919	0,641868591
Cald1		0,14595144	0,709085464
Hars2		0,145963842	-0,468784332
Eif6		0,146001127	-0,417015076
Chmp2a		0,146012339	-0,321843465
Rab35		0,146053812	-0,685343424
Ncln		0,146055087	-0,473000844
Psmb5		0,146094276	1,37426885
Mmaa		0,146119101	-0,477190653
Nampt		0,146176305	-0,460579554
Plin2		0,146231933	-1,261576335
Cox6a1		0,146493013	0,77919515
Mfap5		0,146505593	2,108736674
Nat10		0,146524902	-0,585000356
Ivns1abp		0,146529609	-1,667775472
Tomm40		0,146606943	0,534812927
Lsm7		0,14678057	-0,565715154
Ppie		0,147035714	1,06095314
Drg1		0,147087563	0,931218465
	Mar-06	0,147440357	-1,048596064
Apex1		0,147721604	-0,290024439
Rfc4		0,147745961	-0,449223836
Arg1		0,147993326	3,053967158
Cttn		0,148017807	0,948539734
Gm20390		0,148504725	-0,591177622
Sf1		0,14856	0,499797821
Por		0,148790672	-0,51140213
Tax1bp3		0,148807543	-0,7348423
Cad		0,148835457	-0,496851603
Copb1		0,148855876	-0,476516724
Ndufs6		0,148890122	1,961605072
Ndufs4		0,149249169	1,063772202
Snx18		0,149311222	1,940961838
Gdpgp1		0,149333333	-1,615386327
Thoc2;BC005561		0,149340696	-0,530437469
Gpx1		0,149361326	-0,603849411
Sec11c		0,149393805	1,257516861
Tars2		0,14947648	-0,348419189
Gbp6;Gbp10;Gbp9;Gbp8		0,149932708	-0,451723735
Emd		0,149969129	0,677202861
Aldh9a1		0,15031405	-0,449324926
Sars		0,150436123	0,745910009
Serpina3k		0,150883875	-0,837018331
Aldob		0,151185919	-0,452711741
Ctps2		0,151474437	-0,663551966

Table S2.

Tspan31	0,152368825	-0,721321106
Hnrnpa0	0,152379178	0,9936409
Cln6	0,152410313	1,587263743
Scyl1	0,152452499	-0,325190226
Fscn1	0,152462719	1,951762517
Col5a2	0,152468132	1,348860423
Ifitm3	0,152577365	0,958793004
Ovca2	0,152660832	-0,499238332
Emc6	0,152692223	-0,65568161
Slain2	0,15274439	2,255396525
Top1	0,152928962	-0,384894053
Ehd4	0,153092299	-0,281634649
Arhgap5	0,153200873	-0,445862452
Rcn3	0,15350245	0,864425023
Cstf2t	0,153529412	-0,802832921
Rab18	0,153613079	0,475618362
Emc2	0,153624796	1,096565882
Tyk2	0,153629226	-0,675457637
Fnta	0,15364539	-0,429533641
Macf1	0,153679522	-0,516177495
Adck3	0,153708379	-1,644392649
Dsg2	0,153713043	0,861834844
Cstf2	0,153878393	-1,168682734
Crkl	0,154047568	0,541506449
Isy1	0,154057328	-0,342360179
Dtx3l	0,154070232	-0,612335841
Krr1	0,154116631	2,000823339
S100a9	0,154140693	1,441750209
Ept1	0,154214787	1,831005096
Mrpl38	0,154218123	0,486040751
Lum	0,154224147	-0,456639608
Rnpep	0,15424607	0,314664841
Cpped1	0,154257855	-0,644292831
Ddx5	0,154262473	0,763490041
Inadl	0,154304207	-0,89544932
Ripk3	0,154330997	3,02634112
Mad2l1	0,154635776	0,716028849
Gnb2l1	0,154916532	0,647061666
Dnaja2	0,154930032	0,577136358
Mrps36	0,155147929	1,111392975
Unc45a	0,155344086	1,180266062
Raly	0,155369099	-0,480824153
Esr1	0,155392378	-0,959260305
Acp1	0,155475832	-1,001078924
Slc4a1ap	0,155519571	-0,536327362
Bag5	0,155559377	-0,459982554
Mthfd1	0,155826367	-0,318770091
Prkaa1	0,155931441	-0,398400625
Fadd	0,156408994	-0,635123571
Syp	0,156548267	1,467953364
Ncam1	0,156579989	-0,601860046
Mup1;Mup15;Mup7	0,156616205	1,354317983

Table S2.

Ahsa2	0,156631804	-0,98633194
Heph	0,156660256	-0,540166855
Hist1h1e	0,15671543	0,899380366
Slfn9	0,156743987	-1,598814646
Bysl	0,15675508	-0,820809682
Dgat1	0,156762108	1,092007955
Eps15l1	0,156779553	-0,316523234
Prosc	0,156797017	0,666436513
Paics	0,157053191	0,971950531
Wdr12	0,157305688	-1,398464839
Aspscr1	0,157409139	-0,533413569
	0,157523101	-0,746117274
Cox17	0,157728526	-0,2872715
Col1a2	0,157751592	1,392682393
Ap1b1	0,157812202	-0,386642456
Ace2	0,158408055	-0,568195979
Arl6ip1	0,158473266	-0,791077932
Tmem263	0,158483342	-0,512345632
Cisd3	0,158485169	0,909111023
Cnbp	0,158524313	1,175345103
Safb2	0,158567196	0,571399689
Tsr2	0,158889124	-1,082153956
Tceb2	0,158901215	0,563206991
Mars2	0,158978364	1,008021673
Txnl4a	0,159303797	-0,541673024
Rgn	0,159504742	-0,875972748
Myo5b	0,159520252	-0,365937551
Hypk	0,159534001	1,835825602
Cox7b	0,159604211	0,697221756
Mina	0,159688257	0,811063766
Rbck1	0,159852787	-0,669509888
Ubqln2	0,160062959	0,862167358
Ctsb	0,160088189	0,446951548
Svs2	0,160172269	0,840315501
Pold1	0,160232826	-0,483897527
Prkag1	0,160256437	-0,555018743
Khdrbs3	0,160528025	0,914218903
Dag1	0,160612159	-0,314909617
Psmd5	0,160975916	-0,541102091
Prpsap2	0,161157509	-2,081785202
Rcc1	0,161443515	1,252613068
Ints6	0,161643492	-0,602261861
Hras;Nras	0,161826541	-0,451751073
Mtus1	0,162003133	-0,567219416
Tmbim6	0,162125261	-0,607142131
Cbr3	0,162136387	-1,087269465
Atp8b1	0,162220833	-0,443770091
Spns1	0,162254432	0,820916494
Osbpl2	0,162275611	-1,443174362
Ugt1a6;Ugt1a6b	0,162305367	-1,977954229
Abhd2	0,162339071	-2,048220952
Mmgt1	0,162360042	2,468734105

Table S2.

Noc2l	0,16249896	-1,399019877
Pik3c2a	0,162641039	-0,569388707
Ppp1r21	0,162803738	1,150258382
Tmem205	0,16516805	-0,697928747
Srp14	0,165253762	2,402463913
Snap47	0,165511664	-0,524054845
	0,165803109	0,88395373
Prorsd1	0,166016563	-0,425909042
Gapvd1	0,166032108	-0,53900973
Serpinf2	0,166086867	-0,692087809
Mrpl39	0,16610124	0,401424408
Cpne2	0,166172788	-0,923247655
Sptbn1	0,16618708	-0,315935771
Sord	0,1662754	-0,875246684
Txndc17	0,166313725	-0,540587107
Hpcal1	0,166399587	-0,511194229
Mbnl2	0,167490722	0,578716278
Cse1l	0,167497166	-0,414081573
Mtfr1l	0,167653965	2,390890121
Smpd4	0,168323212	-0,834771474
Actg2	0,168446502	0,844376882
Ykt6	0,169277121	0,644510905
Lace1	0,169923947	0,594984055
Rpap3	0,170778234	0,766570409
Tmx2	0,170865948	0,278821309
Stxbp3	0,171282051	-0,396540324
Dock5	0,171369933	-0,529786428
Scamp2	0,17138083	0,955063502
Eif4h	0,171518443	0,652472814
Glipr2	0,17190179	1,185730616
Dctn1	0,171903738	-0,309975942
Snrpf	0,171916198	-0,570879618
Kank2	0,171940695	0,447851181
Gdi1	0,171942682	0,453615189
Rars	0,171995914	-0,358697255
Mgea5	0,172081674	1,551549911
Ptges2	0,17234898	0,39522934
Ggt5	0,172416793	0,572982788
Ppa2	0,172427263	-0,457450231
Cope	0,172440592	0,452314377
Wdr1	0,172471784	-0,354417165
Apoh	0,172504049	0,673933029
Afg3l2	0,172515013	-0,376624425
Des	0,172560162	0,586015066
Fbl	0,172591392	0,401625315
Spag7	0,172602851	-0,569842656
Tfam	0,172676426	0,436976115
Ogdh	0,172678825	2,170717875
Cml5	0,172690779	-1,202245076
Anxa7	0,17275813	-0,394720713
Pabpc1	0,172760848	0,416613261
	0,172763636	2,102956772

Table S2.

Prkg2	0,172766346	-0,807451884
Stat2	0,172778797	1,326474508
Rdh11	0,172793156	-0,503807704
Tapbpl	0,172794341	1,387759527
Eif3j1;Eif3j2	0,17279716	0,448071162
Slc35b2	0,172823766	1,703018824
Alg5	0,172843058	0,612965266
Hexim1	0,17288483	1,10334905
Snrpd2;Gm5449	0,172910831	-0,496614456
Rbm17	0,172930653	0,452008565
Hp1bp3	0,172971342	-0,400351842
Kiaa0195	0,172972589	-1,928295771
Ank3	0,172997984	0,491716385
Inpp4a	0,173026727	-0,537567139
Atp5c1	0,173060437	0,342967987
Krt7	0,173066265	0,559059779
Krt4	0,173100954	-1,467131933
Tmem245	0,173461114	-1,623416901
Ubap2l	0,173527054	1,618247986
D8Ertd738e	0,17353661	1,166692734
Spata5	0,173548322	-0,807877858
Pdzd11	0,173561905	-1,736353556
Lbr	0,173784	0,556893667
Mien1	0,173821822	-0,540363312
Lrba	0,173870935	-0,363929113
Atg4a	0,173953023	-0,965703328
Tmem236	0,174160759	-1,500962575
Matr3	0,174247752	0,359006246
Magt1	0,174253493	-0,456579208
Aldh1b1	0,174596911	-0,393057505
Cbx5	0,174667331	-0,538286845
Acox1	0,174683948	-0,592526754
Calu	0,174715212	0,537981669
U2surp	0,174879841	-0,36983935
	0,174966716	-0,703337351
Mtnd5	0,174989055	0,554021835
Map2k2	0,175018417	-0,473398844
Atp2b1	0,175053678	-0,409047445
Acat2;Acat3	0,175079065	-0,315706889
Mrpl11	0,176059553	-0,550404231
Igha	0,176230159	-0,466737111
Actr1a	0,176440258	0,283084869
Dnttip2	0,176864222	0,715912501
Fam160b1	0,177022288	-0,493464152
Glod4	0,177970297	-0,261442184
Irgm1	0,178335477	-0,556690852
Ctu1	0,178354105	1,108058294
Zzef1	0,178442688	-0,517372131
Ctps1	0,178481737	-0,262980779
Fam134c	0,178485418	1,402772903
Atox1	0,178506167	-0,776582082
Filip1l	0,178512315	0,797676722

Table S2.

Cops3	0,178527764	-0,436524073
Atp6v0d1	0,178536681	-0,432372411
Ptgs1	0,178560158	0,453626633
Csf1r	0,178564405	-0,507488251
Eef1d	0,178569877	0,615591049
Slc25a13	0,178591244	-0,573279699
Prpf38a	0,178600296	0,461562475
Ywhae	0,178679134	0,462231954
Ogt	0,178882671	-0,606742859
Arpc1a	0,17897053	0,529886246
Stam	0,179436979	-0,542482376
Fkbp1a	0,179458292	0,674420675
Macrod1	0,179531373	0,63720576
Igfbp7	0,179631553	1,564632416
Dscr3	0,179774951	-0,440521876
Esrp1	0,179816227	-0,579491933
Acy1	0,179862947	-0,473136902
Rbm25	0,179904156	-0,263462702
Tradd	0,179951028	-0,535287221
Usp7	0,180084025	-0,437536875
Dcn	0,1807255	-0,399888357
Col4a2	0,180730112	0,753153483
Atp5b	0,180813659	0,303576152
Gap43	0,180818359	-0,539993922
Nceh1	0,180931774	-0,980795542
Lyrm5	0,181148978	-0,833658218
Rrp1	0,181192401	-0,943290075
Ica;1300017J02Rik	0,181204866	-0,523423513
Ssr4	0,181366375	1,344140371
Mapre3	0,181454545	1,286968867
Mrpl17	0,181458961	-0,607662201
Pygl	0,18148249	0,422458649
Actn4	0,181970874	-0,301383972
Luc7l3	0,182068899	0,411223729
Ezr	0,182286822	0,404394786
Tnc	0,182375182	-0,383942286
Rnf213	0,182413191	-0,270584106
Lamp2	0,182615682	-0,482463837
Gopc	0,182643445	-0,457569758
Mtnd1	0,182647942	1,414972941
Rabif	0,183046422	1,920131683
Exosc1	0,183702415	0,958888372
Fgl2	0,183791203	-0,733884811
Spcs1	0,184225978	0,983385722
Phka1	0,185546332	-0,800703049
Akap13	0,18602219	1,955973307
Cnnm4	0,186484089	-0,591108322
Faah	0,186527938	-0,494171143
Gstm1	0,186617831	-0,870262146
Slc15a1	0,186906115	-0,566476186
Acad11	0,187035611	-0,550643285
Mrto4	0,187126923	-0,403546651

Table S2.

Slc35a3	0,187216931	0,440120061
Cd44	0,187965401	0,508326213
Bcl2l13	0,188335249	0,470410665
Man2b2	0,188366076	-0,894058228
Arpin	0,188370797	0,890327454
Hnrnpm	0,188425084	0,604629517
Ckmt1	0,188448921	0,397277832
Psmd7	0,188449761	-0,480131785
Hmcn2	0,188456376	1,033754985
Pik3r1	0,188474088	-0,402128855
Eps8l3	0,188505041	-0,553450902
Gps2	0,188669216	-0,615368525
Psme4	0,188700143	-0,505460739
Gpr107	0,188746896	-0,539405187
Prkcsh	0,188837076	-0,31480662
Tmed4	0,188885919	-0,304066976
Mcm6	0,189017176	-0,364230474
Lmcd1	0,189088137	0,897514343
Cotl1	0,189134414	0,681480408
Acp1	0,189175012	-0,358695984
Gipc2	0,189226667	-0,615635554
Fmo4	0,189300333	-0,981946309
Twf2	0,18934855	0,281836828
Anp32b	0,18943863	-0,413659414
Tle3;Tle2;Tle1;Tle4	0,189528517	0,770771027
Ddx18	0,189681214	-0,357302984
Vbp1	0,189700474	0,816848119
Ipo8	0,189712387	-1,058867772
Wdr33	0,189790422	-0,461559931
Eppk1	0,189802469	-0,257092794
Kng1	0,189892637	-0,525932948
Rpl19	0,190268119	0,420058568
Zpr1	0,19075947	-0,675397873
Tufm;Gm9755	0,190870344	0,434827169
Rplp1	0,190898441	1,065474828
Dcps	0,190915958	-0,372625351
Lgals4	0,190927796	-0,478736877
Stmn2	0,190960377	-1,632350922
Rpl3	0,190988658	0,613985697
Slc25a1	0,190991008	-0,584951401
Prkaca	0,191072848	-0,380151749
Serhl	0,19107896	-0,892344793
Gmfb	0,191234684	1,822582881
Tmem30a	0,19158926	-0,512310028
Zbp1	0,192035782	-0,959249496
Vps13c	0,192372706	1,998607635
Atp5a1	0,192933208	0,209203084
Pkn2	0,193082707	-1,760374705
Cd82	0,193173484	-0,733410517
Ctage5	0,193981291	-0,451754888
Uqcr11	0,193995303	3,855166753
Ptrh2	0,194017782	1,087633133

Table S2.

Ubtf	0,194065543	-0,514554342
Tmx3	0,19409274	-0,405334473
Naa25	0,194173832	-0,387158712
Tubal3	0,19417909	-0,74682045
Hk1	0,194183693	0,245227178
Nup93	0,194195236	-0,32187144
Atp1b1	0,194222222	-0,364557902
Xpot	0,194251866	-2,070643107
Gtf2h4	0,19426461	-0,406532923
Rrp12	0,194270169	-0,617179235
Rab3gap1	0,194276408	0,563587189
Gdi2	0,19429398	0,404071808
Stip1	0,194294747	-0,33690834
Hdgfrp2	0,194301115	-0,352617264
Rtn3	0,194311737	-0,588523229
Cdc73	0,194314219	0,371459325
Ddx17	0,194331163	0,323741277
Anks4b	0,194345599	-0,592212041
Emp2	0,194361333	-0,949911118
Atad1	0,194366853	-0,402984619
Dkc1	0,19443616	-0,388453166
Rpl37a	0,194446818	0,94740804
Ephx2	0,194485383	-0,508307139
Agpat9	0,194575673	-0,556706111
Dnajc1	0,195482375	1,512189865
Shpk	0,195647659	-0,734647115
Gfer	0,195759148	-0,871539434
Nfs1	0,19578684	0,815840403
Uap1	0,195857473	-0,366721471
Tmem43	0,195864815	0,546938578
Acsl5	0,19613136	0,428005219
Ranbp10	0,196146093	-0,75598526
Tppp3	0,196327172	0,592809677
Sf3b2	0,196480148	-0,344851176
Creb1;Crem;Atf1	0,196570901	-0,510410945
Sep-07	0,196822499	-0,185284297
Sdpr	0,196862021	-0,333073934
Pfdn5	0,196882543	-0,440758387
Rfc5	0,196913284	0,878137589
Arf4	0,196930876	-0,517535528
Got2	0,197053407	0,36575826
Shmt2	0,197491026	-0,551810582
Tnpo3	0,197529412	-0,459344228
Sts	0,19762023	0,929374059
Spint2	0,197644465	0,572351456
Ehd1	0,197670653	-0,48837471
Taf6	0,197954128	-0,984659831
Prelp	0,197959596	-0,368806839
Gatb	0,197966071	-0,445896149
Csnk1d;Csnk1e	0,197988068	0,36490949
Hnrnpk;Gm7964	0,198047663	3,404436111
Serpina1b	0,198512821	-0,395775477

Table S2.

Arhgef1	0,198556115	-0,360075633
Pbld1	0,198910755	-0,964968363
Pfkl	0,198975297	-0,377340317
Sec24b	0,199513933	0,359002431
Dctn4	0,199548446	-0,324565887
Park7	0,199588477	0,480496089
Eci2	0,200195434	-0,60060819
Acadm	0,200359325	-0,566084544
Cpsf2	0,200388864	-0,363353729
Ap3b1	0,200399635	-0,298266729
Mgat3	0,200685506	-0,735612869
Armc6	0,200687016	-0,521924337
Etl4;Skt	0,200876138	-0,44490242
Rab21	0,201665908	0,726877848
Rbbp6	0,201969063	0,375862757
Myh10	0,202058182	-0,36836942
Stk24	0,202079055	-0,318947474
Trappc1	0,202105359	-0,554681142
Hmga1	0,202150068	0,831110001
Snw1	0,202546279	0,483133316
Rps17	0,202591012	0,466303507
Mylk	0,202608617	0,427233378
Gstt2	0,203341795	-0,539072673
Copg2	0,203626643	-0,371735255
Atxn7l3b	0,203992754	0,911068598
Ddx3x	0,204764147	0,31795756
Trip12	0,205422624	0,939259211
Dnpep	0,205438263	-0,557763418
Uba7	0,205448463	-0,472358704
Acsl3	0,205686399	0,4057223
Ubqln1	0,206031603	-0,367830912
Spr	0,20603252	0,394016902
Brd2	0,206050542	-0,579985301
Rabgap1l	0,2062553	-0,673763275
Ddx19a;Ddx19b	0,206301172	-0,329678853
Ctsz	0,206386661	-0,51236852
Ggact	0,206636036	1,169942856
Srpk1	0,206885187	-0,931568782
Bax	0,207164716	-0,302691142
Map1lc3a;Gm5612	0,207198562	0,316529592
Stx17	0,207210787	-0,436751684
Igkv4-55;Igkv4-72	0,20721868	-1,634403865
Abhd14b	0,207233468	2,777832667
Abracl	0,207251799	0,42688179
Cstf3	0,207287892	-0,554499308
Ube2z	0,207380888	1,660497665
Ubr2	0,207447781	1,940303802
Dcakd	0,207473968	-0,635365804
Gsn	0,208958781	0,80144755
Rbbp5	0,20925571	-0,872562408
Mgl2	0,209416294	0,789265315
Tmem33	0,209748546	-0,537986755

Table S2.

Gmppb	0,209817531	0,397628784
Sacm1l	0,209891819	0,293787638
Smc2	0,210293119	-0,348091125
Pdcd10	0,210370701	-0,687270482
Mrpl22	0,210433929	-1,787916819
Fam3b	0,210909415	-0,807376226
Wwp1	0,210966994	1,196022669
Mcfd2	0,211283103	2,653294881
Erp44	0,211513369	0,489994685
Ftsj3	0,21162244	-0,674861908
Pnpt1	0,211666815	-0,411184311
Ak2	0,211674232	-0,291634878
Ttc38	0,212259671	-0,37491099
Dut	0,212298932	0,766139984
Utrn	0,212630831	-0,239918391
Rbp7	0,212631111	-0,503096263
Tpm1	0,213383659	1,48227946
Osbpl3	0,213450998	-0,909954071
Taok3	0,213499556	-0,392705282
Gtf2f1	0,213544607	0,776956558
Hspg2	0,213942452	0,254210154
Srp9	0,213996457	0,813991547
Ndufa10	0,214035461	0,274827321
Mrps31	0,214091272	-0,609757106
Srsf3;Gm12355	0,21459823	0,409957886
Adck5	0,214690845	-0,336438497
Ttc19	0,214788683	1,235665639
Vkorc1	0,215202828	0,811361313
Slc25a35	0,215475265	1,05603536
Tcp1	0,215506825	-0,290185293
Cystm1	0,215588884	2,320989609
Apool	0,215601762	0,574205399
Cldn15	0,215633157	-0,877599716
Dpp3	0,21564519	-0,232116699
Slc26a6	0,215647422	-0,40688324
Dock11	0,215740397	-1,474538167
Tbk1	0,216448336	-0,533508937
Spryd4	0,216543145	-0,619378408
Slc2a2	0,216605101	-0,668169657
Idh3b	0,216638037	0,233969371
Gm6576	0,216639438	1,693155924
Eci3	0,216640563	-1,317058563
Mboat7	0,216681338	-0,471186956
Trim30d;Trim30a	0,216687418	2,210697174
Ptgr1	0,21672967	-0,441855113
Nit1	0,216734622	-0,473457336
Gfpt1	0,216767003	-0,339558283
Txndc5	0,216782456	0,571606318
Gm561	0,21686216	-0,687746048
Spint1	0,216937883	0,984708786
Smim20	0,216973304	1,185278575
Raly	0,217348491	0,54111735

Table S2.

Tpi1	0,217520979	0,202467601
Scgn	0,217662882	-1,593987783
Son	0,217757973	0,782320023
Pdcl	0,218283719	0,939470291
Ganab	0,218352531	-0,437586466
Cela1	0,218740515	1,59322993
Col5a1	0,21899041	1,303965251
Kdm1a	0,219038328	-0,448823929
Acot8	0,219133769	1,010247548
Vps35	0,219277318	-0,416994095
Dars2	0,219528285	-0,559652964
Dnajb4	0,219874728	-0,288016637
Gm5435;Gtf2a1	0,219907826	1,278003693
Tomm20	0,220281494	1,349526723
Usp3	0,220377227	-0,347284953
Dync1li2	0,220519323	0,561911265
Exosc9	0,220944444	-0,407429377
Sugt1	0,221938395	0,339570999
Hnrnpdl	0,222560278	0,520953496
Dnajc11	0,223193758	-0,387906392
Myl12b	0,223308492	0,669055939
Slc2a4	0,223320918	0,820553462
Atp6ap2	0,223548052	1,407445908
Reep3	0,223608827	-0,440791448
Rfc2	0,223814879	-0,401702245
Ptpn23	0,223851275	-0,383514404
Dopey2	0,224058773	-0,528994242
Snrpb2	0,224074234	0,417095184
Osbpl11	0,224170984	-1,093257268
Atp8a1	0,224236411	1,542985916
Ocln	0,224267819	0,805562337
Mdm4	0,224670112	-0,688812256
Ddx20	0,224766911	-0,472040812
Nrf1	0,224815517	-1,16467158
Scrn2	0,224853816	-0,59797287
Ckm	0,225124408	0,696975708
Cuzd1	0,226095484	2,276081721
Selenbp1;Selenbp2	0,226134251	-0,41798528
Lyar	0,226493551	0,334444682
Itgb6	0,226827675	-0,865882238
Aars2	0,228819588	1,291695913
Focad	0,22923143	-0,766471227
	0,229244101	-1,698924383
Dhrs7b	0,22928927	-0,270725886
Tpm1	0,229466552	-0,562372843
Ltn1	0,229696918	-0,702369054
Mapk1	0,229795289	-0,316687266
Mthfsl;Mthfs	0,229844835	-0,82117335
Slc7a8	0,229849186	-0,438567479
Atad2b	0,230368849	-0,695737203
C1galt1c1	0,230516681	-0,546608607
Srxn1	0,230556648	1,634991964

Table S2.

Vamp3;Vamp2	0,230653009	0,606108348
Igkv8-28	0,230742418	-1,493553797
Defa8;Defa3;Defa10;Defa16;Defa13;Defa	0,230751494	1,401801427
Alcam	0,230841026	-0,445762634
Tspan8	0,230986348	-0,380690257
Ghitm	0,231186701	0,683356603
Ces1c	0,231237527	-0,660840352
Rbp2	0,231394972	0,417794545
Tmem176b	0,231582624	-0,483836492
Tm9sf1	0,23212415	0,316283544
Slc37a2	0,232215803	-3,306381226
Sult2b1	0,232217595	0,476437887
Rbm26	0,232222884	-0,429676056
Pld3	0,232321702	0,995019277
Znf598	0,232338868	-0,425584157
Xpo5	0,232346497	-0,348199209
Ash2l	0,232521001	-0,439509074
Wdr45b	0,232543294	-0,475081126
Vat1	0,23293469	-0,201779683
Acadvl	0,232973294	-0,342283885
Itm2b	0,233011864	-0,821178436
Hnrnpab	0,233829733	0,577787399
Slc44a1	0,233933136	-0,462706248
Cobll1	0,233984759	0,936970393
Wdr92	0,234370558	0,949680328
Pcyox1l	0,234851586	2,421410878
Ccdc115	0,235634827	-1,291223526
Nagpa	0,235807432	-1,614029566
Lyn	0,235859738	0,424267451
Myo1e	0,235994935	-0,392948786
Bcar1	0,236038819	-0,885009766
Ldlr	0,236328975	1,96942838
Tsfm	0,23651602	-0,347230911
Golga7	0,236726507	-0,595375697
Ppia	0,237142376	0,495295207
Eif3e	0,237254737	-0,391462326
Gstz1	0,237586207	-0,571828842
Hmgb1	0,237600337	-0,329223633
Golim4	0,237618487	-0,596333186
Nt5c3a	0,237648148	-0,332932154
Cyc1	0,237718369	-0,305065155
Psmd13	0,237880772	-0,372053782
2310035C23Rik;Kiaa1468	0,23789509	2,134452184
Ndufv3	0,237937001	0,892185847
Lmf2	0,238389262	0,784088771
Cnih4	0,238642079	0,894273758
Pon2	0,238651572	-0,375291189
Ak1	0,239033096	0,474265416
Rab27a	0,239680067	0,389469147
Muc3	0,240075345	-0,703751882
Fah	0,240324686	1,385491689
Rps15a	0,240610623	0,47100385

Table S2.

Ypel5	0,240961137	0,813768387
Pygb	0,240963211	0,227432251
Pgm5	0,241040936	0,477821986
Mrpl50	0,241070564	-1,052366892
Pak2	0,241358932	-0,305338542
Ndufb10	0,241435127	0,339796066
Thop1	0,24146789	-0,458719889
Smarcb1	0,242121667	-0,443809509
Iws1	0,242165902	-0,46747462
Hn1l;AY358078	0,242302122	0,926937103
Sec24c	0,242305706	-0,365561167
Pfas	0,242346378	0,336704254
Atp5j2	0,243024106	-0,539394379
Tmod1	0,243076539	-0,49899737
Sycn	0,243078586	1,713021596
Ikbkap	0,243099294	-0,320503871
Nup153	0,243350498	1,410044988
Sf3a1	0,243479702	-0,326118469
Rpl22	0,243580605	0,499446869
Ccdc86	0,243605307	-0,579332987
Esd	0,243642236	-0,500057856
Tyms	0,2436978	1,750325521
Cand1	0,243706346	-0,253514608
Rps6ka4	0,243807469	-0,38849322
Sp100	0,243877483	-0,367430369
Chtop	0,243940471	0,713581721
Mrpl53	0,243978521	0,510592779
Letm1	0,244033058	0,345259349
Drap1	0,244041356	-0,568508784
Slc25a3	0,244142325	0,375460307
Psmc5	0,244422442	-0,242179235
Ltbp4	0,2444327	1,614152908
Setd3	0,244523318	-0,275801341
	0,244791753	-0,685417175
Cnot6l	0,245502061	-0,843623479
Atp6v1c1	0,245722291	-0,334555944
Tap1	0,245891269	-0,472349803
Synj2bp	0,245911898	-0,625501633
Plbd2	0,245955556	-0,417612076
Itga6	0,24603125	-0,213846842
Ormdl2	0,246076512	0,720030467
Akap12	0,24626716	0,387919744
Man2c1	0,246366475	-0,541002274
Npc1	0,246444353	-1,603766123
Fndc3a	0,246714813	0,717153549
Cdk5	0,246778325	-0,483435949
Golga3	0,246835111	-0,528901418
Atg3	0,247589996	-0,330260595
Aldh1a7	0,247909836	-1,222867966
Chchd2;Zbed5	0,248315102	0,936972936
Mpp7	0,248342997	0,654314041
Slc1a5	0,248444444	1,989796321

Table S2.

Ctnnbl1	0,248451349	-0,396458308
Pgam5	0,2484953	1,241404215
Eif4a3;Gm8994	0,248502249	-0,382531484
Ints7	0,248520458	0,698108673
Vim	0,248530713	0,550479889
Morf4l1	0,248535407	0,447120031
Gimap4	0,248580987	-0,464768092
Ggps1	0,248581729	-1,148146311
Apoe	0,248797716	-0,475467682
Ogdh	0,248843049	0,315910339
Wdr26	0,249281174	-0,554292043
Mrps10	0,249373523	-0,444949468
Aqp7	0,249579805	1,441319784
Dnm1	0,250304312	-0,398976644
Tuba1b	0,250347296	0,371590932
Dhcr7	0,250406186	-0,360079447
Map2k3	0,250422764	-0,368429184
Sdsl	0,25140512	0,328702927
Serping1	0,251465692	-0,515805562
Prrc2c	0,251530463	0,43488884
Slc2a5	0,251561688	-0,527505875
Mcur1	0,251985396	-1,028390249
Nipbl	0,252422843	-0,44400088
Gm15284	0,252425911	2,268710454
Hint1	0,252474878	0,898614883
Mrps26	0,252532036	-0,669977188
Hsp90ab1	0,252535063	-0,285437266
Rnf40	0,252770538	1,375888189
Bid	0,253176375	-1,913012822
Mtmr1	0,253266478	-1,578139623
Pddc1	0,25352404	1,001625061
Dhrs3	0,253626516	-1,154297511
Myh11	0,2536956	0,483268738
Gamt	0,253798061	2,948013306
Gcdh	0,25434544	-0,411020915
Hspa8	0,254347721	-0,215502421
Commd4	0,2546	0,54239591
Plrg1	0,254805802	-0,464166005
Znf148	0,254866586	0,956384023
Timm50	0,254968989	-0,371458054
Golph3	0,255025765	-0,35716629
Ccar1	0,255074447	-0,237294515
A430005L14Rik	0,255198713	0,3591067
Engase	0,255747487	-0,298196157
Rab9a	0,256282845	-0,377556483
Kpna2	0,256336013	-0,280919393
Syvn1	0,256846586	-0,602847417
Ptpmt1	0,256976315	-0,479946772
Gatm	0,257301243	3,203164419
Gm8730	0,257324238	2,291557312
Rbm19	0,257417234	-0,821661631
Ppif	0,257465918	-0,266994476

Table S2.

Arfgap2	0,257515419	0,499027888
Chmp1b1	0,25756891	-0,31678772
Canx	0,257801441	-0,309857686
Ociad1	0,257867947	0,495687485
Rpa2	0,2583648	-1,765103658
Acaca	0,258597361	-0,433076223
Clasp1	0,258704428	-0,593355815
Ctdspl;Ctdsp1	0,258807662	-1,156346003
Chmp1b2	0,258814815	0,333651225
Ano9	0,25882494	-0,423683802
Gripap1	0,258841226	-0,402772903
Bin2	0,258910978	0,482385635
Rcor1	0,258917928	-1,247550329
Pcyt1a	0,258926635	0,419858297
Gpr108	0,258931845	-1,243149439
Tmem97	0,258944268	1,681590398
Dnajc7	0,258979233	-0,419231415
Ube2v2	0,259082701	0,752327601
Ikbip	0,259397217	1,06250445
Itln1	0,259455847	0,412216822
Isg15	0,259699762	-0,416086833
Cyp4f16;Cyp4f37	0,259726659	-0,588585536
Rps11	0,259750397	0,826945623
Nup54	0,26008092	-0,230620702
Usp39	0,260115919	-0,439237595
Drg2	0,260122125	0,337095261
Acot13	0,260142857	-0,537644068
Itgax	0,260167854	-0,354139964
Rab3a	0,260258423	0,803398768
Hcfc1	0,260270891	0,370080312
Mfn2	0,26037401	-0,455319087
	0,260759494	-0,307503382
Dhodh	0,260816779	-0,564750671
Cp	0,261153025	-0,481919607
Qrich1	0,26118419	-0,36228625
Narf	0,261209008	-0,367355982
Tiprl	0,261684959	0,417609533
Fkbp11	0,261753555	2,482242584
Cnpy3	0,262089976	-0,392482758
Strn4	0,262169625	-0,860327403
Cela2a	0,262334385	1,388931274
Aldh1l1	0,262836879	-0,347976049
Hmg20b	0,262841151	-1,484869639
Fgb	0,262853543	1,679248174
Dynlt3	0,262867271	0,386405945
Rufy3	0,263307359	-0,550806046
Kpnb1	0,264511408	-0,293173472
Lamb1	0,264570979	-0,289749146
Pithd1	0,265646226	-0,643576304
Ush1c	0,266196386	-0,316359202
Ppp4c	0,266300982	0,492881775
Ranbp3	0,266695447	-0,410373052

Table S2.

Thyn1	0,266800157	0,58378919
Hdac1;Gm10093	0,266846184	0,509739558
Gnpda1	0,266918623	-0,518246969
Mst1r	0,266937304	-0,821589788
Arfgef1	0,266939192	-0,3723526
Ighv8-12	0,266950666	-1,600753784
Tox3;Tox;Tox4	0,266993336	-0,49444898
Ahnak	0,267025801	-0,246955872
Gm2a	0,267044706	-0,685009003
Aldoc	0,267055229	0,788885752
Etfb	0,267130231	-0,375220617
Fga	0,267399766	1,372355779
Pla2g1b	0,267400234	1,238955816
Clca3b	0,267414102	0,921365738
Myl1;Myl3	0,267504688	-0,612641017
Capn2	0,267518316	0,331996282
Fbln2	0,267535283	1,572825114
Slc25a22	0,267547229	-0,3960584
Tmed1	0,267555382	1,936976115
Plcg2	0,267659774	0,189788183
Hmox2	0,26817134	-0,279109955
Ppih	0,268501167	-0,391227722
Gclm	0,268523161	-0,297012329
Lactb2	0,268726566	-0,334672928
Kars	0,269255054	0,358665466
Nt5c3b	0,269292929	-0,347694397
Nucb2	0,269301205	0,782042821
C3	0,269373204	0,561363856
Rpn1	0,269635093	0,538733164
Lsm3	0,270716835	-0,466960907
Atxn2l	0,270730307	0,309737523
Itih1	0,270892594	-0,420260111
Trnt1	0,27091938	-0,323090871
Fam50a	0,270933747	0,533082962
Alg6	0,27125639	-0,908928553
Max	0,271413086	-0,595080058
Naa15	0,271537152	-0,168318431
Ipo5	0,271616248	-0,328601201
Bpnt1	0,272802474	-0,38007609
Tnik	0,272907966	-0,662094752
Sfrp1	0,272978362	-1,457009633
Dcun1d1	0,273511008	-0,339300791
Gbas	0,273601544	0,441841125
Numb	0,273627171	-0,330132167
Cyp2c68;Cyp2c69;Cyp2c40;Cyp2c67	0,273990741	-0,818296432
Leo1	0,274412649	-0,506425858
Pitpnb	0,2748151	0,569503784
Rps6	0,274921002	0,395119985
Abcd3	0,274960339	-0,358078003
Asna1	0,274982267	-0,383661906
	0,276170901	1,185554504
Syf2	0,276523278	-0,634044011

Table S2.

Napa	0,276614912	-0,377967834
Lsp1	0,276619048	2,025042852
Grn	0,276673587	0,819091797
Chp1	0,276725317	-0,338118871
Slc11a2	0,276727692	-1,456335068
Clptm1l	0,276870633	0,63180542
Dcaf7	0,277001918	0,802946726
Naprt	0,277065234	-0,361475627
Rhog	0,277651207	0,483748754
Smim24	0,277661043	1,062201182
Strn3	0,277947893	-0,253080368
Eci1	0,278016086	-0,33382225
Naaladl1	0,278284621	-2,507792155
Psap	0,278305396	-0,332473755
Pabpc4;Gm10110	0,278321593	0,801326752
Hnf4g	0,278366068	-0,97327741
Zc3hav1	0,278472477	0,585865657
Mdh1	0,278531549	-0,454341888
Alb	0,279148741	-0,584148407
Khdrbs1	0,279161832	-0,283798218
Agl	0,279233295	0,482273102
Tmem65	0,279255246	-0,704446157
Sparc	0,279300229	0,676314036
Erlec1	0,27947236	1,350589116
Atp5l	0,279698171	-0,252339045
Smap1	0,28048992	-0,457438151
Yif1a	0,28053445	-0,971385956
Ndufb8	0,280549467	0,435756048
Tbcb	0,28064128	0,789419174
Ccdc132	0,280705524	-0,784627914
Mrps24	0,281654753	0,719601313
Slc38a10	0,282221209	-0,755187988
Cisd2	0,282582067	0,350854238
Mrpl33	0,283287505	0,928563436
Lxn	0,283442673	-0,578341166
Tomm5	0,283760243	-0,522392909
Tbc1d9b	0,28376926	-0,345989863
Nasp	0,283769435	-0,396942774
Arpc5	0,283827142	-0,415801366
Ythdf3	0,283847028	1,491873423
Abat	0,283954545	-0,585447947
Znf207;Zfp207	0,284062145	1,991687775
Snap23	0,284470499	0,434604645
Sp1;Sp4;Sp9;Sp8	0,284484298	1,010712306
Nrd1	0,284498107	-0,27879715
Pofut2	0,285019282	-0,605585098
Ube2h	0,285909297	-0,451307933
Apoo	0,286707971	0,602884293
Stx7	0,286813137	0,501628876
Snx3	0,28692145	-0,31696256
Tcea1	0,287673962	-0,36205101
Psmb1	0,287966805	0,286762873

Table S2.

Arpc1b	0,288553336	0,52275149
Tpmt	0,288571644	-0,500610987
Uchl1	0,288610399	0,179868062
Rab8a	0,288741243	-0,484446208
Apaf1	0,289159639	-0,843987783
Tf	0,289174878	-0,327380498
Emc3	0,289193378	0,583740234
Cox5a	0,289196543	-0,363435109
Smap2	0,289258647	1,02355957
Exosc4	0,289302221	1,48021698
Ap1m2	0,28965077	0,280255636
Nudcd2	0,289756757	-0,295577367
Yes1	0,289759579	-2,009953181
Immt	0,289924203	0,669065475
Yars2	0,290412603	-0,179559708
Ap2m1	0,290627672	0,42224884
Naca	0,29066317	0,788208644
Eef1b;Eef1b2	0,290686657	0,479703267
Rps21	0,291328839	0,718627294
Cdc5l	0,291471359	-0,222463608
Rpl17	0,291621257	0,36403211
Ncoa5	0,291655817	-0,413005829
Tuft1	0,291902767	-1,642778397
Ppp1r10	0,293127477	-0,49270312
Cdc37	0,293550075	-0,28687795
Eif2d	0,293924542	-1,083390554
Tmpo	0,294455564	0,522078196
Aldh1l2	0,295533532	2,983573914
Guk1	0,295534676	-0,396268209
Cirh1a	0,295549085	0,805018743
Nup160	0,295562687	1,182918549
Ighv3-6	0,295563501	-1,115117391
Stk39	0,295571802	-0,417220434
Adpgk	0,295597466	0,695877075
Ap1g1	0,295748325	-0,376640956
Fkbp3	0,295803498	-0,385803858
Ythdf2	0,29589736	1,773391088
Lima1	0,29600744	0,417821884
Ighv3-5	0,296178439	-1,084306717
Vps28	0,296249721	-0,412665049
Keap1	0,296414254	-1,927993139
H2-Eb1	0,296524322	-1,167392095
Sf3b5	0,296601783	-0,413005829
Exoc3	0,296922849	-1,637152354
Osbpl10	0,297033024	1,603359858
Smpd3	0,297505376	-0,331886927
Ubqln4	0,29767235	-0,256676992
Qsox1	0,297676176	-0,299157461
Fuk	0,29901037	-0,340429942
Susd2	0,299240281	-0,283714294
Glg1	0,299289415	-0,256786982
Itpa	0,299464497	-0,290776571

Table S2.

Ppp1cc	0,299486496	0,477790197
Parp10	0,299902403	1,149490992
Nova2	0,300805617	1,16027832
Syap1	0,301271787	-0,289014816
Cel	0,301341707	1,68547821
Gucy1b3	0,30151052	0,715356191
Srpr	0,301791882	0,72572581
Ero1lb	0,30249502	1,166233063
Aldoa;Aldoart1	0,302586067	0,314959208
Cpne1	0,302663717	-0,303303401
Scg2	0,302751658	-0,367480596
Plscr3	0,303029098	-0,609518051
Ppp3ca	0,303125184	0,28631719
Tcof1	0,303446855	1,043345769
Hspe1	0,303459897	0,30839475
Mtnd4	0,303480309	0,788731893
Fam162a	0,304185226	0,215527852
Cpsf1	0,304195445	-0,269384384
Usp47	0,304264706	-0,243684769
Col6a5	0,305100917	0,409288406
Psmd2	0,305174743	-0,229434331
Ctsl	0,305236871	-0,335574468
Lta4h	0,305540719	-0,255536397
Polr2e	0,305786891	-0,695237478
Golgb1	0,30586432	-0,291280746
Nptn	0,305898901	-0,24762853
Rer1	0,305961158	-0,390054067
Dctpp1	0,306032258	-0,449090322
Epdr1	0,306150805	-0,707279205
Rrp9	0,306269203	0,766081492
BC017643	0,306302232	-2,417022069
Wibg	0,306331261	0,221365611
Ppp3r1	0,306657895	-0,396225611
Hpx	0,307009134	0,421285629
Ptma	0,307103322	-0,242459615
Snx9	0,307140979	-0,28831927
Snrpd1	0,307494353	-0,839407603
Impa2	0,307498177	1,01413854
Txlna	0,307508567	-0,221984227
Abi1	0,307524781	0,469685872
Psmb9	0,307546151	-0,557326635
Plekha6	0,307560408	0,976093292
Hnrnpul2	0,307613139	0,222141266
Nat6	0,307624181	0,936701457
Yipf3	0,30767237	-1,569558462
Slc5a1	0,30829247	-0,50739034
Rps20	0,308410033	0,435849508
Fam49b	0,308522182	-0,23106575
Hsdl2	0,308549219	1,187014898
Fam213a	0,30856686	-0,824742635
Cmpk2	0,309129993	-0,426897049
	0,309474601	0,529027939

Table S2.

Bckdha	0,309488575	1,199047724
Impad1	0,309547498	0,566390991
Mon2	0,309586933	-0,322664897
Fut8	0,309632476	-0,303447723
Apbb1ip	0,309736232	0,911190669
Psmb10	0,310881565	0,846041997
Lap3	0,310893956	-0,293730418
Paip1	0,310916727	-0,518056234
Asl	0,310920405	-0,348884583
	0,311084268	0,653812408
Cgn	0,311135526	-0,182191849
Sh3glb2	0,311172813	-0,547039668
Prps1l3;Prps1	0,311257225	-0,316682816
Fibp	0,311891658	-0,53768158
Abhd12	0,312046176	0,39099884
Ceacam18	0,312064958	-1,363665899
Hspa9	0,312098195	-0,27200826
Cdk9	0,312911648	1,036800385
Rbm15	0,313084355	-0,421307882
Nsfl1c	0,313183423	0,515548706
Slc25a4	0,313196975	0,339302063
Aars	0,313309798	-0,183861415
Top2a	0,313540677	-0,248812358
Glo1	0,313989205	-0,367399216
Naip1	0,314202878	-1,630325953
Ppig	0,314353448	0,646231969
	0,314356912	1,480305354
Ubap2	0,314429033	1,124223073
Stam2	0,314501797	-0,304053624
Uaca	0,314614887	-2,202730815
Srek1	0,31467911	-0,674242655
Pxn	0,31581916	0,253899256
Cbl	0,316747489	-0,934856415
Ddx3y	0,316803155	-0,496042252
Rrm1	0,317035125	-0,333641052
Hsd17b12	0,317798638	-0,377598445
Akap8l	0,318768349	0,41316096
Znf22	0,318816619	0,667070389
Reg1	0,319166786	1,178120931
Supt5h	0,319544902	0,483459473
Mettl7a1;Methig1;UbiE2;Mettl7a2;Mettl	0,320054363	0,492643992
Pcsk1n	0,320205861	-0,383391698
Atl2	0,320238827	-0,294687271
Ighg1	0,320380136	-0,745033264
Ethe1	0,320516589	0,298353831
Tbc1d17	0,32059	1,219917933
Lrrc47	0,320630978	0,181793213
Commd3	0,320641026	-0,56466039
Epb41l2	0,320672844	-0,258467356
Dap	0,320705882	1,191827774
Ddx41	0,320713267	-0,53174909
Capzb	0,320745448	0,299341202

Table S2.

Hist1h4a	0,320755255	0,4116834
Spg20	0,320760413	-0,361719131
Ctsd	0,32077153	0,715766907
Usp24	0,321548845	-0,53907903
Itih3	0,321577525	-0,293327332
Uqcrb	0,321599431	-0,216206233
Mvk	0,321619623	-0,422166189
Sqrdl	0,321663113	0,221403758
Ech1	0,322160511	-0,204295476
Ahsa1	0,322708304	0,469774882
Nup205	0,32272437	-0,220097224
Sort1	0,322778843	-0,59835434
Uba5	0,322957816	0,536654154
RBM8;Rbm8a	0,32298406	0,30119578
Slc4a4	0,323041135	-0,544041316
Clasp2	0,323098512	1,10244751
Ppp1r9b	0,323137394	-1,796445847
Prmt5	0,32340531	-0,345546722
Cnp	0,324287332	-0,466334025
Thnsl2	0,324646393	-0,416706085
Hbb-b2	0,324713123	2,118039449
Pdia5	0,324869565	-0,384618123
Pi4ka	0,32490318	0,587317149
Rab14	0,324928294	0,19776535
Rraga;Rragb	0,325597458	-0,31638972
F12	0,325610152	-0,756912231
Gabarap	0,325722751	1,093927383
Rnmt	0,325724965	2,045512517
Acp6	0,325748765	-0,413362503
Rps14	0,325819979	0,436418533
Sh3gl1	0,325918252	-0,275093079
Mrps34	0,326015482	-0,371725718
Sec22b	0,326130236	0,302820841
Sult1d1	0,32614019	-0,413615545
Ighv1-42;Ighv1-43	0,326169014	-0,664193471
Krt15	0,327175272	-1,486846288
Vipas39	0,327195782	0,508916855
Aco1	0,327242446	-0,184408188
Ddx46	0,327254309	-0,234692256
Cdc42bpb	0,32731083	-0,223541896
Hook2	0,327373815	-1,782554626
Mlf2	0,327374035	1,151613235
Banf1	0,327397472	-0,452925364
Hdhd2;Ier3ip1;Gm10784	0,327593125	0,499809901
Smad2	0,327795231	0,405971527
Lrrfip1	0,327816334	0,24559021
Cd74	0,327831815	-0,688552856
Naga	0,327956567	-0,265357971
Eif3l	0,328088235	-0,283512751
Plaa	0,328533427	-0,293345769
Cnn2	0,328707198	0,338488897
Arhgap1	0,328736179	0,281163534

Table S2.

Eif4g3	0,328782943	-0,581509272
Fgg	0,32889262	1,297663371
Nploc4	0,328897902	0,168949763
Hnrnph3	0,32950751	-0,490820567
Pdia6	0,330147295	0,53284963
Necap1	0,330148045	-0,426919937
Exoc7	0,330343336	-0,389180501
Fis1	0,330369027	-0,266992569
Mink1	0,33066016	-0,702565511
Hic2	0,330671777	-0,859745661
Cox6c	0,330736402	0,441244125
Kif5b	0,330760348	-0,176995595
Tmtc3	0,330837274	-0,381771088
Atpaf1	0,330875435	-0,376516978
Ivd	0,330942897	0,525047302
Chmp2b	0,330947442	-0,236586253
Pelo	0,331021944	-0,380422592
Ube2g2	0,332230796	-0,321365992
Nat8	0,332436414	-0,837607702
Arrb1	0,332677778	-0,32637914
Gcat	0,332750261	1,281730016
Gcnt3	0,33403331	1,073919296
Gsk3b	0,334106213	0,398402532
Usp4	0,335235784	0,212681452
Ubxn1	0,335281304	0,288040161
Cpsf7	0,335338648	-0,638808568
Cox16	0,335891892	-0,713384628
Gnl1	0,335936266	-0,629791896
Prpf4	0,336969529	0,305185954
Fech	0,337049498	-0,313757579
Crip1	0,337088897	-0,375634511
Psmb2	0,337175087	-0,293076833
Nup35	0,337608575	1,12313652
S100a4	0,337698309	0,865736643
Ano6	0,337729692	-0,633367538
Zc3h18	0,337758454	0,62061882
Mat2a	0,337771942	0,285974503
Cant1	0,337814917	-0,175880432
Pik3r4	0,337884629	-0,627391179
Tdp1	0,338784828	-1,176250458
Derl1	0,33890169	-0,478621165
Khsrp	0,339215713	0,295401891
Dtna	0,33925732	0,725336711
Pard3	0,339268847	1,008457184
Gosr2	0,339273228	0,412055333
Ddx21	0,339292011	0,333914439
Rtkn	0,339332644	0,986589432
Pdcd11	0,339749656	-0,714349111
Stk10	0,339866529	-0,488924662
Dhrs1	0,339944998	0,493041356
Eif4e2	0,340200687	-0,50341479
Armc1	0,340685675	1,199197133

Table S2.

Irf2bp2	0,341248626	1,400110245
Pde2a	0,341288019	0,772384008
Glb1	0,341562114	-0,35879008
Asah1	0,342379417	-0,327582041
Samd9l	0,343256516	-0,32691129
Ciao1	0,344281111	1,508708954
Selt	0,34507745	-0,330043793
Msh6	0,346258904	-0,489038467
Ubr4	0,346377527	-0,281475703
Mark2	0,346444368	-0,264403661
Rpl7a	0,347249829	0,400155385
Rhob	0,347325351	-0,723642985
Pgm1	0,347398291	-0,275773366
Aspn	0,347436389	0,44165357
Umps	0,347506662	-0,350248973
Hspa14	0,347583049	0,666067759
Mdh2	0,347673497	-0,34319814
Hgh1	0,347759645	0,46333758
Sema4b	0,348095563	-0,326589584
Galnt2	0,348279768	0,464876811
Diap1;Diaph1	0,348313779	-0,225839615
Bcar3	0,348826458	-0,493701299
Misp	0,349828221	0,669055303
Tmed7	0,350202385	0,341798147
Rnf20	0,350237057	-0,287133535
Chmp1a	0,351305886	0,573764801
Gabarapl2	0,351425459	0,274150213
Ero1l	0,351442969	0,338762919
Psmc2	0,352053043	0,342882156
Chmp5	0,352142857	-0,217667262
Igbp1	0,352216179	-0,193604787
Dbi	0,352881494	-0,297515869
Dpp9	0,352888587	0,253355662
Denr	0,352926945	0,459692001
Acot2	0,352978622	0,734607061
Ube2n	0,353098439	-0,182099024
Lipa	0,353290366	-0,529339472
Sfxn3	0,353489318	0,209300995
Tcf7l2;Lef1;Tcf7l1;Tcf7	0,353906441	-0,376581828
Cfdp1	0,354133965	0,918317795
Dync1i2	0,354253807	0,22856458
Ppme1	0,354309726	-0,415525436
Ints4	0,354373731	-0,482411702
Cog2	0,354493735	-0,305154165
Ccnt1	0,354536585	0,595647812
B3gnt3	0,354550372	-0,497450511
Pycrl	0,35462834	-0,262789408
Fam129b	0,355390541	-0,301006317
Nap1l4	0,355510645	-0,134990692
Prdx1	0,355775751	-0,312328339
Slc25a5	0,35584605	0,172063192
Nme2	0,356255147	-0,562093099

Table S2.

Lama4	0,356777328	-0,239490509
Dnajc5	0,356816189	-0,405925751
Mtmr3;Mtmr4	0,357000674	-1,369274775
Dlat	0,357237197	0,202875773
Plbd1	0,357322548	-0,308308919
Tom1	0,357574941	-0,595753352
Fam213b	0,357615876	-0,446239471
Numa1	0,357702357	1,07065773
Acbd5	0,357736205	-0,507455826
Cdk11b	0,357777179	-0,476401011
Psip1	0,358713277	0,250061035
Rbbp4	0,358769334	-0,25763003
Cops5	0,358869624	0,238369624
Picalm	0,358977837	-0,262790044
Crat	0,359093656	-0,41327858
Gtf2b	0,359098421	1,565951665
Pklr	0,36186443	-0,368621826
Ago1	0,362140221	0,540686925
Clybl	0,362190476	0,19300588
Tagln	0,362724774	0,26757431
Timm10	0,36330429	-0,268639247
Fer	0,36360201	-0,529671987
Smpd2	0,363819156	-0,364510854
Sccpdh	0,364127175	0,400772095
Cmc1	0,364249079	-0,22931544
Oxa1l	0,36491268	-0,367736816
Mut	0,365071572	0,391930262
Vcl	0,365088599	-0,175735474
Pcmt1	0,365275401	0,267011642
Hdac2	0,365673674	0,699151993
Chac2	0,365748831	-0,974245707
Camk2d	0,365773623	0,238091787
Rap2a	0,365795728	-0,414045334
Nolc1	0,365871032	-0,512706121
Igkv8-27	0,365886591	-1,19417127
Trabd	0,366212738	0,514070511
Otub1	0,366514324	-0,185440063
Twf1	0,366537821	-0,254677455
Ncl	0,366588	0,372006734
Coq3	0,366909091	-0,283185959
Lamtor3	0,367079893	0,622386297
Ap1s1;Ap1s2	0,367137438	-0,449383418
Xpo4	0,3679002	0,432944616
Dnajc2	0,368441636	-0,253612518
Eefsec	0,368702757	-0,290967941
Mup4;Mup9;Mup8;Mup6;Mup14;Mup1[illegible]	0,368772606	1,324664434
Fubp1	0,368790698	0,2243735
Fbxo25	0,368882685	-1,007635752
Gmpr2	0,369104914	0,83672905
Gpd1l	0,369407162	-0,369373322
Pcmtd1	0,369473614	-0,413317998
Fam160a2	0,369529685	-0,635626475

Table S2.

Anp32e	0,369617784	-0,328261058
Cdc42bpg	0,370096122	1,367126465
Idi1	0,370648559	0,289259593
Wbp2	0,370771372	1,616439184
Mccc2	0,371146543	0,512749354
Brd1	0,371269358	0,489277522
Wdr61	0,371351208	-0,532034556
Sec61g	0,371437086	0,840976715
Hadha	0,371791005	0,268951416
Gbf1	0,372058182	-0,309552511
Ctrb1	0,373748843	0,880123138
Tpd52l2	0,374002639	-0,267451604
Eif2s1	0,374023778	-0,263577143
Ubl5;Gm16381	0,374031054	-0,262357712
Strap	0,374126031	-0,254676819
Ehhadh	0,374213579	-0,333179474
Chmp4c	0,374215182	0,958128611
Arhgef5	0,374226477	-0,465906779
Polr2h	0,374282702	0,384046555
Prkag2	0,374296076	1,673087438
Apeh	0,374387352	-0,433192571
Adgre5;Cd97	0,374422127	0,378006617
Slc12a9	0,374707895	-0,768496831
Tmem59	0,37473511	0,244839986
Naa50	0,37485846	1,039704641
Mrps17	0,374888524	1,079573949
Ube2r2	0,376410256	0,812366486
Itih2	0,376701708	-0,418729146
Eif2a	0,376716398	0,337862015
Rap1b	0,376885386	0,298439026
Srsf6	0,377709783	0,441480637
Hnrnpu;Gm28062	0,377715223	0,334457397
Fbn1	0,377780112	0,429913839
Fam114a1	0,377814365	-0,412163417
Psmd3	0,378171803	0,237341563
Erp29	0,378203868	-0,261604309
Mif	0,378471513	0,267341614
Ywhaz	0,378570118	0,217903137
Calr	0,37859548	-0,228983561
Nfkb1	0,380133508	-0,274807612
Ripk1	0,380144026	-1,081195196
Ebna1bp2	0,380520772	-0,338019053
Ndufa5	0,380886275	-0,44713974
Leng9	0,381010788	-0,594784419
Ccdc186	0,381054284	-0,442747752
Chchd3	0,382827834	0,440781275
Msn	0,382856956	0,241279602
Hbb-b1	0,383132986	2,462658564
Fermt3	0,383188254	0,269402822
Igf2bp2	0,383215405	-0,28524971
St3gal6	0,383242009	0,532205582
Ssr3	0,383257907	0,569192251

Table S2.

Apmap	0,383302644	0,264422099
Zw10	0,383734115	-0,471863429
Sel1l	0,384085993	0,38163503
Vps36	0,384178444	-0,327886581
Dhx29	0,384576635	-0,40331459
Bccip	0,384627604	-0,933945974
Cbr4	0,384720416	0,226851781
Chp2	0,38476878	-0,309270859
Lamb2	0,384783344	0,335309982
Strn	0,387002925	0,575938543
Csnk1a1	0,387454722	-0,283638636
Myh14	0,38751039	-0,253348033
Mtch2	0,387624431	-0,20132192
Fahd1	0,387636246	-0,3606294
Diablo	0,387875406	-0,207667669
Supv3l1	0,38841518	-0,402452469
Dgkq	0,388486066	1,030059179
Rabggta	0,388567909	-0,319056829
Copa	0,388575875	-0,209097544
Coq5	0,388921283	0,432832082
Csnk2a1	0,389050518	-0,294895808
Bag2	0,389319521	0,811527888
Mrpl46	0,389524426	0,415799459
Aim1l	0,389650485	-0,477830887
Mcm4	0,389856404	-0,280857722
Lsm12	0,390738203	-0,413250605
Smpdl3a	0,390864533	-0,514420827
Pnlip	0,390966074	1,190531413
Adtrp	0,39103876	-0,75590833
Traf6	0,391467872	0,472357432
Gstt3	0,391916075	0,398196538
Tmpo	0,392486452	0,448322296
	0,392494034	-0,428430557
Ebp	0,392613101	-0,295941671
Rabl6	0,392994197	0,388177872
Fam114a2	0,39303126	-0,297118505
Serbp1	0,393090206	0,289243062
Ptpn12	0,393433816	0,473763784
Cenpv	0,394591567	0,427474976
Got1	0,394625885	0,299238841
Aldh16a1	0,395075933	-0,270433426
Rfx1	0,395297523	-0,874802907
Stoml2	0,395872669	0,395925522
Reg2	0,396041144	1,22150294
Cstb	0,396546272	-0,316118876
Cds2	0,396986829	-0,271389008
Jph2	0,39735517	1,523787816
Eif3b	0,397420225	-0,228421529
Calu	0,398716715	-0,24323527
Sdhc	0,398801027	0,55534935
Lgals9	0,398927518	0,262397766
Wdr82	0,399076627	-0,249689102

Table S2.

Psat1		0,399753433	1,630343755
Sumo2		0,399791826	0,301939011
Igkv2-137		0,399841534	-0,761645635
Mpi		0,399845515	-0,3164711
Fundc2		0,399850336	-0,37018458
Dnph1		0,399893896	-0,437385559
Actb		0,399897534	1,768511454
Eif5a		0,39990016	0,541852951
Vps18		0,399908134	1,110141754
Dpep1		0,399934741	-0,467614492
Pacs1		0,399946188	0,473606745
Vps25		0,39994757	0,314305623
Ndufb2		0,399956605	1,90845108
Mrpl19		0,399959027	0,502545675
Tpm4		0,399962832	0,396362305
Aldh1a3		0,400011538	-0,17033577
	Sep-11	0,400450255	-0,202879588
Actr3		0,400896815	0,203229268
Ei24		0,400991399	0,48211352
Exosc6		0,40104176	0,467375437
Sec24a		0,401119184	0,224902471
Mgat2		0,401155952	-0,332556407
Golga1		0,40124419	-0,496798197
Pctp		0,401643016	-0,260865529
Mgat1		0,402386523	0,432587306
Cox20		0,402412214	0,390349706
Ccs		0,402514467	-0,304697037
Nmt1		0,402547188	-0,338038762
Myd88		0,402578145	-0,267873764
Wapal		0,402607812	-0,394616445
Oat		0,403112381	-0,455459595
Shc1		0,403365482	0,293433507
Wipi1		0,403413892	0,760875702
Bola1		0,403450333	-0,807128906
Mtm1		0,403929002	0,55238533
Tspan15		0,403953075	-0,640207291
Sf3b6		0,404814697	-0,22238795
P4hb		0,404877493	-0,398755391
Rbm27		0,404937302	0,406195958
Med24		0,404942966	-1,143290838
Cox7a2l		0,407774684	0,676464717
Mvd		0,407965834	0,342189789
Ecsit		0,408239089	-0,308579127
Kdm2a		0,408543616	-0,397692998
Mpv17		0,408672779	0,790664673
Top2b		0,409070458	-0,149657567
Copb2		0,40947395	-0,212364833
	Sep-01	0,409567909	-0,866599401
Gstm2		0,410082071	-0,645172755
Serpina1e		0,410119281	0,861785253
Fam98a		0,410276341	0,251272837
Plekha7		0,410960302	0,901589076

Table S2.

Pqlc3	0,41108982	0,650632858
Cd81	0,411180328	-0,346413294
Gmppa	0,411251971	-0,296571096
Ppp1r12a	0,411449449	0,319089254
Hsp90ab1	0,411830028	-0,91775322
Actr10	0,411926952	0,313398361
Ndufa7	0,412173585	-0,375228882
Rcn1	0,412229003	-0,417771657
Lnpep	0,41226281	-0,200721741
Tpp2	0,412276904	-0,212591171
Trmt2a	0,412371743	-0,42601649
Pus7	0,412462312	0,905388514
Rdh14	0,412555451	-0,318018595
Slc35a4	0,412574481	0,524948756
Kiaa1522	0,412664156	0,576572418
Prkar2a	0,412928773	-0,349302292
Actn1	0,41433302	-1,219285965
Txlng	0,414398996	-0,140884399
Arfgap1	0,414486995	-0,237044652
Sod3	0,414511599	0,345253627
Agfg1	0,414726817	0,449441274
Mtpap	0,414912621	-1,266091665
App	0,415046009	-0,234960556
Tigar	0,415175955	1,107114156
Msra	0,415300375	0,369889577
Ttn	0,415600876	-0,700719833
Fam98b	0,415671044	-0,240730921
Itgb2	0,41580125	-0,279110591
Ca13	0,415931229	0,413525263
Asns	0,417015933	0,85534668
	0,41713554	-0,545424779
Rap1a	0,417893225	0,37367185
Nutf2	0,418223471	0,324767431
Gpr89a	0,418638378	0,768875758
Elavl1	0,418654613	0,210800171
	0,418714063	0,437018712
S100a10	0,4187199	-0,400634766
Atp6v1h	0,419794393	-0,310129166
Chid1	0,419894147	-0,395217896
Igkv6-13;Igk-V19-17	0,41992521	-0,974903107
Wdr11	0,419953908	0,989301682
Cyp2s1	0,419965142	-0,341199875
Rpl35a	0,420001245	0,500665029
Pex3	0,420041058	0,378342946
Serpina1d	0,422552239	-0,433909734
Cdc42	0,423664283	0,196659724
Comtd1	0,423891858	-0,338186264
Tbcel	0,424134203	-0,987423579
Erlin2	0,425160248	-0,225384394
Aktip	0,425204595	-0,953687668
Pelp1	0,426191186	-0,410961151
Aoc3	0,426607509	0,232106527

Table S2.

Tnpo2	0,426935484	-1,289178212
Mia3	0,427275659	-0,238311768
Mapre1	0,427425914	0,295574188
Gimap7	0,429933685	0,564884186
Ppp2r5e	0,430742255	0,377157847
Sfr1	0,430907402	-0,181383133
Aak1	0,430978644	-0,275613149
Gtpbp1	0,431077399	0,3375295
Phb2	0,431412129	0,254641215
H2-DMb2;H2-DMb1	0,432320445	-1,07215627
2210010C04Rik	0,432324057	1,068652471
Iglv1;Iglv2	0,432330139	-2,242471695
Prkab1	0,433245598	1,189933777
Lonp2	0,433250927	0,456126531
Cdc16	0,433361334	-0,58403333
BC017158	0,434874961	-0,442171097
Cdkl3	0,435012346	-1,107988993
Gapdh	0,435111385	0,500343959
Cdk2ap1;Cdk2ap2	0,435228871	-0,298999786
Rcc2	0,436948504	0,289543788
Med30	0,437406471	-0,900649389
Gm766	0,437499383	-0,383785248
Ppfibp1	0,437898953	-1,118992488
Lin7a	0,438158203	0,878181458
Gfm2	0,438184729	-0,270766576
Wdr43	0,438277795	0,365732193
Timm17a	0,439289846	0,230939229
Ggh	0,440068902	0,394726435
Hp	0,441056581	1,506397247
Nt5c	0,441821088	-0,293900808
Echdc2	0,441857406	0,716348012
Pfkfb4;Pfkfb1	0,441871582	-0,526673635
Gnb4	0,442058968	-0,393059413
Lmnb2	0,442425545	0,204229991
	0,44263229	0,601307551
Ermp1	0,442741945	-0,320034027
Igsf5;Pcp4	0,442886231	-0,74567922
Camsap3	0,443022086	0,42493693
Eef2k	0,443509503	-0,51127243
Pacsin2	0,445129022	-0,222700755
Tpp1	0,445723039	0,412412643
Ufl1	0,445876876	-0,417983373
Ggt1	0,445905695	-0,789393743
Dpm1	0,446170694	0,248294195
Gm3336	0,44619951	1,152997971
Rnase4	0,446252831	0,637399673
Mydgf	0,446265443	0,410044988
Pnpo	0,446403911	0,312261581
Hdac6	0,446432284	0,514866511
Gemin5	0,44646088	-0,436876933
Dhx15	0,447064142	-0,233110428
Ak3	0,448020763	-0,161066691

Table S2.

Akr1b10	0,448087912	-0,282057444
Nsun2	0,448257553	-0,185070674
Atp6v1f	0,448672361	0,36312294
Cyfip1;Cyfip2	0,44895029	-0,283021291
Hsd11b1	0,449162195	0,299423218
Rab7a	0,449294727	0,171468735
Poldip2	0,449469835	0,256410599
Hrg	0,449572952	1,140778859
Slc6a19	0,450056029	0,337697983
Pex14	0,450425571	-0,35012118
Eif3a	0,451319537	0,265963236
Mogat2	0,451383024	-0,258757909
Akt1	0,45263382	0,372530619
Ndufv2	0,452745515	0,324839274
L1cam	0,453380735	0,278181076
Ssh3	0,453387234	0,7883358
Camk2b	0,453648846	-0,35338974
Ddt	0,453802612	-0,582137426
Bpgm	0,453804493	-0,301151276
Kiaa2013	0,455694175	0,125275294
Renbp	0,455784046	0,909941355
Mcm2	0,455832473	-0,241921107
Agfg2	0,456141904	0,506214142
Tcirg1	0,456278872	-0,378125509
Pebp1	0,456385455	0,22889328
Nop56	0,456495607	-0,221083323
Dlg3	0,457599758	0,416788737
Sirt2	0,457700787	0,374468486
Clip1	0,458767927	-0,363145192
Pip	0,458845036	-1,622212092
Mrps2	0,459097399	0,715054194
Osbp	0,459153567	-0,205394745
Rpl30	0,45929241	-0,276437759
Ddx42	0,460614083	1,301659266
Irf3	0,460952266	0,41353035
Hcls1	0,461756569	0,352096558
Snrpa1	0,462318841	0,278120041
Bpifa2	0,463269544	-1,447580973
Bcs1l	0,464032589	-0,263270696
Nit2	0,464309732	0,288466771
Rasa1	0,464326703	-1,244713465
Asun	0,464332831	0,667570114
Tom1l1	0,464441496	-0,476675034
Rdh10	0,464566265	-0,388587316
Ciapin1	0,464581599	0,246822357
Lclat1	0,465405601	-0,407564799
Nudt12	0,465454545	-0,455058416
Nans	0,466716005	-0,262679418
Ptpn11	0,466856455	-0,195241928
Nudt16l1	0,46718797	-0,211971283
H2afy	0,46725436	0,206424713
Urah	0,467464983	0,422598521

Table S2.

Nmt2	0,467815182	-0,668217341
Hnf1a	0,467817363	0,598081589
Cds1	0,467853497	0,857039134
Zfr	0,467859544	-0,425668081
Ndufaf4	0,467889423	-0,28903389
Zadh2	0,467921922	0,24758021
Emilin1	0,468013197	-0,199693044
Sdf2l1	0,468397001	0,459023158
Mapre2	0,468647482	-0,44785436
Hprt1	0,46935451	0,245714823
1700037H04Rik	0,46942121	0,736716588
Eno3	0,469504642	-0,367266337
Srsf5	0,470738102	-0,239775976
Slc28a2;Gm14085	0,470746858	0,340195338
Mrpl27	0,470782036	0,447689692
Sars2	0,470842955	-0,334571203
Arf5	0,471017334	-0,311126709
Tcaf2	0,471057066	0,341019948
Mcm3	0,471117489	-0,275454203
Gale	0,47112201	0,449735641
Tgfbi	0,47221147	0,224793752
Tbrg4	0,473455957	0,341790517
Kdelr1;Kdelr2;Kdelr3	0,473629364	-0,605950673
Cygb	0,473770746	-0,375486374
Commd10	0,474229508	0,372020721
Scly	0,474339893	-0,368531545
Pex11b	0,474400239	-0,362223943
Atp5o	0,47446778	0,242221832
Cherp	0,474697259	0,280371348
Uso1	0,474903783	-0,182067871
Ca9;Car9	0,475249777	-0,936072032
Bst1	0,475360334	-0,650892893
Snrnp40	0,475865516	0,294686
Cutc	0,476007143	-0,434448242
Tfrc	0,476539994	-0,271729151
Ada	0,476582986	0,364198049
Tmem30b	0,476678954	-0,360249201
Sntb1	0,477544131	0,701172511
Ldha	0,477809863	0,199760437
Cldnd1	0,477959014	-1,275787354
Mpc1	0,478978925	-0,278661092
Gar1	0,478998812	-0,315034866
Gcn1l1	0,479246738	-0,171480179
Scfd2	0,479264967	-0,371271769
Qil1	0,479290208	0,341913223
Golga2	0,479305844	-0,199740092
Coro1c	0,479306256	0,236334483
Pcyt2	0,480867556	-0,241723378
Pbdc1	0,480981043	0,305855433
Mecp2	0,481297009	0,332583745
Csk	0,481469822	-0,400283178
Vcpip1	0,481581533	-0,389796575

Table S2.

Gm7075;Rnf7	0,481608991	-0,422021866
F2	0,481666075	0,930229823
Xpnpep1	0,481718002	-0,17277654
Ckap5	0,481860438	-0,186740239
Mesdc2	0,482885343	0,278191249
Tbca	0,483342984	-0,223597209
Tfip11	0,483896043	-0,359904607
Hnrnpk;Gm7964	0,484089755	-0,20837911
Tmed10	0,484403778	0,332926432
Gm10036;Rpl11	0,484920626	0,176439285
Ccdc134	0,485015929	0,279955546
Ighv1-81	0,485076968	-0,615528107
Tbc1d23	0,487711085	-0,563741048
Uqcrc2	0,488022392	-0,195982615
Slc9a3r2	0,488056587	0,411963781
Rbm14	0,488659794	-0,175106684
Grhpr	0,489261919	0,300899506
Ctbp1	0,489311746	-0,442443848
Il16	0,489312132	0,419027964
Prodh	0,489794645	-0,256802877
Avl9	0,489972361	-0,336743673
Rhoc	0,489985882	0,432734807
Cnot3	0,490085832	-0,210604986
Cdk18	0,491132785	-0,536357244
Ecm1	0,491250073	0,471426646
Tes	0,491471366	0,239110947
Tsta3	0,491874376	0,235835393
Slc22a18	0,491978861	-0,459346771
Ctif	0,492007042	-0,383244197
Enpp1	0,492204165	-0,283857981
Map7d1	0,492308504	0,3885053
Sdhb	0,492501906	0,222210566
Pcx;Pc	0,492994138	0,247048696
Hrsp12	0,49309581	0,957523982
Cycs;Gm10108	0,493490334	0,264844259
Gphn	0,494663543	-0,167159398
Ddx39b	0,494968119	-0,221529643
Lrrc1	0,495049737	-0,846175512
Mrpl28	0,495183626	-0,366347631
	0,495194615	0,295095444
Cox7c	0,495339578	-0,303372065
	0,495826951	0,362889608
Gnas	0,495909994	-0,257303238
Zfpl1	0,496028046	-0,260698954
Fabp5	0,496478972	0,509791692
Ighm	0,496615474	0,358008703
Mrvi1	0,496855309	-0,306241353
Gipc1	0,497000292	0,583847046
Phgdh	0,497013707	0,834269206
Uqcrc1	0,497145359	0,19077301
Nudcd1	0,497519091	1,101846695
Csnk2a2	0,49762449	-0,238188426

Table S2.

Alg2	0,498016895	0,497067134
Fbxo22	0,498097902	-0,280380249
Trappc5	0,498112988	-0,201721827
Trappc3	0,498283552	-0,284946442
Atpaf2	0,49837369	-0,304002126
Pet100	0,499197208	-0,285909017
Tm9sf3	0,499249345	-0,278441747
Rab25	0,499255598	0,33078893
Retsat	0,499877907	-0,402711868
Sardh	0,500305725	1,645056407
Cops7a	0,500482278	1,363150915
Prpf31	0,500602787	-0,218781789
Egfr	0,500612257	0,272743225
Necap2	0,500617707	0,293680191
Tuba1a;Tuba3a	0,501536854	0,440869649
Pnliprp1	0,502273281	1,121214549
Ankrd28	0,502276602	-0,334990184
Pls3	0,502307425	-0,189141591
Atp6v1a	0,502683662	-0,231428782
Coq7	0,502740075	-0,371116002
Tex10	0,50274087	0,423133214
Arhgef7	0,503103388	0,19832929
Larp4	0,503798553	-0,228020986
Aimp2	0,503865663	0,209306717
Mon1a	0,504097222	0,83616066
Gnl3	0,505000868	-0,302305857
9030624J02Rik	0,505355697	0,396425247
Cacna2d1	0,507624169	-0,395254135
Atp5f1	0,508315607	-0,165385564
Ksr1	0,508364057	-0,977561315
Iars	0,508476025	-0,282332102
Frmd8	0,509073283	0,565087001
Edc4	0,509140878	-0,184147517
Fkbp5	0,509154401	-0,260181427
Gspt1	0,509287901	-0,209735235
Fam107b	0,509599077	0,444765091
Ttll12	0,510858461	-0,256198247
Prpf38b	0,510904268	-0,176681519
Nars	0,511453602	0,271544774
Bcat2	0,511703832	0,819696426
Hk2	0,511746544	-0,188023249
Clptm1	0,513145983	0,295618057
Rmdn3	0,51327733	-0,182617188
Gnpnat1	0,51336518	0,298274358
Clec3b	0,513487622	0,219486872
Hnrnph1	0,513686511	-0,142737071
Gpt	0,513820587	-0,24587822
Lmo7	0,514490371	0,274012248
Spcs3	0,514572824	0,377768834
Mri1	0,514590805	0,235603333
Odr4;BC003331	0,51534865	0,517477671
Stk38	0,515908125	-0,207382838

Table S2.

Erp27	0,516005741	2,439297994
Nup188	0,516215782	-0,474246343
Stt3a	0,51641411	0,334780375
	0,516415376	1,067985535
Atxn3	0,516771789	-0,458895365
Sil1	0,517404414	0,245586395
Gart	0,517410484	-0,14135615
Dnajc8	0,51749341	0,203748067
Aldh2	0,518372745	-0,256170909
Dhx32	0,518438717	0,611855189
Ppap2b	0,518439611	-0,327243805
Lpcat2	0,518723891	-0,383043289
Ndufb5	0,519065217	-0,150066376
Rangap1	0,519653417	0,175919215
Gng12	0,519732419	-0,203922908
Pigg	0,520561303	-0,253341675
Htra2	0,521748571	0,991465251
Mlec	0,523098543	0,231323878
Eya3	0,523700828	-0,328422546
Mrps25	0,523716733	-0,26114591
Llgl2	0,523805936	0,22140185
Ighv1-76	0,523824401	0,557415009
Bak1	0,523944112	-0,188971837
Hnrnpc	0,524061609	-0,19124349
Zak	0,524123252	0,258447647
Rasip1	0,524303048	0,458147049
Mief1	0,524323739	0,93680954
Arl1	0,524352137	-0,503447851
Opa1	0,524508967	0,185248693
Aimp1	0,524596811	-0,201244354
Rrm2	0,525361411	0,480868022
Tor1aip1	0,525741404	0,165790558
Sdf2	0,525746303	0,259910583
Mecr	0,525758317	-0,201843262
Sec13	0,52581353	-0,210005442
Nup155	0,525857183	-0,180024465
Antxr2	0,526694295	-0,519858042
Ndufb9	0,526713231	0,160202026
Kin	0,526831014	0,751804352
Sec63	0,526948864	-0,242293676
Armc8	0,527832009	-0,229338328
Adck4	0,528167943	-0,450318019
Fam129a	0,528250709	0,460041682
Gatad2b	0,528371988	-0,230107625
Mtif2	0,52926644	-0,286109924
Bre	0,529526778	0,699172974
Mtco2	0,529684509	0,352705638
Itgb4	0,529737259	0,180510839
Fbln1	0,529745042	0,225777944
Abhd11	0,529803056	0,313053131
Lancl2	0,529869307	-0,244648616
Ptpn1	0,529879423	-1,055555979

Table S2.

Iscu		0,529942308	-0,19813029
Fuom		0,53054566	-0,229005178
Dmd		0,531972874	0,153118134
Trim26		0,532123233	-0,257527669
	Sep-09	0,533048829	0,358904521
Narfl		0,533071711	0,276375453
Prep		0,533150282	-0,189291
Set		0,533165772	0,359439214
Dym		0,533387479	-0,407929103
Pitpna		0,53344921	-0,170871735
Cyb5r1		0,533537941	1,078879674
Pwp1		0,533632929	-0,165496826
Cfh		0,533674183	0,43046697
Thy1		0,534531417	-0,343574524
Ctrl		0,535291467	0,978830973
Hacd3		0,53532507	-0,233920415
Atp6ap1		0,535468468	-0,331782659
Wtap		0,536108078	-0,551833471
Pck2		0,536348805	0,384498596
Chmp6		0,536398424	-0,383544922
Tstd1		0,536937869	0,443335215
Farsa		0,537021372	-0,202607473
Farp1		0,537162124	-0,217502594
Ankfy1		0,537245644	0,126676559
Psmb3		0,537822572	-0,264246623
Phyh		0,537912946	-0,527332942
Pon3		0,537937079	0,160016378
Psmg1		0,538071288	-0,165772756
Vma21		0,539790123	-0,168787638
Lad1		0,540030286	0,346988042
Nnt		0,54009425	1,20874087
Elovl6		0,540472962	0,35881424
rp9		0,540572549	-0,467664719
Mob2		0,540573031	0,229386648
Mbnl1		0,540573991	-0,24221611
Papss1		0,540590311	0,278526306
Sucla2		0,542927212	-0,19963328
Pum2		0,543006997	-0,369972865
Mon1b		0,544440963	-0,236054103
Galnt7		0,544523636	-0,217685699
Surf1		0,545644295	0,248245239
Scamp3		0,54610232	-0,33808581
Eef1a1		0,548347098	0,174404144
Fam83b		0,548462183	0,296090444
Polr2c		0,548494696	-0,333838781
Rps16		0,548506428	0,21373113
Atp6v0a1		0,548600279	-0,236499786
Ccdc93		0,548601227	-0,365527471
Cldn3		0,548603122	0,291240692
Cops7b		0,548647864	0,83653005
Trappc11		0,548649162	-0,176938375
Hspa4		0,548669083	-0,145807902

Table S2.

Tinag	0,548685108	-0,251805623
Fblim1	0,548818258	0,47717158
Ngly1	0,548860802	0,356608073
P2rx4	0,548976045	0,431471507
Gpr180	0,549013645	-0,920963923
Actn1	0,549075125	0,201972961
	0,549129005	-0,538473129
Myo18a	0,550191933	0,148681005
Scpep1	0,550385321	0,268940608
Epcam	0,550390434	0,332129161
Uap1l1	0,550707744	-0,283220291
Hnrnpf	0,550732574	-0,116072337
Dcxr	0,550757433	0,333238602
Eef1g	0,550802887	0,219394684
Rab8b	0,55080378	-0,224632263
Anapc1	0,550854444	-0,375785828
Abcb7	0,551353121	-0,25544548
Smu1	0,551466741	0,275749842
Map1b	0,551477248	-0,294733683
Mrpl55	0,551484336	0,426198959
Timm8a1	0,551505269	0,274816513
Acadl	0,55167304	-0,235720317
Sec62	0,552990582	0,388376236
Wdfy1	0,553896428	-0,273490906
Mocs2	0,554674419	-0,353516261
Pdk3	0,554835177	-0,242026011
Oas1g;Oas1a	0,554893499	0,1762441
Fdxr	0,554915329	-0,251324336
Snd1	0,554922864	0,446987152
Eif1;Eif1b	0,554997236	0,156276067
Hscb	0,555068918	-0,287087123
Nbas	0,555285991	-0,277432124
Ufc1	0,555411212	-0,288478216
Eif2s2	0,555498343	0,256955465
Cct3	0,5556455	-0,126411438
Tspan13	0,55642727	0,214901606
Yif1b	0,556533113	-0,439161301
Gtf2f2	0,556814345	0,162824631
Rnh1	0,556895753	0,207584381
Ighv5-15;Ighv5-12	0,556981527	-0,994204203
Cd34	0,557644983	-0,199292501
Cetn2	0,557739322	-0,226844152
Pea15;Pea15a	0,558540347	0,393421173
Pdcd5	0,558665565	0,188634237
Srp54;Srp54c	0,559162996	-0,33391571
Sco2	0,559311863	-0,226483663
Ddx27	0,559531224	-0,383913676
Gm21970;Il10rb	0,559685195	-0,263799032
Tpt1	0,559709651	-0,15319252
Prdx2	0,559713971	0,133666992
Rras	0,560708081	0,179833094
Numa1	0,561086013	-0,172122955

Table S2.

Casz1	0,56162373	-0,273819606
Marcksl1	0,561647253	0,353116989
Lims2	0,561771068	-0,260624568
Clps	0,561837452	0,787939072
Ubac1	0,563106476	-0,730862935
Mark3;Mark1	0,563293472	0,17415301
Edf1	0,563448011	0,312366486
Eif3m	0,564025226	0,308970769
Lsr	0,564808114	0,962148031
Pgrmc2	0,565656344	-0,208094915
	0,566543562	0,50855573
Uba3	0,567688013	-0,314078013
Dguok	0,567784287	0,633820852
Pofut1	0,567903588	-0,320386887
Mdp1	0,567939759	-0,095352173
Vrk1	0,568182564	0,215025584
Ndufs1	0,56833789	0,123901367
Akap8	0,568437517	0,272228241
Adam10	0,568535011	-0,245742798
Phf5a	0,568571272	0,295017242
Kras	0,569198907	-0,099529266
Igkv17-127;Igkv17-121	0,56953947	-0,808781306
Rhot2	0,569894047	0,247555415
Mtif3	0,570330786	0,348930359
Ywhah	0,570383629	-0,187074025
Snrpa	0,570486486	0,248343786
Ipo9	0,571875614	-0,231261571
Ufsp2	0,57241778	-0,342674255
Mrpl13	0,572737186	-0,315054576
Gfm1	0,573161079	-0,121586482
Ap1g2	0,57333842	-0,291154226
Gm27029;Aarsd1	0,573451376	-0,123760223
Hnrnpd	0,574592593	0,217372259
Serpina6	0,575348938	-0,409378052
Pam16	0,575357473	0,328452428
Lgals8	0,575978231	-0,357843399
Gorasp2	0,576932535	0,345208486
Eps15	0,57715529	-0,111544927
Slc3a2	0,578362599	-0,209421794
Pxmp4	0,578519848	-0,43927002
Lypla1	0,579666757	0,27498881
Gsk3a	0,579710334	-0,261322021
Tmem254b;Tmem254	0,579722041	-0,443962097
Podxl	0,579733913	-0,311505
Mrps5	0,579867607	-0,246353785
Rpl5	0,579891363	0,187711716
Hkdc1	0,5799	-0,241597493
Birc6	0,579903287	-0,18473053
Enpp3	0,58005423	-0,333608627
Rbms3;Rbms1	0,580259149	0,417753855
Uchl5	0,580335861	0,310891469
Muc2	0,58037605	0,222124736

Table S2.

Copg1		0,580385908	-0,170196533
Ddx1		0,580719198	-0,132740657
Arpc4		0,58116405	-0,20761172
Cela3b		0,581434289	0,892029444
Slco2a1		0,581456316	-0,296319326
Cnot7		0,581494589	0,777851105
Kpna3		0,581514479	0,386401494
Psma2		0,582806164	0,124015172
Sptan1		0,583620541	-0,118631999
Plin4		0,583647758	-0,141949972
Yrdc		0,583771954	-0,568049749
Atxn10		0,584373751	-0,356920242
Cab39l		0,584412527	0,720916748
Anp32a		0,586466937	-0,165981929
	Sep-15	0,586829242	0,141075134
Fam49a		0,586900162	0,338784536
Ola1		0,586950378	-0,12586085
Tceb1		0,587433962	0,215970357
Osbpl9		0,587592343	0,600814184
Ptgfrn		0,589076716	0,180250804
Immt		0,589111948	-0,118648529
Nomo1		0,5892311	0,167427063
Flad1		0,589235326	-0,400866191
Nat2		0,5892432	-0,295742035
Rela		0,589289871	0,402523041
Rbm47		0,589331178	-0,196406047
Hspa1a		0,589743011	0,171162923
Ptdss1		0,589784467	-0,302958806
Blvra		0,58984028	-0,159193675
Tor1aip2		0,589852609	0,167844137
Plekha5		0,590162278	0,573602041
Sdcbp		0,590598979	-0,242270152
Tm9sf4		0,591567132	-0,21624438
Ik		0,592115973	0,170866013
Mocs3		0,593928073	0,850441615
Lrrc19		0,594482426	-0,51383845
Anxa2		0,594717811	0,127581278
Ktn1		0,595231966	0,172198613
Cbfb		0,596030027	0,284105937
Pla2g16		0,597320118	-0,275973638
Arsb		0,59744373	0,437362035
Trim14		0,597451622	0,269408544
Cox7a1		0,597710308	0,212899526
Atic		0,59772255	0,262667974
Exosc2		0,5977308	0,881070455
Sf3a3		0,597827715	-0,175153732
Aldh7a1		0,597862955	0,715874354
Ndufv1		0,598006954	0,180351257
Kbtbd11		0,598158781	-0,282758713
Atl3		0,598294118	-0,134284973
Tomm6		0,598324339	0,168457667
Fryl		0,598428648	-0,264772415

Table S2.

Dhdh	0,599035514	-0,294413249
Mtx1	0,599102564	0,164519628
Cul2	0,599451148	-0,157145182
Blvrb	0,599885836	-0,390753428
	0,599922071	-0,798914591
Dido1	0,599932764	-0,197134018
Ubfd1	0,600180267	-0,225692749
Cdx2	0,600360341	0,429998398
Lias	0,600394561	0,258335749
Vta1	0,601985083	0,215398788
Nfkb2	0,602048495	0,662285487
Trappc9	0,603587124	0,233968099
Usp8	0,603616511	0,385192235
D10Jhu81e	0,603625333	0,153208415
Vps45	0,603748669	0,802303314
Slc12a2	0,603758318	0,213331858
Mrpl3	0,603943647	-0,242848078
Herpud1	0,604031915	0,418100357
Mbd2	0,604043605	0,188158671
Itga2	0,604127558	-0,304072062
Mccc1	0,604179596	-0,292362849
Tjp3	0,60421992	-0,180476507
Exosc7	0,604398301	-0,160479228
Sncg	0,60460828	0,211444219
Cpt2	0,604708256	-0,125923157
Lman1	0,605188644	0,388484319
Mrpl32	0,605371489	0,481126785
Clec2h	0,605531937	0,259085973
Nudt21	0,60554825	0,191591263
Pisd;Gm20671	0,605708831	-0,228413264
Ca4;Car4	0,605839788	0,158268611
Eif3g	0,606229404	0,211644491
Psen1;Psen2	0,606998147	0,935774485
Defa22;Defa21	0,607038095	0,407765706
Farp2	0,607040465	-0,167994181
Mpc2	0,607060381	0,262676875
Eef1e1	0,607090524	-0,188442866
Map2	0,60719873	-0,220088959
Pdia2	0,607586462	1,518774033
Stxbp1	0,608372191	-0,200806936
Hdhd2	0,608527205	-0,171413422
Wdr37	0,608562368	-0,704393387
Qars	0,608660502	-0,135787328
Xpo1	0,609409031	-0,136327744
Chuk	0,609846885	0,530289968
Hsd17b10	0,610272751	-0,17031161
Tmem165	0,610346174	0,340937932
Urgcp	0,610417524	0,444810867
Hnrnpul1	0,610421719	-0,230840683
Dpp7	0,610457806	0,298231125
Pdk1	0,610529644	0,795936584
Mrpl41;Gm6434	0,610582652	0,18626531

Table S2.

Cgref1	0,610651212	-0,385279338
Prkg1	0,610768501	-0,24415652
Gm1123	0,611198526	-0,230656942
Hmgcs2	0,612200053	-0,18506813
Rbx1	0,614370526	0,203049978
Diap2;Diaph2	0,615764273	-0,379182816
Rbpms	0,616719621	0,546101888
Farsb	0,616871943	0,141444524
Glyr1	0,617453207	0,187555949
Uba2	0,618864652	-0,1457208
FAM120A	0,620531792	0,129402796
Entpd1	0,620790123	-0,213912964
Ctnna2	0,620819732	-1,59112676
Sun2	0,620828782	-0,292444229
Cltb	0,620849567	0,196655909
Rdx	0,620976915	0,191445033
Ist1	0,620982677	-0,160533269
Sf3a2	0,622232363	0,41607221
Rala	0,622320755	-0,233868281
Spink3	0,622483879	-0,856323878
Strip1;Strip2	0,62264709	0,227229436
Hspbp1	0,624206445	-0,249998093
Iah1	0,62527187	-0,221129735
Epb4.1;Epb41	0,625426551	0,691083272
Ran;1700009N14Rik	0,625496207	0,212284088
Eif3k	0,625502092	-0,15547816
Etf1	0,625522513	-0,14381663
Tmem106a	0,625636839	-0,281630198
Nde1;Ndel1	0,625800576	-0,65176328
Tamm41	0,627187451	0,261285782
Ptprk	0,627843219	-0,14558665
Hspa12a	0,627945635	-0,222024918
Pnpla2	0,627960303	-0,565928141
Scgb2b20;Scgb2b7	0,628027168	-0,858594259
Stom	0,628159791	-0,150265376
Rad50	0,629002349	-0,245654424
Snap29	0,629045656	-0,280977885
Dnm1l	0,62908977	-0,116031647
Rnf126	0,629152843	0,657460531
Dnaja3	0,629207508	-0,17971166
Aprt	0,629344459	-0,248058955
Scgb1b20;Abpa29_a7;Scgb1b3	0,629998436	0,678407669
Idh2	0,630872917	0,161963781
Try5	0,630880959	0,651139577
Ndufs8	0,631045336	0,17183876
Rbm39	0,631587715	-0,158368429
Zfand6	0,631700312	0,708979925
Uggt1	0,631725215	0,166301092
Rmdn1	0,631752148	-0,224752426
Cr1l	0,633187419	-0,15496254
Aqp11	0,633234529	-0,386834462
Hspa2	0,633260858	0,166266759

Table S2.

Inpp1	0,634356976	-0,218782425
Deptor	0,63445738	0,325006485
Tmed9	0,635301818	-0,314553579
Serinc3	0,636186964	-0,318609238
H2afv;H2afz	0,636694704	0,253873189
Abhd3	0,636865819	0,426241557
Cmtm7	0,637529442	0,244915644
Inppl1	0,6376274	-0,191256841
Cox15	0,638852697	-0,252417882
Eprs	0,639231527	0,146886826
Ppp1r7	0,639861068	-0,174694061
Serpinb1a	0,640031096	-0,170772552
Araf	0,64053057	-0,300396601
Usp15	0,640589332	0,130938212
Cpb1	0,640631961	0,785859426
Try4	0,640741393	0,606215795
Ostf1	0,640755694	0,135774612
Trim47	0,64173868	0,469551722
Gne	0,641986036	0,132092794
Larp1	0,642120021	0,229022344
Cav2	0,642436401	0,234790802
Pdcl3	0,642647059	-0,146855672
Vps29	0,642723184	-0,229746501
Cps1	0,642736951	-0,153539022
Uqcr10	0,642812903	-0,224650065
Rtn4ip1	0,64283441	1,025042216
Nedd4l	0,642866288	-0,342741648
Acadsb	0,643005422	0,152133306
Rps19	0,643126033	0,187357585
Prune	0,643262887	0,1710186
Lsm14a	0,643275071	0,137106578
Gnao1	0,643373904	-0,271930695
2200002D01Rik	0,643504772	0,415090561
Ssfa2	0,644006182	0,201114655
Hbbt1;Hbb-bs	0,644117495	-0,454817454
Cpa2	0,645117693	0,690178553
Cog8	0,645487127	0,291737239
Map4	0,646017499	0,173712413
Ncor1	0,646153925	-0,261372248
Psmc1	0,646558271	0,196813583
Taf15	0,646900771	0,331713994
Camk1d	0,646981239	-0,23072052
Ptrf	0,646990741	0,113810857
Aqr	0,64702261	-0,425746918
Amy2;Amy2a1	0,647067112	0,689872742
Actbl2	0,647553159	0,180232366
Pof1b	0,647578731	0,22392718
Cnep1r1	0,647812067	0,172486623
Ilkap	0,648030785	0,350497564
Plp2	0,648068789	-0,21397845
Eif2s3x	0,648120092	0,15073204
Cd47	0,648661708	0,248490651

Table S2.

Sdhaf2		0,648958974	0,407126109
Psmd14		0,64970828	-0,146575292
Coro7		0,649885187	0,127325058
Kif2a		0,650579257	-0,221330007
Reep5		0,650626697	0,486486435
Mcm7		0,650663934	0,193929036
Ppp2r5a		0,651107015	-0,223526001
Thumpd3		0,65155055	-0,33257548
Grpel1		0,652219038	-0,123331706
	Mar-02	0,652256843	-0,193873088
Dnmt1		0,652346803	0,21993955
Bdh1		0,653461519	0,118808111
L7rn6;l7Rn6		0,653613497	0,772731145
Agpat2		0,65404903	-0,159489314
Ascc3		0,654154868	0,721322378
Iigp1		0,654185999	-0,594153722
Srm		0,654216092	0,293463389
Eps8l2		0,654259316	0,183840434
Otulin		0,654271126	-0,460029602
Clca4b;Clca4a		0,6550222	-0,231946945
Gc		0,655401478	-0,280797323
Nfib		0,655423763	-0,204983393
Magohb;Magoh		0,655590921	0,100406647
Edem3		0,655657143	-0,275615056
Serpinb9		0,655942915	-0,109595617
Nipsnap3b		0,656299542	-0,289168676
Rps3		0,656353631	0,144004822
C4b		0,657152024	0,383754094
Prkacb		0,658139511	-0,269622167
Tpm3		0,658563502	0,556062698
Commd9		0,659377099	-0,228547414
Psat1		0,659378433	0,71224912
Tmem186		0,659405186	-0,347771327
Slc39a4		0,659461245	0,395018895
Ogn		0,659511823	-0,232177734
Nmi		0,659546171	-0,182157516
Sestd1		0,660890808	0,180456161
Ruvbl1		0,660975362	-0,146385829
Trappc6b		0,661036585	-0,157524109
Mtor		0,661178979	-0,192254384
Pkm		0,66148731	-0,11112086
Sepsecs		0,661541741	-0,137475967
Dynlt1		0,661795587	-0,185529709
Pold2		0,66184069	-0,233984629
Atg7		0,662706897	0,202102025
Rplp2		0,662982003	0,173740387
Ndufs2		0,663363567	-0,080469131
Rpl12		0,663373543	0,198523204
Ptbp1		0,663400203	0,199513753
Tbc1d5		0,663753986	0,182097753
Serpinf1		0,663797417	-0,706660589
Mcts1		0,663798481	0,147322973

Table S2.

Nemf	0,66504427	-0,19891421
Clic1	0,665075911	0,132018407
Eif2b2	0,665749115	0,291962306
Chchd4	0,667179772	0,189338684
Lpgat1	0,669150657	-0,151151021
	0,669173616	0,397980372
Bicd2	0,67014957	-0,483226776
Vps52	0,670285138	-0,260418574
Bco2	0,670338384	-0,353249232
Letmd1	0,670367264	-0,352173487
Scrib	0,670376577	-0,107538859
Fam96b	0,67042464	-0,136152903
Eif3h	0,672900101	-0,146368663
Ndc1	0,673493064	-0,255071004
Rprd2	0,674826021	0,542262395
Aspa	0,67509453	0,298057556
Crip2	0,675402519	0,171961466
Scfd1	0,675420509	-0,205874761
Fahd2	0,675458669	-0,220519384
Agpat3	0,675953664	0,238541285
Hells	0,676320161	0,287434896
Eif2b1	0,676406848	0,184122086
Tagln2	0,679636638	-0,116784414
Ralb	0,681000754	0,139055888
Mrps27	0,681148893	-0,131808599
Hspa5	0,681187421	0,217834473
Sh3glb1	0,6815636	-0,250804901
Atp6v1d	0,682189495	0,111860911
Mrpl42	0,682298492	0,33848381
Bag4	0,682528378	-0,295501709
Ergic2	0,682668676	0,181952794
Isoc2a	0,682688755	-0,345106761
Gapdh;Gm7293	0,682830615	0,102661133
Gmds	0,682860156	-0,164000829
Acsl4	0,683524335	-0,433218002
Carm1	0,684656132	-0,263572693
Prdx4	0,685319629	-0,242822011
Suz12	0,685462387	0,179875692
Prss3;Gm10334	0,68642807	0,76218605
Ndufa13	0,686914372	-0,140775681
	0,687032307	-0,225687027
Glt28d2;Alg13	0,687061122	0,408735275
Dnaja1	0,687107554	0,121033351
Rps12	0,687112112	-0,223043442
Mpdu1	0,687154366	-0,26182429
Itih5	0,687173967	0,177206675
Snx6	0,687233275	-0,101399104
Akr1c13	0,687489872	0,180413564
Gzma	0,687652	-0,201313655
Vps4a	0,688077942	0,230626424
Prmt1	0,688085979	0,149157842
Hpgd	0,68822089	-0,221411387

Table S2.

Celf2	0,688246753	0,205786387
Eif2b5	0,688962797	0,242675145
Rbm5	0,689113659	0,275004069
Ntpcr	0,689215177	0,329163233
Adrbk1;Adrbk2	0,689238903	-0,167441686
Pfkfb2	0,689259536	-0,166980108
Ube2l3	0,689275767	0,095258077
Rragc;Rragd	0,689283113	-0,209089279
Fam210a	0,689308747	0,111310323
Mob4	0,68939521	0,510439555
Stag1	0,689449564	-0,513538361
Arhgap12	0,689508719	-0,322256724
Dnajc3	0,689583665	0,253413518
Tars	0,689602191	0,102535248
Helz2	0,689925889	-0,449475606
Srrt	0,690004975	-0,109086355
Irgq	0,69002887	0,354628881
Cmtr1	0,69011993	-0,34242185
Ghrl	0,690154152	0,261730194
Ilf3	0,690637832	0,159666697
Rab1	0,691301193	0,123402913
Etfdh	0,691384845	-0,167320887
Myo1c	0,691398063	-0,08876737
Smarca5	0,691547938	-0,08515803
Hist1h3b	0,691896723	0,164479574
Ncstn	0,692517122	-0,145404816
Pafah1b2	0,692537106	0,525455475
Eif5	0,69390377	0,128046672
Lonp1	0,693912181	0,10051918
Cmbl	0,694037661	0,234596888
Eif2b4	0,694135383	-0,160078049
Kti12	0,694183891	0,349411011
Prkar2b	0,69423996	-0,17520841
Ccdc25	0,694339361	-0,18656985
Stk25	0,694888559	0,329968135
Sdhd	0,694902699	-0,421738942
Eml4	0,695707076	0,112922668
Mrpl9	0,695747712	0,101596832
Mycbp	0,695791089	0,121177673
Col14a1	0,695850532	0,105369568
Abcb11	0,696434224	-1,165480932
Fam32a	0,696643758	0,471188863
G3bp2	0,697303015	0,174900055
Higd1a	0,697814678	0,386095683
Abce1	0,69843083	0,123185476
Sf3b3	0,69877303	0,078613917
Uqcc1	0,699274074	-0,269756317
Trim28	0,6994431	-0,086290995
Mbd3	0,699901283	-0,242345174
Hpca	0,700682782	-0,251502991
Ccar2	0,700728349	-0,097869237
Cmtm6	0,701438501	0,474307378

Table S2.

Atp1a4	0,701480276	-0,283309937
Crocc	0,701601973	0,109761556
Sphk2	0,701673479	-0,194337845
Rxrg;Rxrb;Rxra	0,701714286	-0,212907155
Slc16a3	0,70175117	-0,2300752
Yipf5	0,701759487	0,161531448
Coq9	0,70216839	-0,13381958
Dnajc16	0,702648289	0,390514374
Pgls	0,706653543	0,130110423
Galt	0,706771956	0,868728002
Tbc1d22a	0,708848008	0,171925227
Camk1	0,709134005	-0,539230982
Eif1a;Gm5662;Gm8300;Gm2016;Gm219	0,709437561	0,232456207
Skiv2l	0,709546326	0,429709752
Ssrp1	0,709867322	0,120447159
Yy1	0,710504544	0,149714788
Mt-Cyb	0,710511788	-0,306999207
Vasp	0,710927906	-0,223928452
Parl	0,71101203	-0,151680628
Sap30bp	0,71104832	-0,161659241
Eif2b3	0,711163886	-0,192733129
Dbnl	0,711338405	0,143849691
Hagh	0,71135297	-0,177384059
Parn	0,713288235	0,924811045
Qsox2	0,713408188	0,384452184
Surf6	0,713754472	0,102488836
Atp2b4	0,714099437	0,093094508
Impa1	0,71411269	-0,07458051
Plvap	0,714565281	0,134501139
Rars2	0,714574713	-0,206242243
Src	0,714641546	-0,103245417
Mavs	0,714749511	0,201354345
Sigmar1	0,714786099	-0,298131307
Atg2a	0,714786781	0,317625682
Tpm1	0,714850147	0,256328583
Nipsnap1	0,715251405	-0,161144892
Prpf3	0,71526784	0,255758921
Stard10	0,715364459	0,145123164
Fcho2	0,71622765	-0,216862361
Nags	0,716404396	-0,484648387
Ndufb6	0,717321289	-0,166190465
Rab32	0,717458628	-0,12374115
Cltc	0,71747353	-0,088678996
Pigk	0,717486579	-0,190409978
Stat5a;Stat5b	0,717654634	-0,144845327
Mal2	0,717956596	-0,194396973
Vps26a	0,718283764	0,094889323
Wnk1	0,718724835	-0,193321228
Tmem70	0,718964442	-0,760023117
Rps18;Gm10260	0,719058193	-0,170834859
Rwdd1	0,719112302	-0,187877019
Lactb	0,719154971	-0,199572881

Table S2.

Dpp8	0,720749757	-0,191894531
Rufy1	0,721556204	0,098836899
Dtymk	0,721592602	0,20153745
Prkar1a	0,72298127	0,106285731
Slc9a1	0,723047156	-0,153689702
Coa7	0,723091661	0,304271062
Ube2g1	0,723107977	0,1809419
Cyth3;Cyth2;Cyth1;Cyth4	0,723312758	0,178950628
Ighv9-2;Ighv9-4;Ighv9-3	0,723484937	0,209699631
Emc1	0,723992227	0,110096614
Bckdk	0,724292304	-0,250555038
Rnaseh2a	0,724445847	-0,164944331
Pdlim5	0,725153398	0,173006694
Eny2	0,725605436	0,145352681
Taco1	0,725615721	-0,138442357
Brd4	0,725816153	0,234495163
Ppp4r1	0,725903007	-0,137663523
Lrrc59	0,726231273	-0,196418762
Arfgef2	0,726480988	-0,134469986
Pkn1	0,726619821	0,167648951
Ifi30	0,726656977	0,153474172
Tpd52l1	0,726665051	0,330458323
Ubxn7	0,727597094	0,198129654
Marcks	0,727718364	0,158934275
Rad21	0,727732752	0,186660767
Dock6	0,727873185	0,293495814
Ubac2	0,728134429	0,185390472
Pnkp	0,728235123	0,568265915
Sgce	0,728283434	0,234891256
Ube3c	0,729011827	-0,132119497
Ppm1b	0,729028489	0,108513514
Bin1	0,729088626	-0,209649404
Gm7356	0,729122705	-0,922700246
Gm10250;Atp5h	0,729138031	-0,104612986
Qdpr	0,729188693	-0,180537542
Lgals1	0,729283712	0,218999227
Igkv1-110;Igkv1-99;Igkv1-115	0,729306261	-0,172307332
Ubxn4	0,7293462	0,093866984
Papln	0,729553304	0,421039581
Nrbp1	0,729931999	0,68929863
Setd7	0,730167792	-0,198212941
Igj	0,730591807	-0,238569895
Rtf1	0,730642564	-0,237826029
Nhlrc2	0,732185016	-0,115586599
Cpd	0,732338069	0,086790721
Txn	0,732405588	-0,101685842
Plcd1	0,733006259	-0,22763443
Mogs	0,735121059	-0,175443649
Rpl34	0,735415784	-0,108015696
Upf1	0,738092855	-0,089408239
Glul	0,738681097	-0,201265971
Epb4.1l4b;Epb41l4b	0,739636713	-0,346607208

Table S2.

Anxa6		0,739693195	0,092992147
C1qbp		0,739754808	0,222302755
Arhgef26		0,73978659	0,178430557
Tvp23b		0,740855153	-0,127188365
Dctn6		0,740900096	-0,230256399
Ndufs7		0,740970715	0,08460935
Tst		0,741006959	0,633543015
Ppp4r2		0,741073709	-0,117186228
Hars		0,741582534	-0,132858276
Hdlbp		0,743877189	0,208240509
Use1		0,744164988	0,315054576
Fnbp4		0,744395973	-0,341359456
9530053A07Rik		0,744547591	-1,00446256
Acad10		0,744993771	-0,142794291
Erc1;Erc2		0,745085071	-0,161565781
Gvin1		0,746013892	0,126728058
	Sep-09	0,746313218	0,140369415
Rab11b		0,746665071	-0,108469009
Ergic3		0,746774749	0,146253586
Lypla2		0,746874641	0,176980337
Chkb		0,746880651	0,146287918
Atp2a2		0,747028476	0,063035329
Spcs1		0,747085687	-0,339958827
Ighv6-3;Ighv6-7;Ighv6-6		0,747318519	-0,25470988
Myl6		0,747355486	0,113615672
Cybb		0,747373805	0,143015544
Basp1		0,747619775	-0,068941752
Sgk2		0,747662685	-0,44255956
Elovl1		0,747787011	0,559417725
Armc10		0,748106947	0,23910586
S100a11		0,748334129	-0,078173955
		0,748419847	-1,185801824
Bcam		0,748502028	0,306366603
Rabgef1		0,749731173	-0,096324285
Tjp2		0,749750656	-0,085555394
Adprh		0,74987506	-0,158789953
Thap4		0,749904126	0,332932154
	Mar-05	0,751649273	-0,182530721
Sgta		0,753988566	0,073980331
Sp110		0,754987149	0,390570958
Tnfaip8		0,755090693	-0,201649984
Polr2a		0,755191429	-0,082413356
Trappc6a		0,755205525	-0,100709915
Galnt3		0,755273073	-0,184342702
Cept1		0,755320485	-0,107717514
Col4a5		0,75544352	-0,372872035
Creld2		0,755445554	0,175306956
Dynlrb1		0,755959116	0,13033549
Pde5a		0,756625475	-0,093538284
Itpr2		0,756784816	-0,48084259
Dnah7c;Dnah7b		0,756786885	-0,204004288
Ndufs1		0,756933967	0,591936747

Table S2.

Hnrnph2	0,756943522	0,184034348
Ighg2b;Igh-3	0,756967742	-0,251814524
Aifm2	0,757016837	0,17670695
Tmlhe	0,757116809	-0,147211711
Hist1h1c	0,75712319	-0,100950877
Clic4	0,75715222	-0,066817602
Mtmr6	0,757800853	-0,123833338
Psma6	0,760582128	0,070327759
Copz1	0,761769668	-0,126850128
Akr1a1	0,762733949	0,10345459
Map1lc3b	0,763598295	0,238445918
Prrc1	0,76394601	-0,123466492
Vil1	0,764562367	-0,122445424
Gpx4	0,764564127	-0,220837275
Klc2	0,764743371	-0,220960617
Flii	0,764818547	0,095791499
Pgm3	0,765157048	0,112448374
Rdh13	0,765319461	0,140954336
Stx18	0,765476123	0,120519002
Rplp0	0,766533428	0,11376063
Naa40	0,76660397	-0,323343277
Ide	0,76663862	0,110925039
Nadsyn1	0,768358999	-0,152125676
Dolpp1	0,770133648	0,331064224
Acot9	0,770208913	0,081571579
Mxra7	0,77028423	-0,152282079
Ap1ar	0,770293396	-0,197710037
Ephx1	0,770346063	-0,206560771
Cobl	0,770422841	-0,157311757
Alg11	0,770453409	-0,174512227
Hspa13	0,770533868	-0,173954646
Vcp	0,771041431	0,092867533
Cc2d1a	0,771152143	-0,129581451
Dapk3	0,771193784	0,086291631
Hyou1	0,771333804	0,168001811
Ncf2	0,771408909	0,617612203
Rab2a	0,771420099	0,14622879
Klk1	0,771440151	0,349878947
G6pdx	0,771888	-0,10403951
Ndufb7	0,772526935	-0,075833639
Serpini2	0,7725381	0,916845322
Akr1c12	0,773294145	0,118476868
Get4	0,773406345	0,188489914
Fermt1	0,773563705	-0,1853803
Gm10881	0,773671992	0,567902247
Mtx2	0,773812544	-0,100286484
Hbs1l	0,773948333	0,09528923
Chchd1	0,775193238	0,465521495
Cpa1	0,775250117	0,54538091
Top1mt	0,775389815	-0,163310369
Dnm2	0,775492019	-0,074847539
Uqcc2	0,77612758	-0,166292191

Table S2.

Zmat2	0,77622707	0,146505992
Usp9x	0,77694068	-0,080464045
Obfc1	0,777065604	-0,164432526
Ddah2	0,777134552	0,151210785
Xrn2	0,777247715	-0,086743037
Mrpl10	0,777252752	0,1920681
Naaa	0,777742389	0,152917862
Stx16	0,777963483	-0,169299444
	0,778035071	-0,060399373
Oxsm	0,778125029	-0,147327423
Vdac1	0,778171188	0,200411479
Upp1	0,778243743	-0,119234721
Plec	0,778303768	-0,068124135
Thumpd1	0,778425831	0,212328593
Dnajb11	0,778772324	-0,161421458
Prcc	0,779516935	0,158288956
Gstm5	0,779548598	-0,114596685
Kiaa1033	0,779623276	-0,143564224
haemaglobin alpha 2;Hba	0,780191499	-0,300051371
Ndufs3	0,780505135	-0,13172849
Tmem19	0,780636937	0,136210759
Inmt	0,781378763	0,303754807
Hist1h2br;Hist1h2bp;Hist1h2bk;Hist1h2b	0,782018665	0,519486109
Cbs	0,782086307	1,112691879
Ccdc47	0,782317164	0,079969406
Vwa9	0,783270692	0,347935359
Ndufab1	0,784899767	-0,074176153
Bcap29	0,785646236	0,166805903
Dpysl2	0,7872274	0,094216665
Snrpc	0,787385977	-0,077482224
Acsf2	0,787771775	0,089129766
Capza1	0,788223516	0,10053126
Pgp	0,789204001	-0,134660721
Chchd6	0,789237785	0,125659943
Sult1c2	0,789397254	-0,395683289
Ap2b1	0,789581006	-0,07648468
Scaf8	0,789745116	-0,114562988
Lama5	0,79087561	-0,102618535
Rpl23a	0,792234146	0,114159266
Lmna	0,792255576	-0,08380127
Amdhd2	0,792269642	-0,151423772
Add1	0,79229726	0,071546555
Plekha2	0,792362538	0,362620672
Mms19	0,792748549	-0,145800273
Kdelc2	0,794171773	0,187555949
Gp2	0,794193548	0,455769857
Fbll1	0,794260264	0,146633148
Yars	0,794291415	-0,160831451
Eif4e	0,794912801	0,120167414
Cap1	0,796104799	0,067082723
Pin4	0,796575666	0,201510111
Mrpl16	0,796687993	-0,146311442

Table S2.

Vps4b	0,796780375	0,082852681
Atp6v1g1	0,796947709	0,124358495
Ifi27l2b	0,796964815	-0,106194814
Lsm1	0,797011345	0,267982483
Dennd4c	0,797039611	0,187838236
Gsta3	0,797096386	0,225227356
Ahnak2	0,797195924	0,139849345
Pccb	0,797397178	0,142970403
Serpina3n	0,797688396	0,192478816
Jup	0,797764624	-0,073434194
Pcca	0,797796485	-0,150836309
Ube2i	0,798016177	0,060587565
Vars	0,79815015	0,131703695
Ppib	0,798224584	0,195037206
Vtn	0,798828683	0,335817337
Tomm7	0,79883868	-0,206824621
Vmp1	0,798894834	-0,092156728
Slc25a11	0,798925952	-0,099247615
Asrgl1	0,798953392	0,328306834
A1cf	0,798999077	-0,25345548
Dcp1a	0,79915493	-0,180592855
Ube2k	0,799163048	0,099764506
Cd200	0,799597049	-0,133363724
Rps3a	0,800011971	0,096558889
Syk	0,800070984	-0,097588857
Tnxb	0,800117891	0,16361173
Rcn2	0,800205435	0,087204615
St13	0,80023589	0,073913574
Prdx3	0,800252186	0,099582036
Lamtor2	0,80037235	-0,419318517
Akr7a2	0,800409666	0,115389506
Pfn1	0,800478491	0,064678192
Fntb	0,800549885	-0,1256663
Mrpl2	0,80070545	0,138090134
Pcbp2	0,800722171	-0,062253316
Slmap	0,803031947	-0,0977904
Ppp5c	0,803230875	0,104848862
Sod2	0,803236213	-0,078902562
Arl8b	0,803816303	0,095702489
Slc7a1	0,803945797	0,259254456
H6pd	0,803993572	-0,077138901
Hspa12b	0,804807886	-0,103953679
Derl3	0,804878093	-0,123333613
Nid1	0,80496264	0,058815638
Ube3a	0,804965834	-0,092513402
Tax1bp1	0,805066789	0,177595139
Grb2	0,805080734	-0,086498896
Itpr1	0,805092325	0,101970673
Prps1l1	0,805123451	0,050100962
Mocs2	0,805238816	0,305164337
Adi1	0,805596885	-0,091653824
Nup98	0,805880467	-0,120347341

Table S2.

Hebp1		0,806569597	0,121949514
Mllt4		0,807509382	-0,090224584
Krt8		0,807694209	-0,071172078
	Sep-06	0,808762297	0,131544113
Col6a2		0,80920558	0,099858602
Eif4a1		0,809294602	0,061585744
Abcg2		0,809702789	0,423622767
Ddrgk1		0,809947898	0,146929423
Slc9a8		0,810034286	0,092359543
Cdk5rap3		0,811750571	-0,164686839
Tpm1		0,811796207	-0,119715373
Txndc15		0,811874001	-0,36890475
Pcdh1		0,811883105	0,126054764
Ckap4		0,811895867	0,076011658
Iba57		0,812373346	-0,358575185
Acyp1		0,813155373	0,095168432
Esyt2		0,81332208	-0,076416016
Ywhaq		0,813694482	-0,049079895
Clint1		0,813780616	0,055061976
Elp3		0,814484959	0,102603277
Rbbp9		0,814670618	0,071028392
Sdr39u1		0,814984735	-0,099592845
Sltm		0,8150041	-0,105137507
Ctbp2		0,816163972	-0,057615916
Ythdf1		0,816448998	-0,109861374
Cpsf3		0,816791259	0,108084997
Kpna1		0,817126991	-0,178601583
Nbeal2		0,817685552	-0,103527069
Ano10		0,818526268	0,054959615
Mrpl30		0,818639672	-0,10066096
Pes1		0,818902068	-0,094600677
Gm28036;Rbm12		0,819022283	-0,159560521
Impdh2		0,819088182	-0,063511531
Chd4		0,819202546	0,063317617
Dohh		0,81929214	0,193020503
G6pc		0,820228026	-0,242103577
Alg3		0,820627611	0,157677968
Tram1		0,820638365	0,268491109
Dbi		0,822075352	0,199564616
Efhd2		0,822769231	-0,134777069
Rab5b		0,823240988	0,113160451
Mthfd2		0,823264065	-0,215682348
Tbck		0,823264232	-0,221737544
Neurl3		0,823303401	-0,181180318
Wdr48		0,82443155	-0,087447484
Vwa1		0,825376841	-0,165120443
Tmod3		0,825397961	-0,059574127
Bet1		0,825564114	0,089026769
Anxa5		0,826296062	0,09349378
Pin1;Pin1rt1		0,826435137	0,0855395
Calb2		0,826530797	-0,112056732
Aup1		0,826580674	0,072045644

Table S2.

Ppap2c	0,827304977	-0,196402232
Entpd2	0,827425729	0,186867396
Pbrm1	0,827539471	-0,088111242
Arpp19	0,82758209	0,139192581
Itgav	0,827914124	0,108172099
Zrsr2	0,827997288	0,253648758
Acss2	0,82868685	0,081401825
Eif3f	0,82893246	-0,048737208
Ddx47	0,82900813	0,268149694
Cd68	0,829070219	-0,175824483
Dysf	0,82910404	0,23575592
Eif3c	0,829181038	-0,060112
Ndufa2	0,830333935	0,087015152
Pmm2	0,830469025	-0,087151845
Auh	0,830482199	-0,098856608
Atp1a2;Atp1a3	0,830669371	-0,245307922
Actl6a	0,830731289	0,117670695
Emc7	0,830850056	0,09344101
Ptpn6	0,831013983	0,062009811
Pdgfrb	0,831063388	-0,102485657
Asph	0,8314	-0,055915197
Eif4a2	0,831519027	0,064652125
Ilvbl	0,831752364	-0,099899292
Pfkm	0,832157551	0,066368103
Slc39a7	0,83380378	0,34462738
Cpne3	0,833831347	-0,066145579
Carkd	0,833962213	0,142958323
Hgfac	0,834044544	0,124579748
Nap1l1	0,834347122	-0,105511347
Sephs2	0,834511126	-0,075656255
Dad1	0,834677753	-0,114853541
Git1	0,836371153	0,109539032
Xrcc6	0,836459119	-0,087774913
Mgll	0,836751853	0,051303228
Glrx5	0,836880108	-0,134133657
Tapbp	0,837098092	0,064005534
Suox	0,837546679	0,041182836
Dnajc25	0,837942114	-0,083830516
Svip	0,838049349	-0,128426234
Psme3	0,839509307	-0,061101913
Tubg1;Tubg2	0,840313004	-0,100943883
Acad8	0,841033849	-0,115752538
Dab2ip	0,841465382	-0,098092397
Zc3h4	0,841476468	0,163387299
Galk1	0,842896057	0,088350932
Rsl1d1	0,844021501	0,067417781
Hsd17b13	0,844111882	-0,347372055
Itch	0,844116409	-0,061787923
Trim65	0,844229288	0,073524475
Rhot1	0,844300806	-0,05632782
Psmc6	0,845540045	0,062484741
Uba1	0,847203936	0,040859222

Table S2.

Cox4i1	0,847370163	0,05900828
Pld4	0,847797004	-0,158088684
Slc43a2	0,848608851	-0,070966721
Pycr1	0,848870615	0,209927241
Cfb;Gm20547	0,849224307	-0,099754333
Fam98c	0,849282109	-0,068418503
Xab2	0,849403305	-0,153871536
Chmp7	0,851814244	-0,115952174
Hid1	0,852177679	0,102858861
Hip1	0,852297255	0,069304784
Gpd2	0,853165291	0,052440643
Hnf4a	0,853182508	0,063908895
Espn	0,854396967	-0,090337118
Tcerg1	0,854663099	-0,074995677
Fdx1	0,855045029	0,084241867
Mul1	0,855708491	-0,096675237
Nudt7	0,855721925	0,139186223
Metap2	0,856629093	-0,067708333
Adnp	0,856993318	0,11303393
Cops2	0,858292585	-0,046418508
Arf6	0,858629564	-0,068482717
	0,859095482	-0,092045466
Cox7a2	0,859195372	0,131345749
Stau1	0,859373526	0,193169912
Lyz1	0,860634342	0,111476898
Saa4	0,861576701	0,242239634
Ppp2r5b	0,861745163	0,056094488
Tkt	0,861768393	0,064537048
Lrrn4	0,861845333	0,218772888
Os9	0,862376721	0,063067754
Ap2s1	0,862425239	-0,129138311
Mrps18b	0,862637655	0,062636058
Itsn2	0,862642683	-0,102441788
Pkp2	0,862664005	-0,05286026
Stk4	0,862678793	0,075866699
Prph	0,862749501	-0,068464915
Syncrip	0,86286285	-0,051596959
Sri	0,862875028	-0,065124512
Ppp6c	0,863045001	-0,072647731
Sfpq	0,863124612	0,050535838
Hdhd3	0,863727011	-0,065921148
Stt3b	0,863874113	-0,067073186
Tmem86b	0,864698272	-0,356186549
Syn2	0,864770764	-0,108930588
Myh9	0,864784418	-0,047217051
Apoa1bp	0,864925393	0,129461924
Maob	0,864976085	-0,071678797
Atp6v1e1	0,865535738	0,059335073
Tmx1	0,865887611	-0,085196177
Itpr3	0,866294183	-0,043264389
Ddx23	0,866615657	0,062730789
Ndufaf2	0,866797258	0,076941172

Table S2.

Cd151	0,869977905	-0,075143178
Ppm1h	0,87009107	-0,290943782
Bola2	0,870124199	0,095997492
Ttc1	0,870310954	0,160839717
Capns1	0,870503203	-0,055587133
Fmnl2;Fmnl1	0,870570419	-0,094199498
Cyb5a	0,870762641	-0,093357086
Pitrm1	0,870783492	0,071147919
Inpp5d	0,871447485	0,0692571
Syne1	0,871586237	0,196930567
Ddx39a	0,871705272	-0,092573166
Ctnnb1	0,87187478	-0,057554881
Arpc3	0,871925942	0,095974604
Agk	0,87198677	0,074077606
Trappc12	0,872034376	0,133893967
Ilk	0,872054638	0,044697444
Tbcc	0,872311826	-0,142921448
Rpl9	0,872412335	0,072677612
Anxa3	0,87356583	0,061223984
Nudt16	0,873969624	0,062955221
Hadhb	0,875275528	-0,043842316
Pef1	0,875400308	0,135486603
Mrpl15	0,875507151	-0,081987381
Tcea3	0,875520668	-0,161188126
Sec11a	0,875546074	-0,099006653
Mrpl23	0,875738671	-0,087755839
Rad23b	0,876086154	-0,059302648
Arcn1	0,876486926	-0,045711517
Zfp947	0,876727592	-0,408603668
Hist1h2al;Hist2h2aa1;Hist2h2ac	0,876881862	-0,191135406
Ppp1cb	0,876971667	0,054103851
Ccnh	0,879339917	-0,137022654
Wdr77	0,879369022	0,138392766
Man2a1	0,879468421	-0,068639119
Nudt3	0,879532924	0,11057663
Pagr1a	0,879553411	-0,086070379
Eno1	0,879680351	-0,045931498
C530008M17Rik;Kiaa1211	0,880235758	-0,110977173
Fh	0,880297238	-0,047125498
Mrps21	0,880370094	-0,157269796
Ahsg	0,880383081	0,115662893
Tbl2	0,880718196	-0,09001859
Col6a1	0,880814183	-0,060977936
Hint3	0,880833114	-0,121994019
Pdia4	0,880875657	0,065869649
Hist1h1a	0,881003286	0,06172053
Gga1	0,881174617	-0,06440862
Hydin	0,881409757	0,132545471
Arl6ip5	0,881486439	-0,042437871
Nup88	0,881595977	-0,227359772
Rabgap1l	0,881687295	-0,08815829
Mocos	0,882523279	0,208670934

Table S2.

Bcap31	0,883381119	-0,04863739
Try10	0,884301376	0,232028325
Cnot11	0,884311053	-0,136202494
Sppl2a	0,884420363	-0,057148616
Fam20b	0,884510044	-0,104890823
Mta2	0,885599825	0,050897598
Chdh	0,885623745	-0,099184672
Rpl22l1	0,885654962	-0,145321528
Ppt1	0,885771666	0,063899994
ORF11	0,885777487	-0,068194071
Gpa33	0,886273005	-0,08068339
Ralgapb	0,886664923	-0,055790583
Thg1l	0,888061029	-0,103916168
Aldh6a1	0,889078231	0,164808273
Aftph	0,890145938	0,076000214
Palm	0,89015512	0,084339142
Arglu1	0,890543554	0,046789169
Gatad2a	0,890831265	-0,059438705
Gm20441	0,891710927	0,061507543
Vrk3	0,891803264	-0,078281403
Osbpl1a	0,89203653	0,112159093
Slc27a2	0,892062636	0,078231812
Celf1	0,892229715	0,054466883
Hnrnpm	0,892354221	-0,152276357
Rps7;Gm9493	0,893564348	0,084136963
Tmed5	0,89400739	0,097836812
Cops8	0,894101695	0,061611176
Iqgap1	0,894135129	0,038941701
Paox	0,894428323	0,062163035
Eif5b	0,894682874	-0,060184479
Ppp2r5d	0,894766558	-0,0560112
Sssca1	0,894823274	-0,062676112
Opa3	0,894924479	-0,087250392
Nck2	0,895449772	-0,105677923
Dbt	0,896619523	-0,108313243
Ass1;Gm5424	0,896907395	0,072044373
Nudc	0,897615785	-0,03997612
Scyl2	0,897939736	0,062648773
Nup50	0,898713481	-0,036485672
H2-Ab1	0,899772914	0,689419429
Mrps7	0,900426343	-0,0669988
Rock1	0,900932207	-0,044758479
Pkp4	0,900936797	-0,112295787
Lamc1	0,901022732	0,043060939
Srp68	0,901217843	0,064961116
Gm9774;Adrm1	0,903075659	-0,049648921
Capza2	0,903163314	-0,035901388
Rpl35;Gm10269	0,903163495	0,052098592
Zbtb8os	0,903251027	0,046209335
Ighg3	0,903333622	-0,227384567
Tff3	0,903405324	-0,086596807
Ago2	0,904050994	0,069024404

Table S2.

Gls	0,904173688	0,208260854
Oxnad1	0,904179814	0,051095327
Tollip	0,90607689	0,080608368
Srp72	0,906443533	-0,071986516
Lsm2	0,906978411	0,086048126
Fasn	0,907273198	-0,037481308
Col15a1	0,907357652	0,064612707
Osgep	0,908123409	0,049130758
Arfip2	0,908495903	-0,064198812
Aacs	0,908589174	-0,058827082
Abcb8	0,908599655	-0,053986231
Agpat5	0,908942444	-0,084646861
Galm	0,909459483	-0,071489334
Aldh5a1	0,910234863	-0,03399531
Pzp;A2m	0,911658768	0,058368047
Orm1	0,91195524	0,073596954
Rtn1	0,91212656	0,063085556
Rpl10a	0,912145533	0,056237539
Rrbp1	0,912184165	-0,056634267
Rab1b	0,912205901	-0,039564133
Acaa2	0,912341947	-0,051134745
Arpc2	0,912728759	0,033984502
Rab6a;Rab6b	0,912821505	0,042565664
Fermt2	0,913278865	0,03844897
Txnl1	0,913641445	-0,053941091
Arih1	0,913927359	-0,082068125
Ighv14-2	0,913947595	-0,332802455
Col18a1	0,914027068	-0,052096685
S100a6	0,914056725	-0,0534935
Fbxo6	0,915385871	0,076861699
Bgn	0,915769	-0,0554142
Brix1	0,917908564	-0,070753098
Ccdc43	0,918305579	0,077665329
Lars	0,918474147	0,049975077
H2-Aa	0,918582887	0,306191762
Arhgef2	0,918779923	0,089284261
Dhx30	0,920327616	-0,102294286
Poldip3	0,920900129	0,046169917
Nid2	0,92100493	0,033813477
Ss18	0,921820908	-0,06394577
Map7	0,921947289	-0,070232391
Mtap	0,921964446	0,038584391
Itgb3	0,922764026	-0,030611674
Prpf40a	0,923225862	-0,053935369
Ccl6	0,92348887	0,077713648
Fmo5	0,923830516	-0,05891482
Abhd14b	0,92416	-0,065260569
Sntb2	0,924243902	0,048725128
Emc10	0,925254063	-0,058889389
Gtpbp4	0,925575369	0,035907745
Cdipt	0,925853784	0,069959005
Npm1;Gm5611	0,927029707	-0,040200551

Table S2.

Pigo	0,927033333	0,041285833
Pcbd1	0,927040376	0,085495631
Cars	0,927219991	-0,073837916
Slc7a7	0,927627589	-0,066333771
Mrpl24	0,927952208	0,090088526
Smg1	0,927974381	0,042597453
Sec24a	0,928098934	-0,120003382
Defa24	0,928150235	0,038459778
Brcc3	0,9282508	0,036104838
Ric8a	0,92832444	-0,097873052
Rsu1	0,928361543	-0,033231099
Hm13;H13	0,928377986	-0,059882482
	0,928805283	0,095602036
Ndufa8	0,92888046	-0,029068629
Exosc8	0,929059676	0,087497075
Cd9	0,929229131	0,102725983
Unc45b	0,929248136	0,121449788
Pdia3	0,92943581	-0,022455851
Rabggtb	0,929461047	0,141277949
Arhgef16	0,930302979	0,046892802
Tbc1d13	0,930382816	0,035229365
Acsl1	0,930454139	-0,037452062
Sptlc1	0,930459902	-0,051266988
Shoc2	0,930797363	0,066344579
Sec23ip	0,930934524	0,030124664
Exoc1	0,93120442	0,120101293
Vps33b	0,931271413	0,028401693
Tmed11	0,931450181	-0,417737961
Pdk2	0,931595582	-0,041345596
Ehd2	0,931994904	-0,021222432
Sun1	0,932004244	0,076295217
Rabgap1	0,93201868	0,039838791
Igkv4-70;Igkv4-59;Igkv4-86	0,932080697	-0,081277847
Rbm10	0,933316928	-0,036830902
Prss2	0,933411665	0,158233643
L2hgdh	0,933424936	-0,048892975
Dmbt1	0,933466582	-0,125090281
Ociad2	0,933735001	0,030441284
Tmem214	0,935312421	-0,082899729
Cnot2	0,935418043	0,070800145
B2m	0,935462206	-0,064084371
Anxa4	0,9355018	-0,034786224
Irf6	0,935676271	0,039416631
Gtf2i	0,935706718	-0,02177302
Fam96a	0,935904318	0,115432739
Vti1b	0,93591873	-0,037517548
Sdc4	0,936314008	-0,080013275
Tsnax	0,9381392	0,024117788
Mug1;Mug2	0,938329949	-0,057631175
Tmed2	0,938932544	-0,050711314
Nck1	0,939649894	0,04572169
Jund	0,939728713	-0,088850657

Table S2.

0610011F06Rik	0,939880811	0,037215551
Fabp2	0,940079476	-0,05302302
Praf2	0,940373469	0,052216848
Pgm2	0,942062936	0,02677536
Reg3b	0,94217138	0,156745275
Mrps6	0,942269426	0,050434748
Sms	0,942322145	0,106150945
Qtrt1	0,943319688	-0,090737661
Gss	0,943825353	-0,023485819
Pcna	0,94384135	0,058045705
2210016F16Rik	0,944186419	-0,041702906
Cfd	0,944457095	0,083906174
Fam109a	0,944477234	0,117848078
Fam136a	0,945157429	-0,057124456
Actr2	0,945474616	0,021879196
Pter	0,945673831	-0,041248957
Inpp5a	0,946484734	-0,029050191
Ccbl1	0,946562592	0,029565811
Fam192a	0,946620893	0,04206721
Naa35	0,946668632	-0,023328145
Tm9sf2	0,946685185	-0,027231216
Gps1	0,946824668	0,025698344
Dock7	0,948188473	-0,031548818
Lin7c	0,948699454	-0,026383718
Slc51a	0,94882489	0,064737956
Ccdc91	0,948844407	0,08729744
Rps25	0,948847108	-0,046782811
Acy3	0,949156545	0,048041662
Sc5d	0,949242017	-0,056406657
Mpp1	0,949505566	0,030394872
Plg	0,949850157	-0,047363917
Insr	0,94993199	0,058437983
Gca	0,95001008	-0,038606008
Tmem109	0,950014697	-0,046085358
C5	0,95038355	0,030230204
Ube2o	0,950646528	0,060469945
Mfsd10	0,951910235	-0,127775828
Trim2	0,951979031	-0,04923439
Eftud2	0,952384744	0,021448771
Stxbp5	0,952419199	-0,018892288
Lama2	0,952549266	0,054217021
Rab3ip	0,952553949	0,037799199
Psmb8	0,953357068	-0,013142268
	0,953483871	0,044867198
Myo1b	0,954747069	-0,027657827
Cryab	0,954913125	0,045883179
Cdh17	0,955165341	0,020640055
Ptgr2	0,955528772	0,029930115
Lgmn	0,955539749	0,025962194
Akr1b1	0,955793392	0,019005458
Igkv9-124	0,955805898	-0,186002096
Mrpl43	0,95673913	-0,050177892

Table S2.

Gstp1;Gstp2	0,956779427	0,032114665
Idh3g	0,957860815	0,022096634
Mcam	0,958160468	0,023121516
Hist1h2bc;Hist2h2bb;Hist1h2bh;Hist1h2l	0,958941299	-0,021251678
Luc7l2	0,958998329	0,016963959
Nono	0,959483817	-0,027441025
Ighv4-1	0,959583299	0,069180806
Aip	0,960161937	-0,018332799
Hsp90b1	0,960241286	-0,02302297
Hexa	0,960452138	-0,040749232
Abcb10	0,960460576	-0,048604329
Ttc9c	0,960590862	-0,028320948
Map2k6	0,961043369	0,071922302
Slc35e1	0,961459245	0,047270457
Khk	0,96168215	0,19372495
Dnajb1	0,9616875	0,01806132
Atp1a1	0,961804543	0,018807729
Rps4x	0,961979158	0,02589035
Sec61a1;Sec61a2	0,962286904	0,037380854
Ighv7-3;Ighv7-2	0,962442316	0,074417114
Cdk7	0,962761865	0,024194082
Abhd16a	0,963308221	-0,03501002
Pura	0,963530587	0,011833827
B4galt1	0,963802788	0,025587718
Ighv10-1;Ighv10-3	0,964425125	-0,048823039
Faf1	0,964798503	-0,035756429
Hck	0,96483659	-0,032180786
Psmc3	0,965257119	-0,016542435
Scaf11	0,965268495	0,021458944
Tmem87a	0,96540536	0,05315272
Cntnap5c	0,965423884	0,078005473
Cbx1	0,965569442	0,034443537
Jak1	0,965624429	-0,036840439
Mapk3	0,965714286	-0,030275981
Ppp2ca	0,965755551	-0,017084122
Mroh1	0,96577418	0,06214269
Nudt2	0,965881328	0,039800644
Cyp20a1	0,966536181	-0,028239568
Ctr9	0,966556616	-0,031298319
Acox3	0,96668409	-0,027472814
Hsd17b13	0,96744799	-0,102136612
Srsf11	0,967501243	0,024714788
Gnai1	0,96753658	0,032295863
Vamp7	0,967570748	-0,040086746
Lman2	0,967608624	0,012759527
Bsg	0,968182909	-0,018810908
Dhcr24	0,968209437	0,020605723
Rab3gap2	0,968249224	-0,021774292
Babam1	0,968263964	0,015858332
Mtatp8	0,968279628	0,024113337
Rpn2	0,968289087	0,022969564
Pygm	0,968449689	0,025322596

Table S2.

Tbc1d1		0,968490488	0,052918116
Ankrd13a		0,969062642	-0,052774429
Cpe		0,969074824	0,032443364
Azgp1		0,969675553	-0,069948196
Ndufa11		0,970031812	0,023691813
Ampd3		0,970136307	0,023999532
Gtf3c4		0,970144628	0,06252861
Cmpk1		0,972102416	0,013630549
Pycr2		0,972270851	0,025499344
Slc25a51		0,972510305	0,020249685
Rps6ka3		0,972543016	-0,01264445
Kpna6		0,972548115	0,015484492
Pdxk		0,972580272	-0,036881765
Grwd1		0,972639868	-0,027048747
Pm20d1		0,972690452	-0,04384613
H2afy2		0,972780528	0,018274943
Igkv1-135;Igkv1-133		0,972781011	0,048659007
Clu		0,972790928	-0,052336375
Smarca4		0,97285932	-0,012941996
Commd2		0,973016893	-0,021993637
Gpi		0,973976936	-0,013299306
Ptges3		0,975496912	0,016791026
Apip		0,975566811	0,047499339
	Sep-10	0,976074074	0,02222379
Prpf8		0,97625602	0,011184692
Cdk1		0,976630757	0,038849513
Cnn3		0,976758111	0,02063179
		0,976831585	0,063369751
Spg7		0,976838643	0,007837296
Rqcd1		0,976888889	0,028296153
Plekhf2		0,976947412	0,035147349
Ssb		0,977011315	0,009916306
Tmf1		0,977013575	-0,031407674
Rbp1		0,977076355	0,027977626
Serpina1a;Serpina1c		0,977080748	0,019730886
Mapkapk2		0,97708412	0,016279221
Fam63a		0,977129026	-0,011530558
Hnrnpr		0,977152487	0,013601939
H2-D1		0,977188257	-0,022406896
Npepl1		0,97728463	-0,01563708
Parp1		0,97767398	-0,01508077
Ppil1		0,977837572	0,014932632
Gars		0,977923739	-0,014754613
Gmps		0,978156871	-0,005628586
Nup214		0,97820373	0,013284683
Snx27		0,978226137	0,018526077
Snrpe		0,978327049	-0,020694733
Gucy1a3		0,978378378	-0,016970952
Etfa		0,978426552	-0,009568532
Cops6		0,978874923	-0,010392507
Nucb1		0,979209169	-0,01020813
Ppwd1		0,980572949	-0,009104411

Table S2.

Rhoa	0,980754239	-0,010300954
Ssr1	0,980871271	-0,028636297
Pak1	0,980967294	-0,012304942
Hibadh	0,980978541	-0,012287776
Nme1	0,98108347	-0,017651876
Gnai2	0,981110247	0,007921219
Ppp1r2	0,981143558	-0,011388143
Adk	0,981167859	0,020469666
Mars	0,981260621	0,009424845
Stambp	0,981577292	0,031632741
Usp14	0,982163332	0,007108053
Kat7	0,982907532	-0,014056524
Tjp1	0,983189884	0,007617315
Nsdhl	0,983269984	0,007663727
Sart1	0,983390453	0,017649968
Pex6	0,983408163	-0,013223012
Lcp1	0,983544583	-0,006579081
Itgb1	0,983956796	-0,005999247
Sar1a	0,984113331	0,013173421
Ppp1ca	0,984269113	0,007432302
Ostc	0,984303178	-0,024336497
Dis3	0,984342625	0,016827265
Mrpl44	0,984474231	-0,01180013
Mcat	0,984475376	0,009986242
Ddost	0,984503155	0,009159724
Herc4	0,984661238	0,00949351
Rab5c	0,984769043	-0,008760452
Psma5;Gm8394	0,984825061	0,008927663
Rnaseh2b	0,984834655	-0,009230932
Pgrmc1	0,984861739	-0,009346008
Tpm2	0,985079301	-0,015509288
Cyp27a1	0,985651555	-0,020965576
C1galt1	0,986309756	-0,008492152
Hdgfrp3	0,987476529	-0,00418663
Dhx9	0,987804304	0,005609512
Scaf4	0,987808247	0,006905238
Dpagt1	0,987827782	0,008902232
Cdh13	0,987857143	-0,015179952
Ado	0,987910696	0,007707596
Lims1	0,987948936	-0,004630407
Dhx36	0,98798781	0,015252431
Aass	0,988004873	-0,015753428
Vwa5a	0,988006486	-0,0035127
Mcmbp	0,988070603	-0,014859517
Lsm8	0,9880746	0,007789612
Psmb7	0,988145985	-0,008042653
Fubp3	0,988230146	0,003497442
Eif4g1	0,988325289	0,004491806
Bod1l	0,988486004	0,031203588
Coasy	0,988779599	-0,002854029
Surf4	0,988845687	0,006999969
Sh3bgr	0,988908502	-0,018702825

Table S2.

Tmem126a	0,988911116	0,004566193
Skp1	0,988921085	0,004147212
Lmnb1	0,989005469	-0,004849116
Ahcy	0,989225976	0,007541656
Yme1l1	0,989316343	-0,005468369
Plgrkt	0,989528817	0,006897608
Atad3	0,990046907	-0,003859838
Bphl	0,991779349	0,004576365
Col6a3	0,991928037	0,004014333
Ampd2	0,99197979	0,00918897
Ddx6	0,992186667	-0,003107707
Sf3b4	0,992342557	-0,005438487
Pla2g4a	0,992394992	0,002829234
Csde1	0,993223501	0,003044764
Fxr1	0,993550262	-0,003247579
Oxsr1	0,99491929	0,00373141
Galnt1	0,994974773	0,002575556
Ndufb3	0,995333871	0,001532237
Ubl4a	0,995372454	-0,00159963
Gtf2e2	0,995573215	0,003348668
Scgb2b2	0,995606213	-0,010388056
Arrb1	0,996801451	-0,003163656
Nek9	0,996951844	0,001483917
Manf	0,99700524	0,001948675
Rac1;Rac3	0,997272361	-0,001633962
Mfap2	0,997693919	0,003200531
Fbxl8	0,997786103	0,001401265
Dhfr	0,99803181	0,001079559
Snrpb;Snrpn	0,998661836	0,000431061
Psmd4	0,999512274	-0,00021553
Pcyox1	0,999574965	0,000301997
