# Supplementary material for: HUWE1 is a critical colonic tumour suppressor gene that prevents MYC signalling, DNA damage accumulation and tumour initiation
Source: EMBO Mol Med. 2016 Dec 22;9(2):181–97. doi: 10.15252/emmm.201606684 (PMC5286368; doi:10.15252/emmm.201606684)
Supplement: Supplementary file 2 — Expanded View Figures PDF [file EMMM-9-181-s002.pdf]

Expanded View Figures

Figure EV1. *Huwe1* is an intestinal tumour suppressor gene commonly mutated in human CRC.

A cBioportal OncoPrints of *HUWE1* mutation rate in CRC identified during sequencing projects.
B cBioportal OncoPrints of *HUWE1* mutation rates in a range of human cancers.
C UbcH7 pulldown of HUWE1 HECT domain containing point mutations identified in colorectal cancer sequencing projects.
D Quantification of total tumour numbers per colon in sacrificed *Vil Apc*, *Vil Apc Huwe1*^{het} and *Vil Apc Huwe1*^{hom} mice. Deletion of *Huwe1* led to a significant increase in the number of tumours per colon (Mann–Whitney, $n \geq 3$). Mean and standard deviation are plotted.

 © 2016 Cancer Research UK Beatson Institute

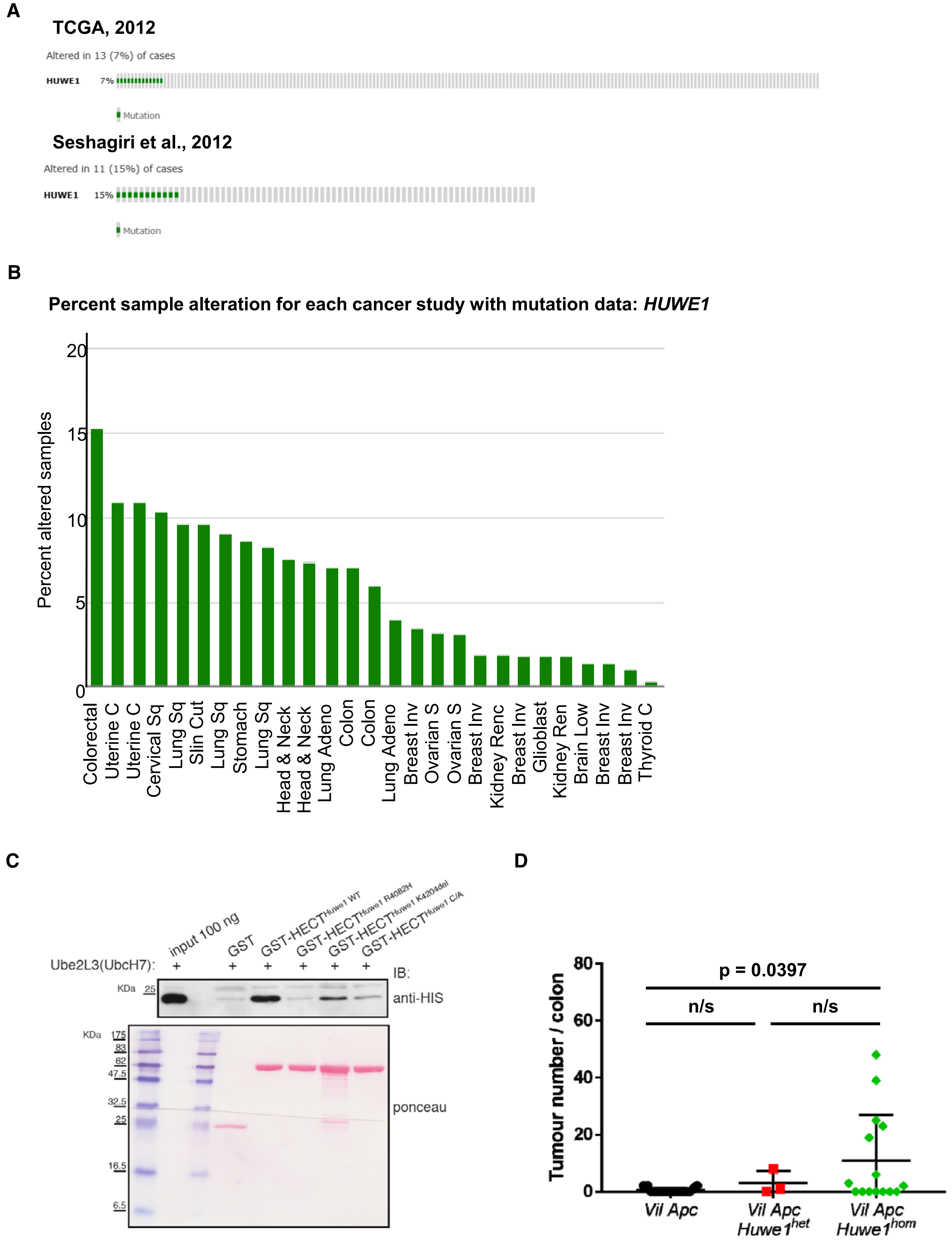
A
TCGA, 2012
Altered in 13 (7%) of cases
HUWE1 7%
Mutation
Seshagiri et al., 2012
Altered in 11 (15%) of cases
HUWE1 15%
Mutation
B
Percent sample alteration for each cancer study with mutation data: HUWE1
Percent altered samples
0
5
10
15
20
Colorectal
Uterine C
Uterine C
Cervical Sq
Lung Sq
Slin Cut
Lung Sq
Stomach
Lung Sq
Head & Neck
Head & Neck
Lung Adeno
Colon
Colon
Lung Adeno
Breast Inv
Ovarian S
Ovarian S
Breast Inv
Kidney Renc
Breast Inv
Glioblast
Kidney Ren
Brain Low
Breast Inv
Breast Inv
Thyroid C
C
input 100 ng
GST
GST-HECT Huwe1 WT
GST-HECT Huwe1 R4082H
GST-HECT Huwe1 K4204del
GST-HECT Huwe1 C/A
Ube2L3(UbcH7):
IB:
anti-HIS
KDa 25
KDa 175 83 62 47.5 32.5 25 16.5 6.5
ponceau
D
Tumour number / colon
p = 0.0397
n/s
n/s
0
20
40
60
80
Vil Apc
Vil Apc Huwe1 het
Vil Apc Huwe1 hom

Figure EV1.

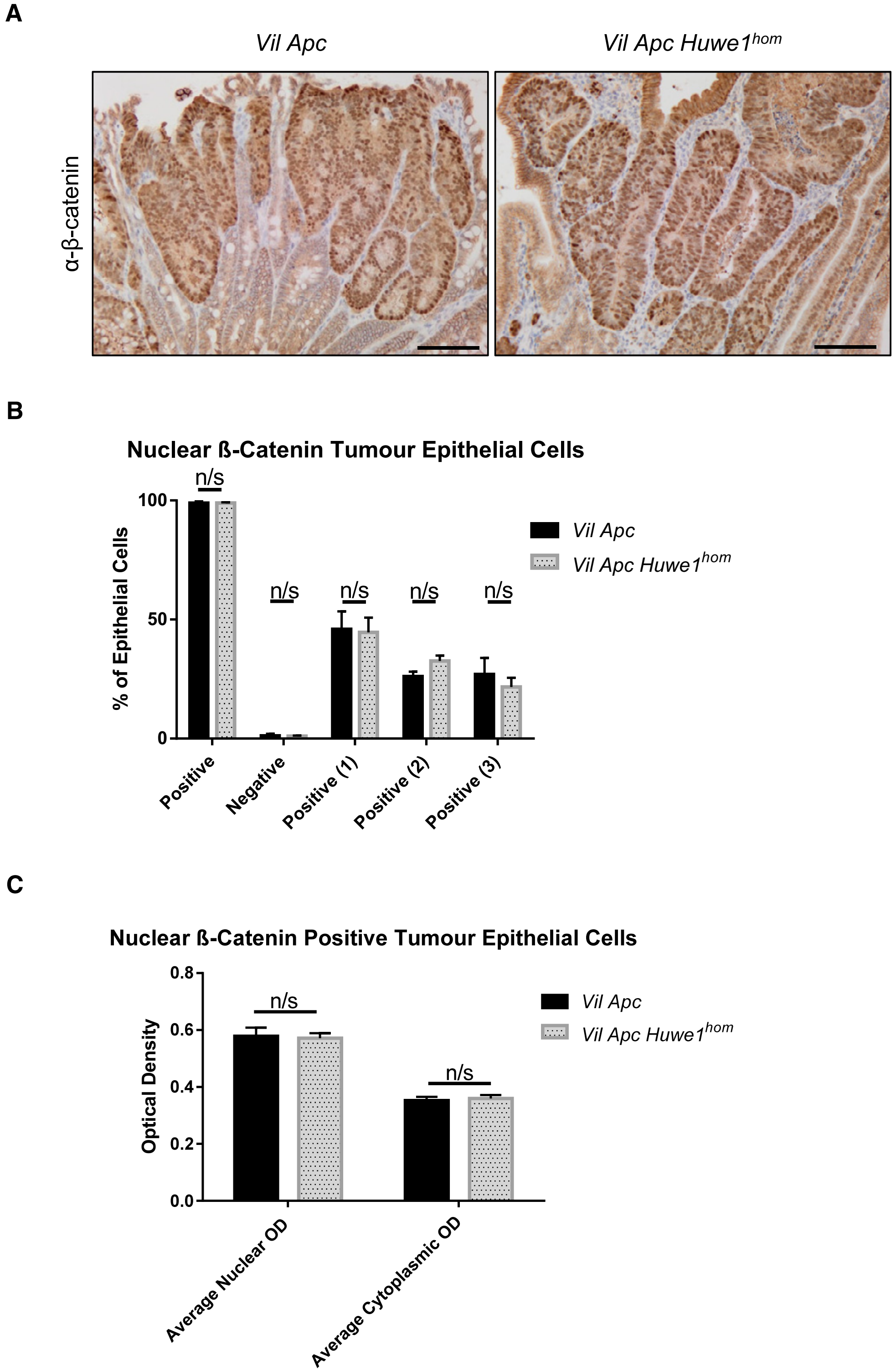

Figure EV2. *Huwe1*-deficient tumours display β-catenin nuclear localisation.

A β-Catenin IHC of *Vil Apc* and *Vil Apc Huwe1*^{hom} adenomas demonstrating nuclear localisation. Scale bars = 100 μm.

B Quantification of percentage of nuclear β-catenin-positive cells in *Vil Apc* and *Vil Apc Huwe1*^{hom} adenomas. Values 1, 2 and 3 represent low, medium and high OD, respectively (Mann–Whitney, n = 3 versus 3). Data are mean and SEM.

C Quantification of β-catenin staining intensity in *Vil Apc* and *Vil Apc Huwe1*^{hom} adenomas (Mann–Whitney, n = 3 versus 3). Data are mean and SEM.

 © 2016 Cancer Research UK Beatson Institute

Figure EV3. Intestinal homoeostasis following *Huwe1* deletion.

A Scoring of number of BrdU-positive cells per half-crypt in control and *Huwe1*-deficient small intestines (Mann–Whitney, n = 4 versus 4). Mean and SD are plotted.
B Lysozyme IHC demonstrating mislocalised lysozyme expression in *Huwe1*-deficient small intestine. Scale bars = 200 μm. Black arrows indicate mislocalized lysozyme positive staining.
C Quantification of lysozyme-positive cell mislocalisation (per cent of crypts displaying mislocalised lysozyme-positive cells). Significant lysozyme-positive cell mislocalisation observed upon *Huwe1* deletion (Mann–Whitney, P = 0.0179, n = 3 versus 5). Mean and SD are plotted.
D High-magnification image of lysozyme-positive villus cells in *Huwe1*-deficient intestine. Note typical goblet cell morphology (black arrows). Scale bar = 20 μm.
E qRT–PCR data of WNT target genes in control and *Huwe1*-deleted small intestine (Mann–Whitney, n = 4 versus 6, **P = 0.0048). Mean and SD are plotted.
F qRT–PCR data of WNT target genes in *Apc* and *Apc Huwe1*-deleted small intestine (Mann–Whitney, n = 3 versus 3, *P = 0.04). Mean and SD are plotted.
G β-Catenin IHC of control and *Huwe1*-deleted small intestine. Note no gross changes in β-catenin localisation following *Huwe1* deletion. Scale bars = 100 μm.

© 2016 Cancer Research UK Beatson Institute

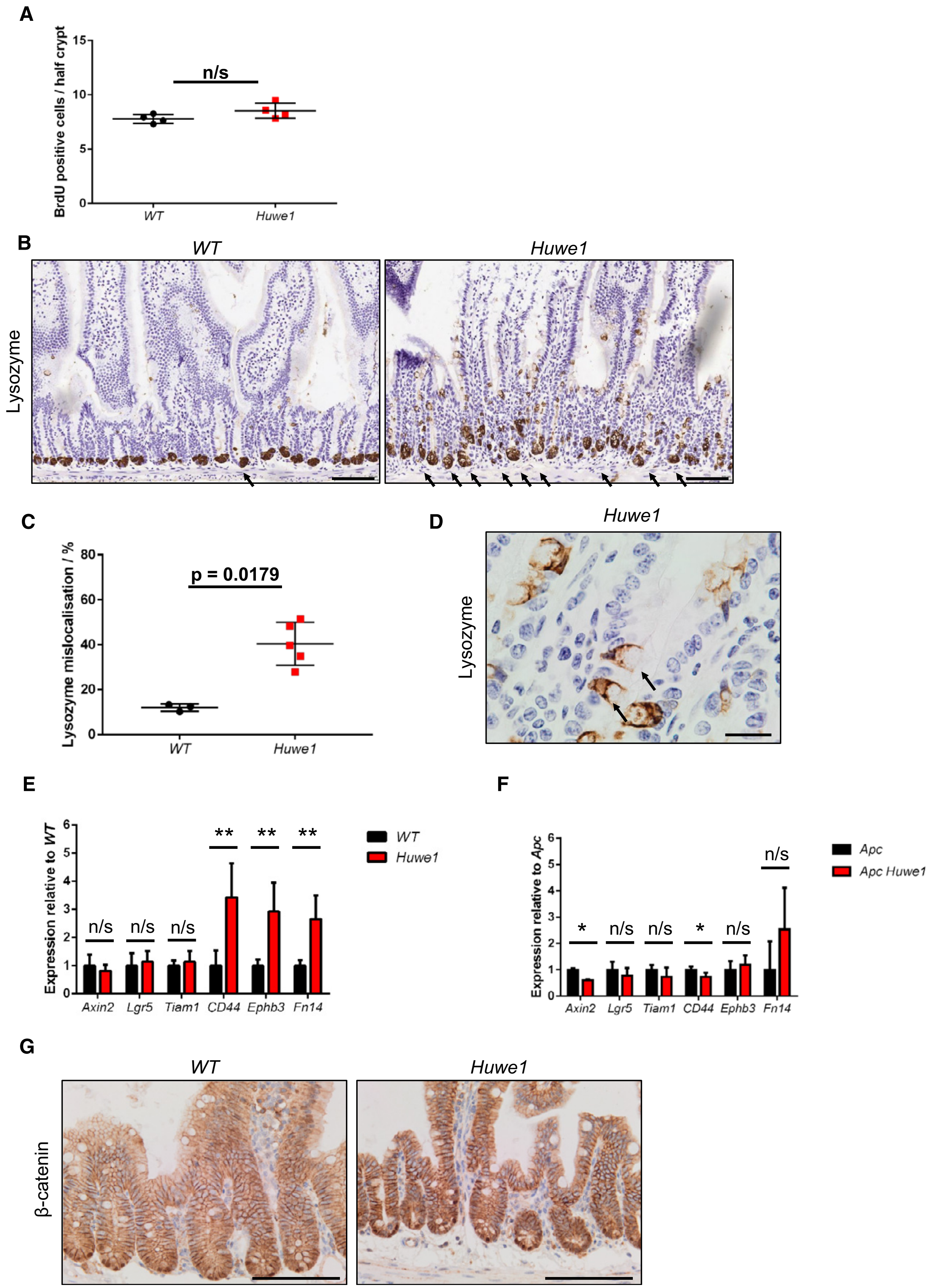

Figure EV3.

 © 2016 Cancer Research UK Beatson Institute

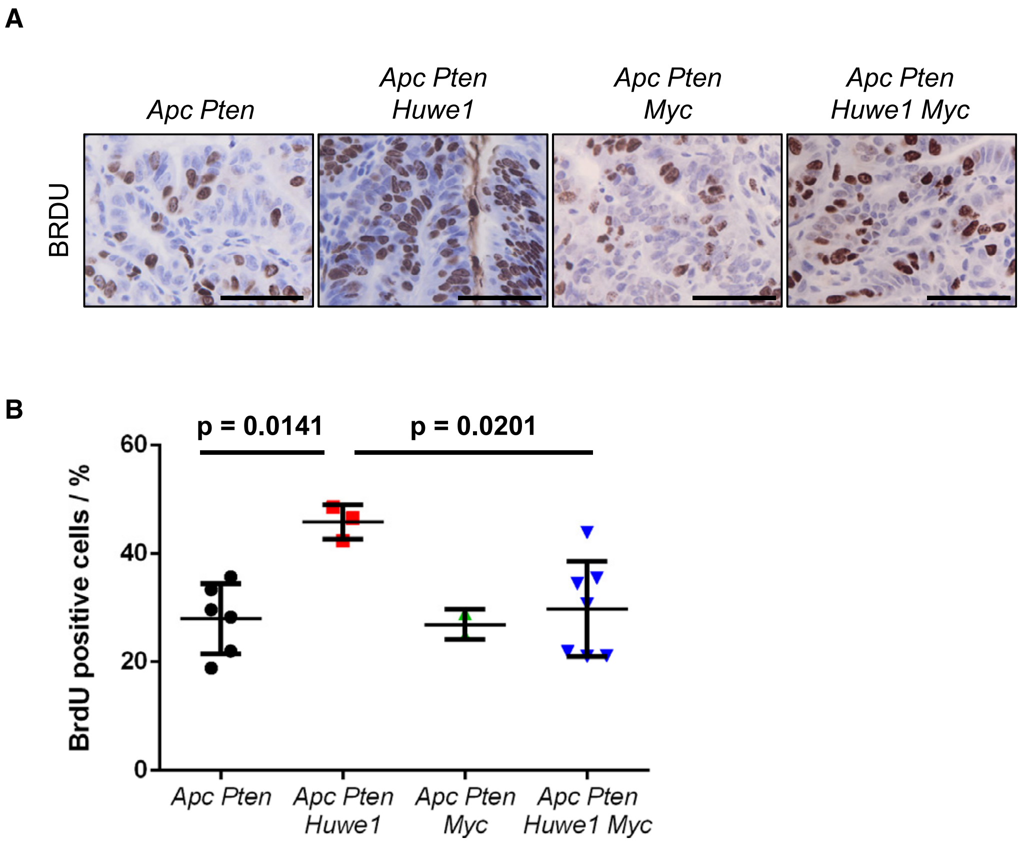

Figure EV4. HUWE1 suppresses tumourigenesis via regulation of MYC.

A BRDU IHC of *Vil Apc Pten*, *Vil Apc Pten Huwe1*, *Vil Apc Pten Myc* and *Vil Apc Pten Huwe1 Myc* tumours. Scale bars = 50 μm.

B Quantification of BrdU IHC in *Vil Apc Pten*, *Vil Apc Pten Huwe1*, *Vil Apc Pten Myc* and *Vil Apc Pten Huwe1 Myc* tumours (Mann–Whitney, $n \geq 3$). Data plotted are mean and SD.

© 2016 Cancer Research UK Beatson Institute

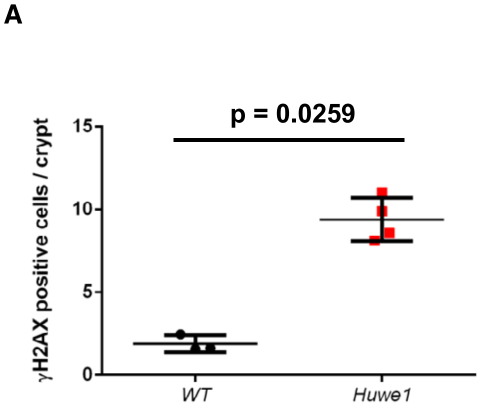

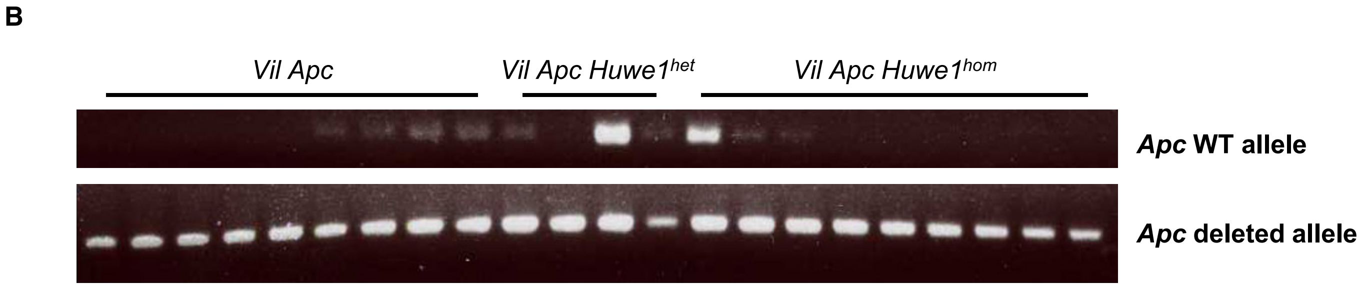

Figure EV5. *Huwe1*-deficient tumours display *Apc* LOH.

A Quantification of γ-H2AX IHC demonstrating increased number of nuclei staining positive following *Huwe1* deletion (Mann–Whitney, $n = 3$ versus 4). Data plotted are mean and SD.

B *Apc* LOH PCR analysis of tumours isolated from *Vil Apc*, *Vil Apc Huwe1*^{het} and *Vil Apc Huwe1*^{hom} mice. The majority of tumours display *Apc* LOH.

Figure EV6. *MCL1* protects *Huwe1*-deficient tumours from apoptosis.

A Quantification of scoring of apoptotic bodies in control, *Mcl1*$^{fl/+}$, *Huwe1*$^{fl/fl}$ and *Huwe1*$^{fl/fl}$ *Mcl*$^{1fl/+}$ deleted intestines (Mann-Whitney, $P = 0.0259$, $n = 3$ vs 4). Data are mean and SD.

B Classification of adenoma and indolent lesions observed in *Lgr5 Apc Huwe1* (left panel) and *Lgr5 Apc Huwe1 Mcl1*^{het} (right panel) mice. Scale bars = 100 μm.

C Image of intestines from *Lgr5 Apc Huwe1* (left panel) and *Lgr5 Apc Huwe1 Mcl1*^{het} (right panel) mice. Adenomas indicated by red arrows and indolent lesions by black arrows. Note the decreased ration of adenomas/indolent lesions observed in *Mcl1*^{het} intestines. Scale bars = 100 μm.

© 2016 Cancer Research UK Beatson Institute

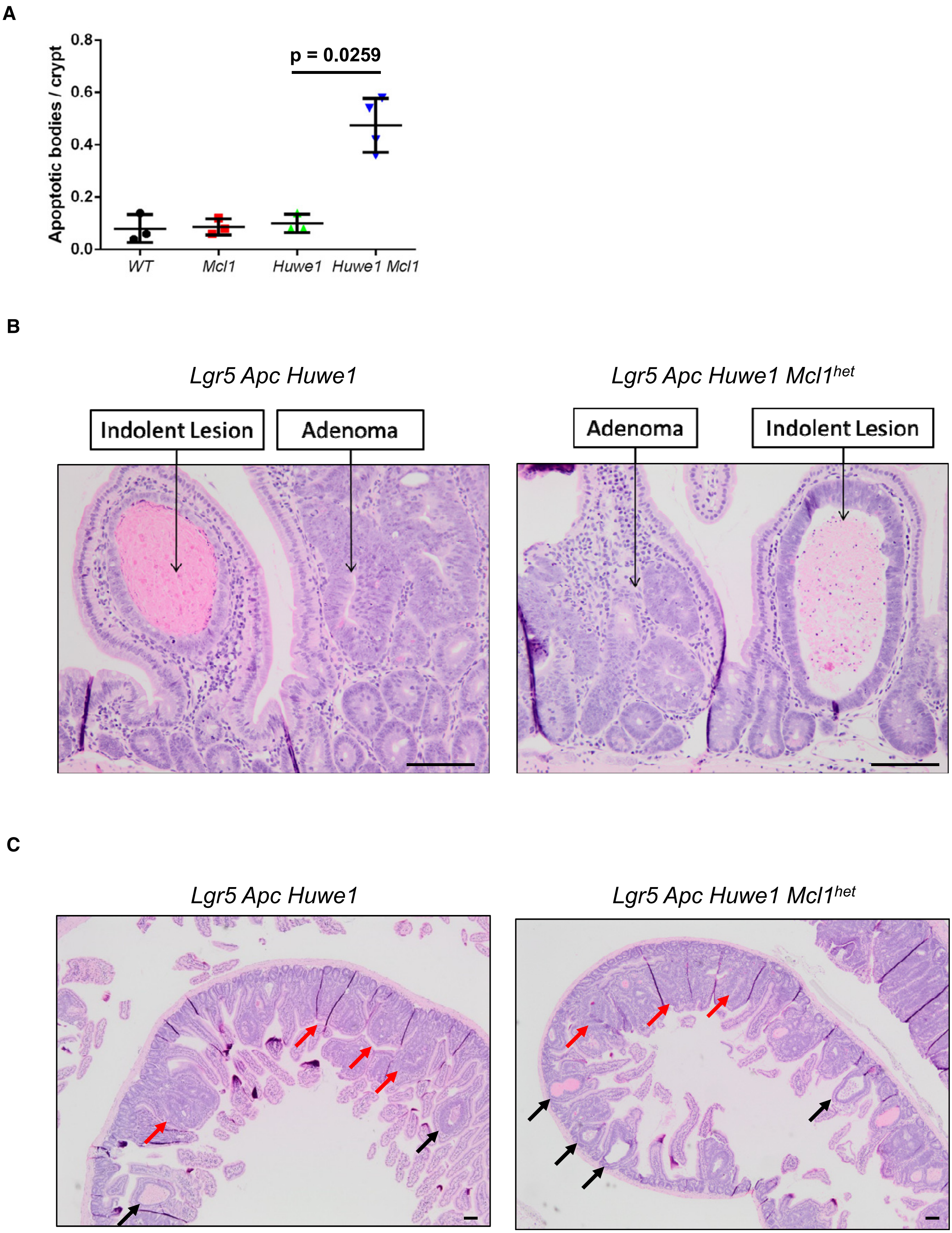
A
p = 0.0259
Apoptotic bodies / crypt
0.0
0.2
0.4
0.6
0.8
WT
Mcl1
Huwe1
Huwe1 Mcl1
B
Lgr5 Apc Huwe1
Lgr5 Apc Huwe1 Mcl1het
Indolent Lesion
Adenoma
Adenoma
Indolent Lesion
C
Lgr5 Apc Huwe1
Lgr5 Apc Huwe1 Mcl1het

Figure EV6.
