# Supplementary figures and images for: HUWE1 is a critical colonic tumour suppressor gene that prevents MYC signalling, DNA damage accumulation and tumour initiation
Source: EMBO Mol Med. 2016 Dec 22;9(2):181–97. doi: 10.15252/emmm.201606684 (PMC5286368; doi:10.15252/emmm.201606684)

Figure 3A

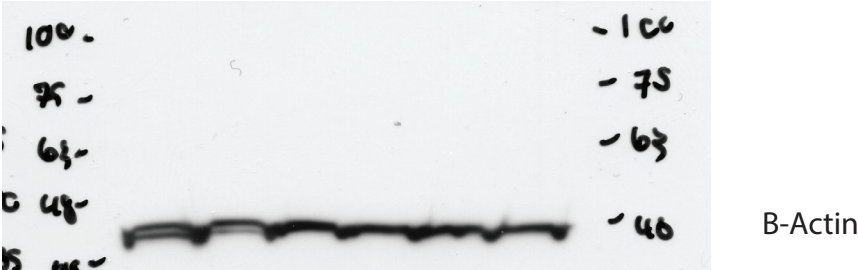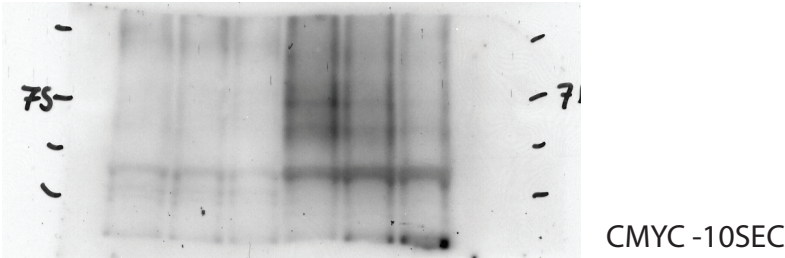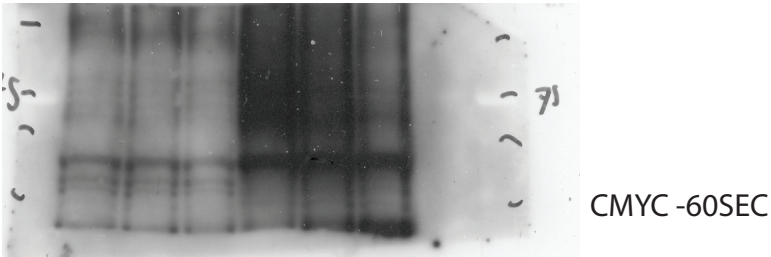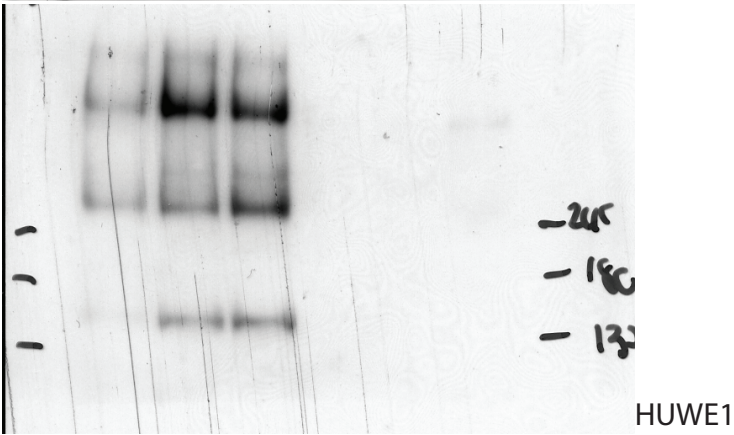

Figure 3D

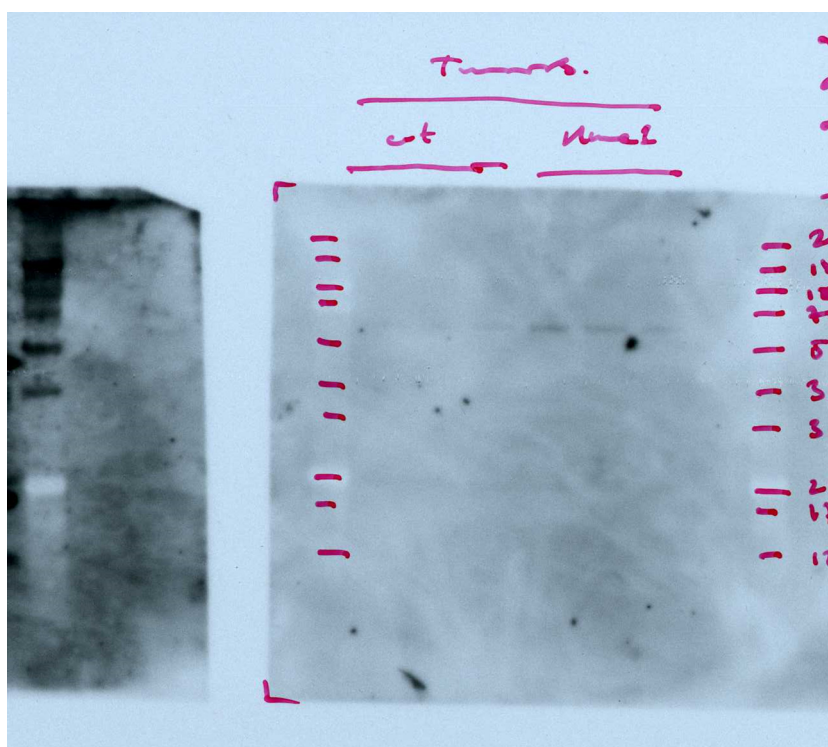

c-Myc

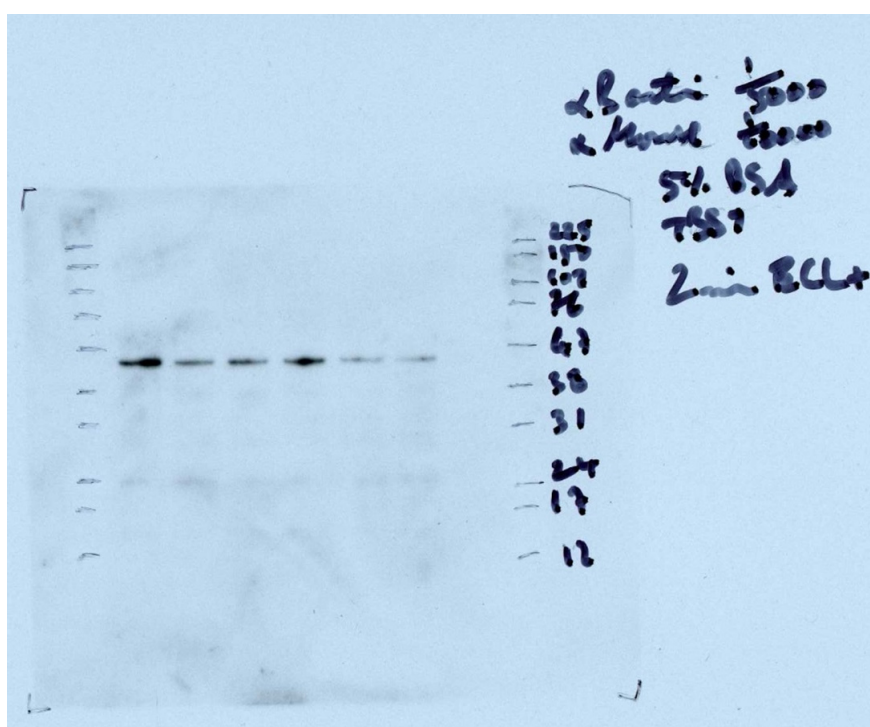

Actin

Supplement: Supplementary file 4 — Source Data for Figure 3 [file EMMM-9-181-s003.pdf]

Figure 5A

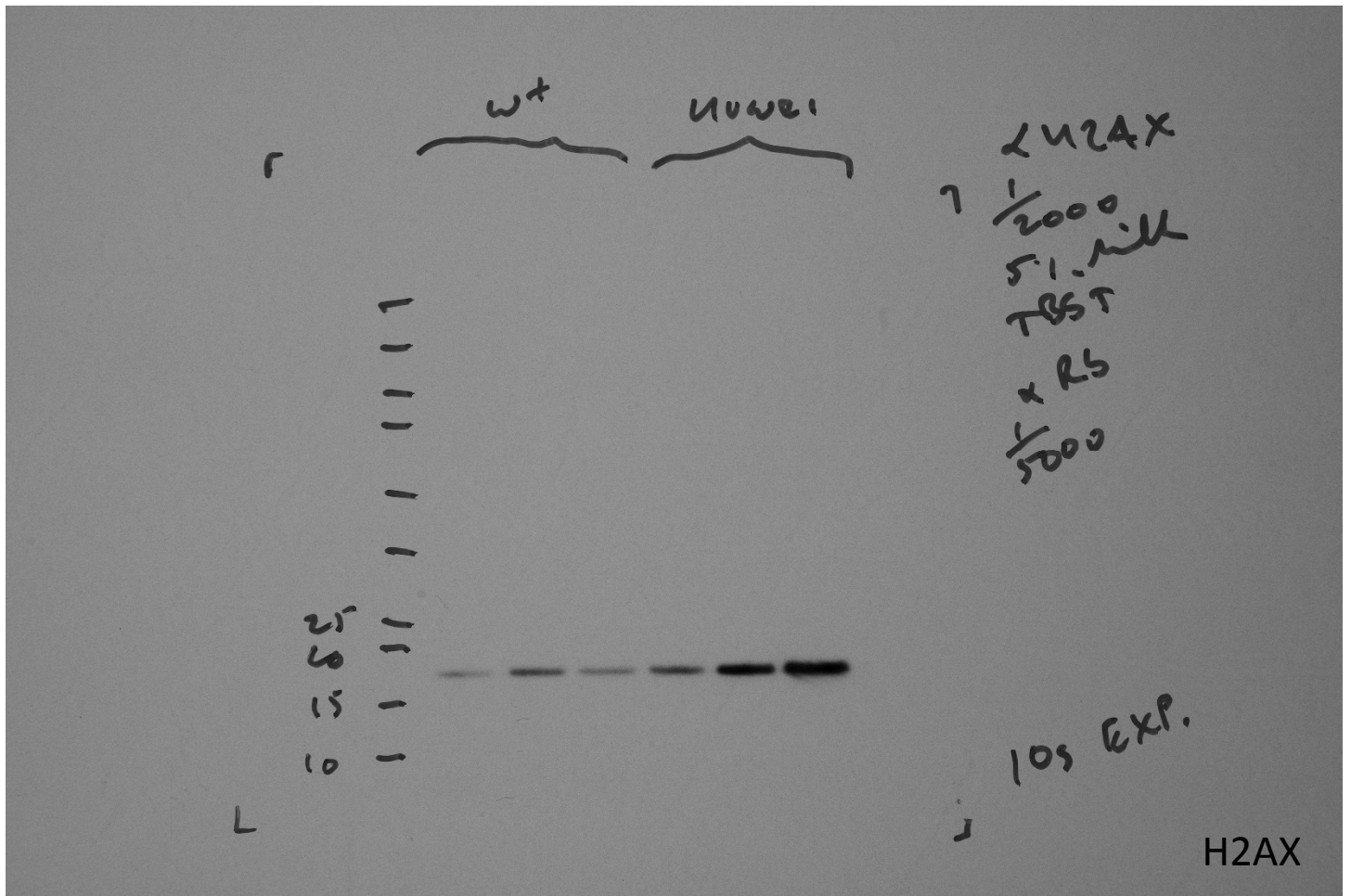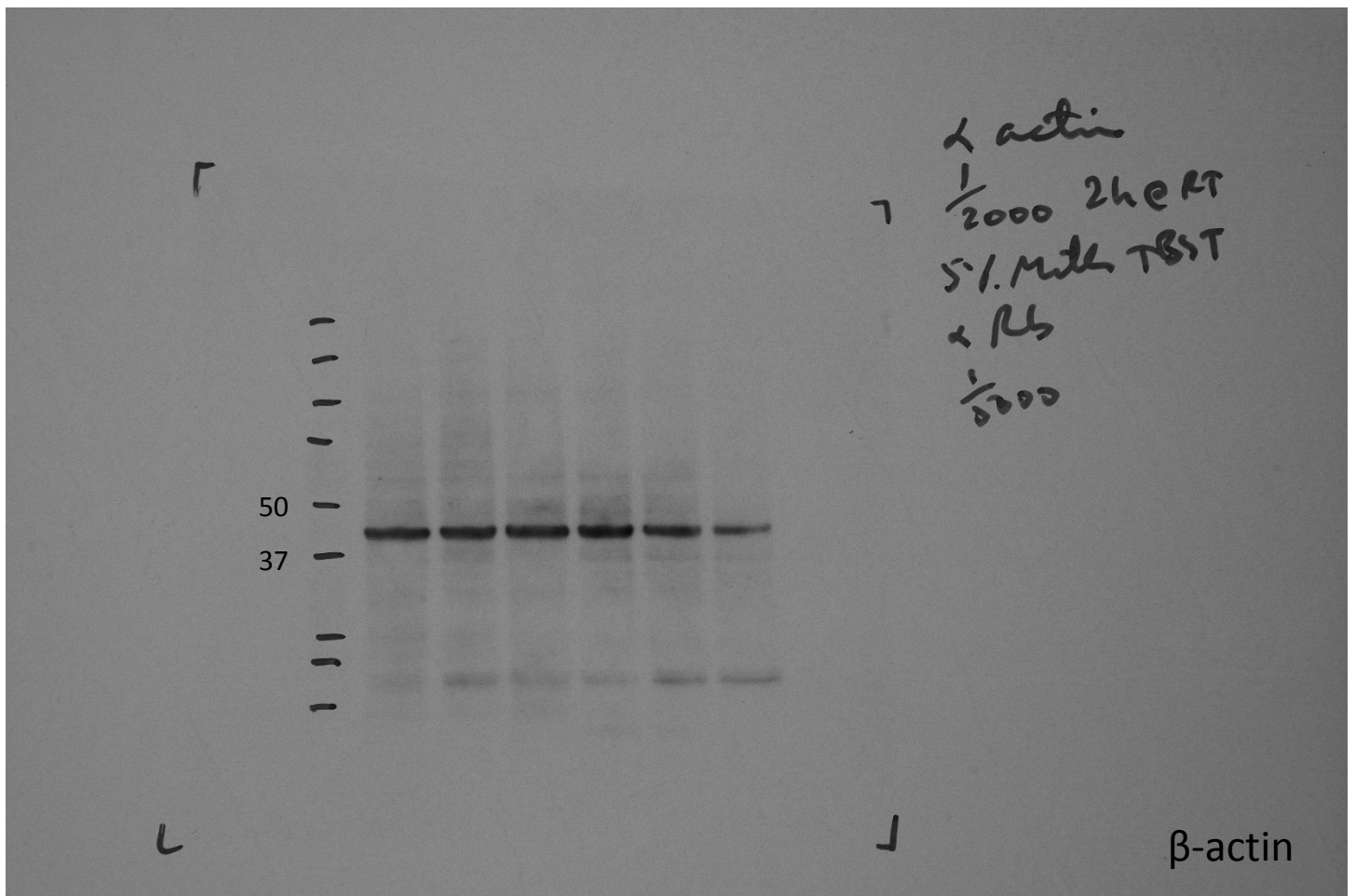

Figure 5C

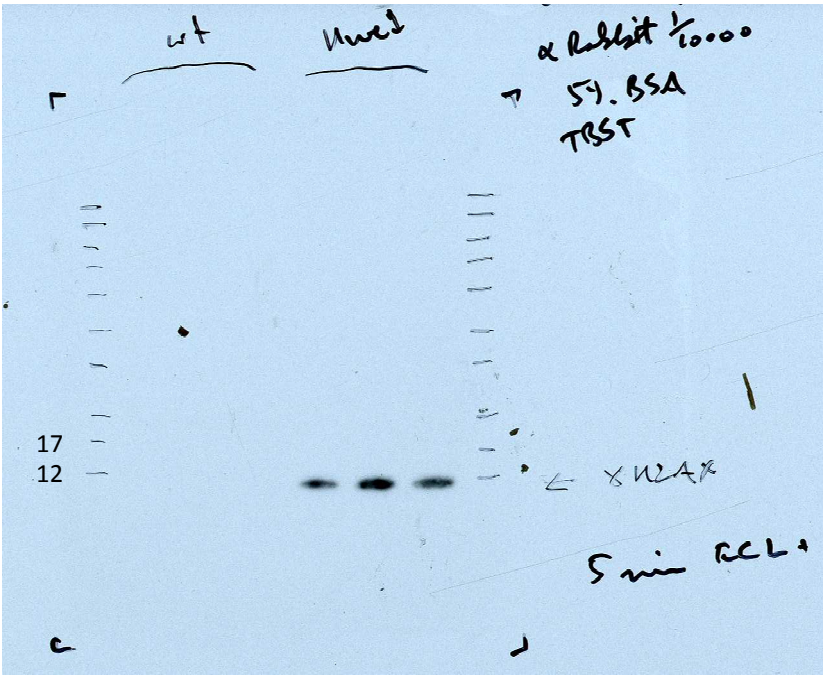

γ-H2AX

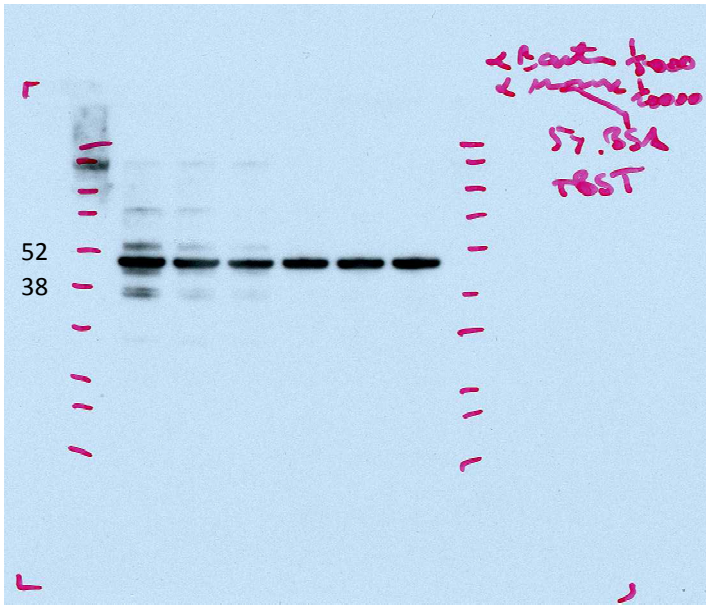

β-actin

Supplement: Supplementary file 5 — Source Data for Figure 5 [file EMMM-9-181-s004.pdf]

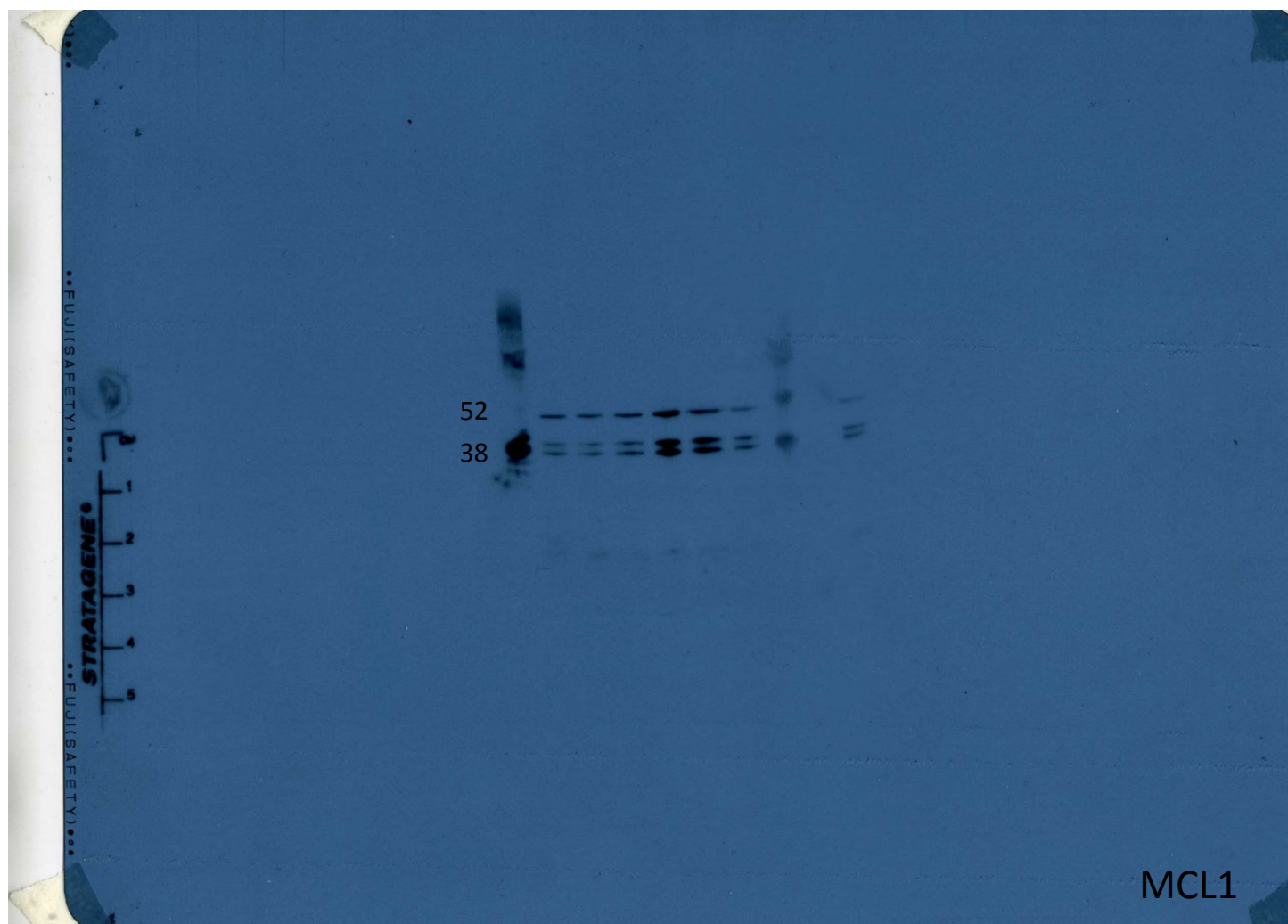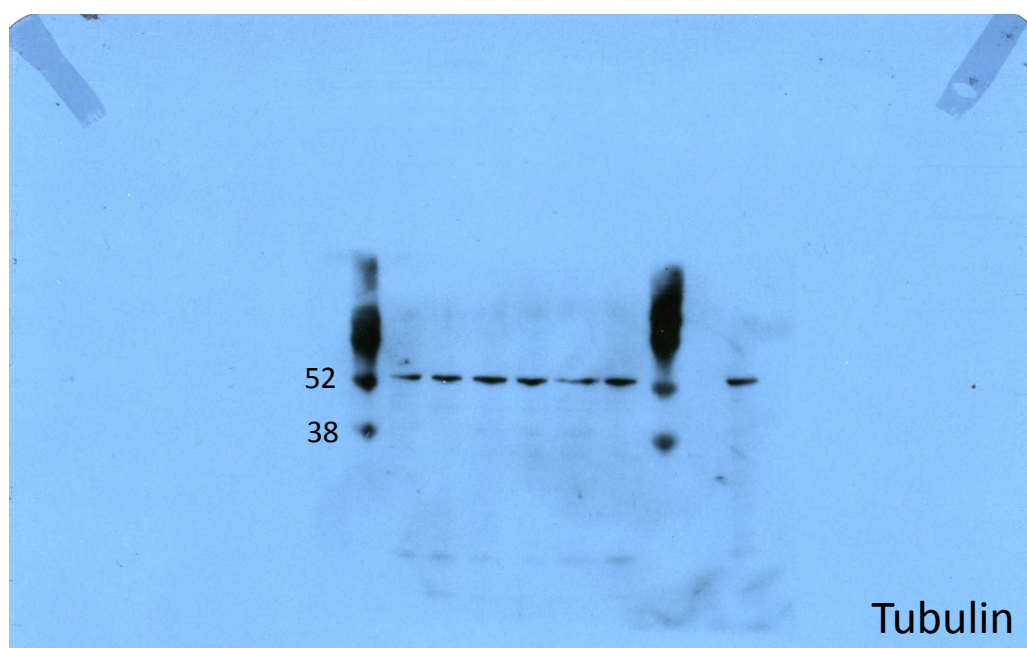

Supplement: Supplementary file 6 — Source Data for Figure 8 [file EMMM-9-181-s005.pdf]
